# Tumor microenvironment–activated Zn//MnO_2_ battery for sustained and local electrochemical immunotherapy

**DOI:** 10.1126/sciadv.adu1647

**Published:** 2025-05-28

**Authors:** Xiaoran Ding, Xiaoteng Jia, Hongming Yuan, Daxin Pang, Hualu Zhao, Peng Sun, Fangmeng Liu, Danming Chao, Meiying Xin, Caiyun Wang, Geyu Lu, Gordon Wallace

**Affiliations:** ^1^ State Key Laboratory of Integrated Optoelectronics, JLU Region, College of Electronic Science and Engineering, Jilin University, Changchun 130012, China.; ^2^ College of Animal Sciences, Jilin University, Changchun 130062, China.; ^3^ International Center of Future Science, Jilin University, Changchun 130012, China.; ^4^ College of Chemistry, Jilin University, Changchun 130012, China.; ^5^ Department of Pediatric Neurology, Children’s Medical Center, The First Hospital of Jilin University, Changchun 130021, China.; ^6^ Jilin Provincial Key Laboratory of Pediatric Neurology, Changchun 130021, China.; ^7^ Intelligent Polymer Research Institute, Faculty of Engineering and Information Sciences, University of Wollongong, North Wollongong, NSW 2500, Australia.

## Abstract

Metal ions are widely adopted to potentiate oxidative damage-induced immunogenic cell death (ICD) and regulate immune pathways. However, current ion delivery platforms lack precise administration over distribution and retention time within the tumor microenvironment, leading to high systemic toxicity and suboptimal therapeutic efficacy. Here, local electrochemical immunotherapy is developed by implanting a tumor microenvironment–activated Zn//MnO_2_ battery, which uses body fluid as an electrolyte and endogenous glutathione as a redox mediator to promote sustained generation and localized retention of electrochemical products (Zn^2+^ and Mn^2+^). These elements trigger the ICD effect through reactive oxygen species accumulation and stimulate the STING pathway to activate immune cells effectively. Strategic placement of this battery around subcutaneous tumor sites results in a 99.6% growth inhibition rate after a 14-day treatment. This work demonstrates a powerful electrochemical tool for drug-free cancer immunotherapy, which may open an avenue for sustained and localized delivery of immune agonists.

## Teaser

An implantable battery effectively delivers metal ion agonists and activates immune responses.

## INTRODUCTION

Immunotherapy is a prominent and effective cancer treatment that restores normal antitumor immune response by reactivating and sustaining the tumor-immune cycle (*1*–*3*). Current immunotherapies, such as immune checkpoint blockade (*4*–*7*) and adaptive cell therapies (*8*–*10*), suffer from insufficient immune activation and an immunosuppressive microenvironment (*11*, *12*). Innate immune systems are capable of defense against pathogen invasion and cellular carcinogenesis (*13*). The cyclic guanosine monophosphate–adenosine monophosphate synthase (cGAS) stimulator of interferon genes (STING) has been identified as a prominent innate immune signaling pathway (*14*–*18*). Abnormal double-stranded DNA in the cytoplasm is recognized by the cGAS receptor, which promotes cascade activation of the STING pathway. This, in turn, triggers the expression and secretion of type I interferon (IFN-I) and proinflammatory cytokines, initiating an effective innate immune response to inhibit tumor growth (*19*, *20*). Growing evidence suggests that Mn^2+^ ions play a vital role in promoting cGAS-STING pathway activation. These ions can freely penetrate cell membranes and increase the affinity of cGAS for double-stranded DNA to further activate the STING pathway, thereby stimulating the maturation of dendritic cells (DCs) and enhancing tumor-specific T cell responses (*21*–*26*). Furthermore, the presence of excessive metal ions can trigger different kinds of programmed cell death through various pathways, including oxidative stress, cell membrane disruption, protein inactivation, and DNA fragmentation (*27*). Mn^2+^ ions, in particular, can induce cell apoptosis via a Fenton-like reaction and generate toxic hydroxyl radicals (·OH), which amplify oxidative stress promoting an effective immunogenic cell death (ICD) effect (*28*–*30*). However, free Mn^2+^ ions can quickly enter the circulatory system from the injection site and be rapidly eliminated. Effective delivery of the Mn^2+^ ions is essential to maximize immunosensitive functions (*31*, *32*).

Recently, various ion delivery carriers have been developed to introduce exogenous manganese ions for immune activation and tumor elimination by leveraging molecular engineering and nanobiotechnology strategies (*33*). Representative carriers include responsive Mn-based nano-agonists (*22*), such as Mn-layered double hydroxide nanoparticles (*34*, *35*), Mn nanofiber hydrogels (*36*), Mn lipid nanocomposites (*37*), and Mn polymer nanomaterials (*38*). However, the clinical development of Mn^2+^ immune agonists as immunotherapies faces challenges (*39*, *40*), including (i) the need for frequent intravenous injections due to the short retention time of nanoparticles (less than 2 days), (ii) adverse toxicities and immune-suppressive effects associated with nontargeted systemic administration, and (iii) limited accessibility to certain solid tumor sites.

Therefore, a key goal in cancer immunotherapy is to design a sustainable therapeutic delivery platform that enables precise administration to the tumor and/or tumor microenvironment (TME), prolongs ions release, and minimizes systemic toxicity. Local immunotherapy, such as implantable bioelectronic devices at tumor sites and injectable biodegradable hydrogels, offers a promising solution for eliciting tumor-specific immune responses (*41*, *42*). These methods overcome random distribution in healthy tissue to minimize side effects while enhancing efficacy. Implantable self-charging batteries and injectable fiber electronic devices have been shown to regulate the tissue microenvironment by reacting with oxygen at the cathode to release radical species, but their long-term stability in the TME is uncertain due to fluctuations in oxygen levels in biofluids (*41*, *42*). Given the complexity of the TME with variable acidity, high redox potentials, and hypoxic conditions, it is essential to rationally tailor the electrochemistry of implantable therapeutic batteries. This potent electrochemical tool may serve as an immunotherapy approach by integrating innate immune response modulation with sustained local delivery of agonists.

**Fig. 1.**
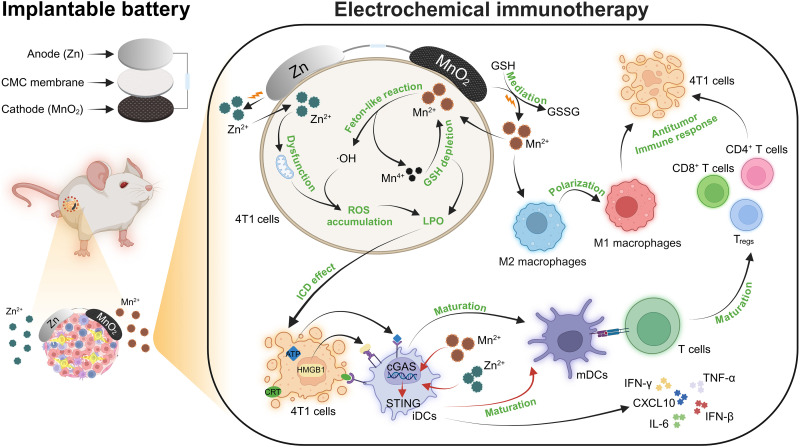
Scheme of the TME-activated Zn//MnO_2_ battery for enhancing local electrochemically mediated immunotherapy. Combined with the consumption of GSH, Zn^2+^, and Mn^2+^ generated by the battery discharge leading to an obvious accumulation of reactive oxygen species (ROS), thereby inducing the ICD effect. Mn^2+^ and Zn^2+^ also stimulate macrophage polarization and activate the cGAS-STING pathway, promoting DC maturation and T cell activation, ultimately achieving a powerful antitumor immune response. LPO, lipid peroxide; iDCs, immature dendritic cells; mDCs, mature dendritic cells.

Here, we report an implantable TME-activated Zn//MnO_2_ battery that enables continuous localized delivery of metal ions to activate the immune system. This battery uses body fluid as electrolyte and redox-active species as mediators, becoming activated in there to control the TME. This approach extends the battery life span and offers an additional charge-transfer route beyond the electrochemical interface. Compared to the unconnected battery (UB; i.e., in an open circuit state), the battery discharge facilitates greater localized generation of Mn ions through effective two-electron transfer conversion between Mn^2+^ and solid MnO_2_ accompanied by the consumption of endogenous glutathione (GSH). With the help of GSH depletion, oxidative stress imbalance is achieved through a Mn^2+^-induced Fenton-like reaction and Zn^2+^-induced accumulation in mitochondria. All these products contribute to the ICD of tumor cells, stimulating the cGAS-STING pathway, activating DCs, increasing T lymphocytic infiltration, and reversing the immunosuppressive microenvironment (shifting from M2 macrophages to M1 macrophages). This ultimately results in favorable antitumor effects, achieving a tumor growth inhibition (TGI) rate of 99.6%. Overall, this implanted battery serves as a localized therapeutic device that offers a unique approach to regulate the TME and activate immunity, which has a high loading capacity of metal ions (150 mg/kg), sustained and localized delivery of immune agonists, drug-free single-session treatment, and strong growth inhibitory effect (99.6%) (Fig. 1 and tables S1 and S2). This positions it as a promising electrochemical immunotherapy approach for drug-free tumor therapy, distinguishing it from nanomedicine-based metal ion delivery platforms.

## RESULTS

### Design and electrochemistry of the TME-activated Zn//MnO_2_ battery

The TME-activated battery is composed of a metallic Zn foil anode and an α-manganese dioxide (α-MnO_2_) cathode (fig. S1) (*43*, *44*), both of which exhibit excellent biocompatibility. When this battery is implanted in the subcutaneous region of mice, it uses body fluid as the electrolyte and harnesses GSH from the TME as an electron shuttle between the electrode and reactants, thereby enhancing discharge capacity and prolonging battery lifetime.

At the anode

$$\begin{array}{c} \text{Zn}\;-\;2\;e^{-}\;\to \;{\text{Zn}}^{2+} \end{array}$$ (1)

At the cathode

$$\begin{array}{c} {\text{MnO}}_{2}+2\;\text{GSH}+2\;H^{+}\to \text{GSSG}+{\text{Mn}}^{2+}+2\;H_{2}O \end{array}$$ (2)

$${\text{MnO}}_{2}+e^{-}+{\text{4H}}^{+}\to {\text{Mn}}^{3+}+2\;H_{2}O$$ (3)

$${\text{MnO}}_{2}+2\;e^{-}+4\;H^{+}\to {\text{Mn}}^{2+}+2\;H_{2}O$$ (4)

**Fig. 2.**
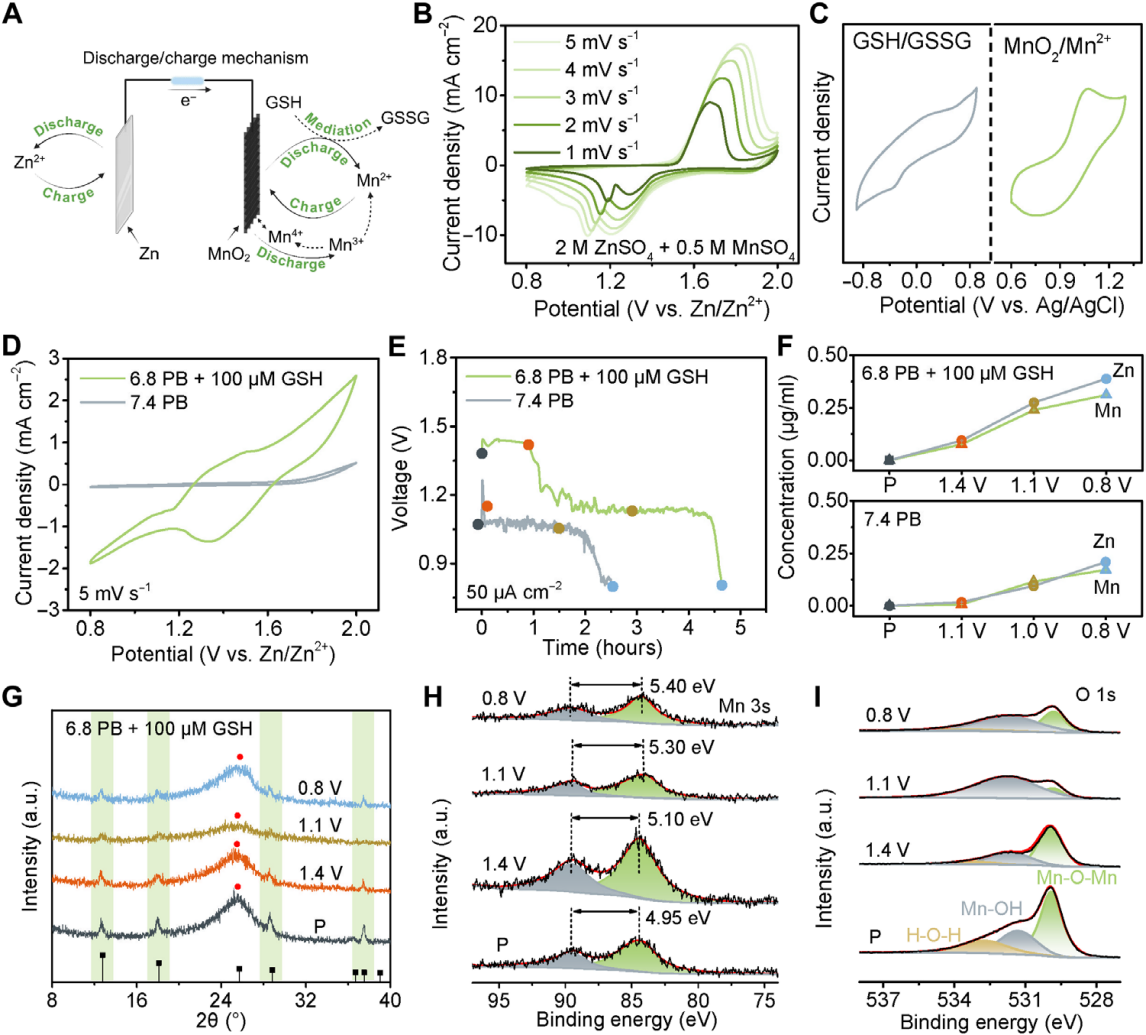
Electrochemical profiles of the TME-activated Zn//MnO_2_ battery in the ambient environment. (**A**) Schematic diagram of its energy storage mechanism. (**B**) CV curves at different scan rates in 2 M ZnSO_4_ and 0.5 M MnSO_4_. (**C**) CV curves of 100 μM GSH in 6.8 PB (left) and 0.5 M MnSO_4_ (right) electrolytes at 5 mV s^−1^. (**D**) CV and (**E**) galvanostatic discharge curves of Zn//MnO_2_ batteries with PB (pH 7.4) and GSH-containing PB (pH 6.8) electrolytes. (**F**) Mn and Zn concentrations in the electrolytes measured by ICP at different stages of discharge. (**G**) Normalized XRD and XPS patterns of (**H**) Mn 3s and (**I**) O 1s of the MnO_2_ cathode in 100 μM GSH in PB (pH 6.8) at different states of discharge. a.u., arbitrary units.

During the discharge process, the GSH mediator reduces solid MnO_2_ due to a lower redox potential compared to MnO_2_/Mn^2+^. This spontaneous chemical process endows the battery with the capability to deplete GSH while providing additional redox capacity. Subsequently, the oxidized glutathione (GSSG) accepts electrons at the electrode, converting it back to GSH and completing the mediation cycle. Meanwhile, a cyclic loop involving soluble Mn^2+^ and solid MnO_2_, facilitated by a two-electron reaction, is established at the cathode within the TME (Fig. 2A). It is worth noting that the oxygen reduction reaction (ORR) at the cathode may occur when oxygen receives electrons catalyzed by MnO_2_. To verify this, a rotating disk electrode was used to conduct linear sweep voltammetry (LSV) on the MnO_2_ electrode in an oxygen-saturated phosphate buffer (PB) electrolyte (pH = 6.8). As shown in fig. S2, no obvious half-wave potential was observed, and the limiting current density remained low at 0.6 mA cm^−2^, indicating that no notable ORR occurred at the cathode (*45*–*47*). In addition, electron paramagnetic resonance (EPR) showed the same result (fig. S3).

To gain a comprehensive understanding of the redox process, the Zn//MnO_2_ battery was initially studied in 2 M ZnSO_4_ and 0.5 M MnSO_4_ electrolytes (pH 4.6). The ZnSO_4_ aqueous electrolytes enable H^+^ intercalation, contributing to the capacity. Mn^2+^ is introduced as an electrolyte additive to further enhance capacity through the deposition and dissolution of Mn^2+^. Figure 2B describes the cyclic voltammogram (CV) curves of Zn//MnO_2_ at different scan rates within the voltage window of 0.8 to 2.0 V. The peaks at ~1.2 and 1.3 V (versus Zn^2+^/Zn) can be attributed to the intercalation/deintercalation of Zn^2+^ and/or H^+^. The proportion of the capacitive contribution grew progressively from 20 to 38% as the scan rate increased from 1 to 5 mV s^−1^ (fig. S4). The galvanostatic charge-discharge curves showed that the specific capacity decreased gradually from 260 to 93 μAh cm^−2^ with increased current densities (fig. S5). During battery discharge, the MnO_2_ cathode is reduced to MnOOH, resulting in the production of Mn^2+^ and MnO_2_ via a disproportionation reaction.

We further explored the electrochemistry using PB (pH = 7.4) to simulate normal tissue conditions and PB (pH = 6.8) with GSH (100 μM) to mimic tumor conditions. This relatively low concentration of GSH was selected because cancer cells undergo continuous lysis and release intracellular GSH during the treatment process, leading to dynamic changes in its levels. Considering extracellular GSH concentrations (at least 2 to 20 μM) and intracellular levels (2 to 10 mM), we chose a relatively small value of 100 μM as the experimental condition for electrochemical experiments. The thermodynamic feasibility of the GSH redox mediator was confirmed by its much lower redox potential compared to the equilibrium potential of MnO_2_/Mn^2+^ (Fig. 2C). Furthermore, the ultraviolet-visible spectra of 2-nitro-5-thiobenzoic acid solution showed a decreased absorbance at 412 nm after the addition of MnO_2_, attributed to the exhaustion of GSH (fig. S6). Then, the CV curves of Zn-MnO_2_ cells were first investigated (Fig. 2D). This battery demonstrated a much lower current response with no obvious redox peaks in the PB electrolyte (pH = 7.4). In contrast, two redox peaks were observed in the GSH-containing PB electrolyte, attributed to the reduction of solid MnO_2_ and the GSH/GSSG redox mediation. This can be used to explain discharge behavior (Fig. 2E). The discharge voltage dropped to 0.8 V after 2.5 hours in the PB electrolyte (pH = 7.4) (*48*). Two discharge plateaus and an 84% increase in discharge times (4.6 hours) were shown for the battery in the GSH-containing PB electrolyte. It is worth noting that the battery using GSH-containing PB electrolyte exhibited a higher plateau voltage above 1.45 V due to the MnO_2_ dissolution reaction, which was absent in the PB electrolyte. These results demonstrated that the presence of GSH improved battery performance by facilitating the dissolution-deposition of MnO_2_/Mn^2+^. The constant-resistance (100 kilohm) discharge behavior of the Zn//MnO_2_ cell also confirmed its long-lasting (5 days to 0.8 V) discharging capability in the GSH-containing PB electrolyte (fig. S7).

The Mn and Zn ion concentrations in the electrolyte were determined using inductively coupled plasma optical emission spectrometry (ICP-OES) (Fig. 2F). The concentration of each reached a maximum at a discharge voltage of 0.8 V. A lower concentration of Mn ion was observed in the phosphate-buffered saline (PBS) electrolyte (0.8 V) compared to the GSH-containing PB electrolyte (1.1 V) after the same discharging time of 2.5 hours. In addition, the generation of Mn ion was lower than Zn ion, caused by the generation of Mn^3+^ during the discharge process. Mn^3+^ undergoes a disproportionation reaction to form Mn^2+^ and Mn^4+^. In a moderately acidic environment, Mn^4+^ reacts with H_2_O to produce MnO_2_, which reduces the concentration of Mn ions in the solution. Meanwhile, this process also endowed the battery with the ability to discharge for a longer period. Collectively, these findings provide compelling evidence that the Zn//MnO_2_ battery can be activated in the TME to enable the prolonged and sustained generation of Zn and Mn ions during the discharge process

$$2\;{\text{Mn}}^{3+}\to {\text{Mn}}^{2+}+{\text{Mn}}^{4+}$$ (5)

$${\text{Mn}}^{4+}+2\;H_{2}O\to {\text{MnO}}_{2}+4\;H^{+}$$ (6)

To reveal the reaction process and charge storage mechanism, the structural evolution of the MnO_2_ cathode during the discharge process in the GSH-containing PB electrolyte was investigated via ex situ measurements (Fig. 2G). No shift in diffraction peak or other peaks was observed, and the damping of the α-MnO_2_ signals suggests a solid/liquid reaction of MnO_2_ to Mn^2+^. The x-ray photoelectron spectroscopy (XPS; Fig. 2H) results displayed a doublet peak of Mn 3s with spin-energy splitting (Δ*E*) values of 4.95 eV at pristine state, 5.10 eV at 1.4 V, 5.30 eV at 1.1 V, and 5.40 eV at 0.8 V. These findings reveal that the average Mn valence states decreased from 3.38 (pristine state) to 2.87 (0.8 V). The core-level spectra of O 1s displayed peaks corresponding to the Mn─O─Mn (529.9 eV) bond for MnO_2_, Mn─OH (531.3 eV) for MnOOH, and H─O─H (532.0 eV) for residual water, respectively (Fig. 2I) (*49*).

### Battery-induced microenvironmental modulation and cell death

Given the fundamental condition for tumor survival, regulating the TME shows great potential in suppressing cancer growth. The Zn//MnO_2_ battery modulates the TME through discharge and chemical reactions (H^+^ and GSH), yielding metal ions (Zn^2+^, Mn^2+^, and Mn^3+^). First, excessive accumulation of Zn ions in cells disrupts mitochondria function. This leads to electron leakage from the electron transport chain and an increase in reactive oxygen species (ROS) levels. Excessive ROS damages mitochondrial membrane lipids, proteins, and DNA, compromising structural integrity and ultimately resulting in the loss of mitochondrial membrane potential. Second, Mn^2+^ can undergo a Fenton-like reaction with H_2_O_2_, generating ·OH, OH^−^, and Mn^4+^. This process causes an increase in ROS content and a rise in pH in the microenvironment. Notably, the accumulation of ROS along with the reduction of Mn^4+^ leads to a decrease in GSH levels. The hydrolysis of Mn^3+^ further reduces the acidity of the microenvironment. In addition, the discharge current promotes the conversion of H_2_O_2_ to ·OH, further increasing ROS levels

$${\text{Mn}}^{2+}+H_{2}O_{2}\to {\text{Mn}}^{4+}+{\text{OH}}^{-}+\cdot \;\text{OH}$$ (7)

**Fig. 3.**
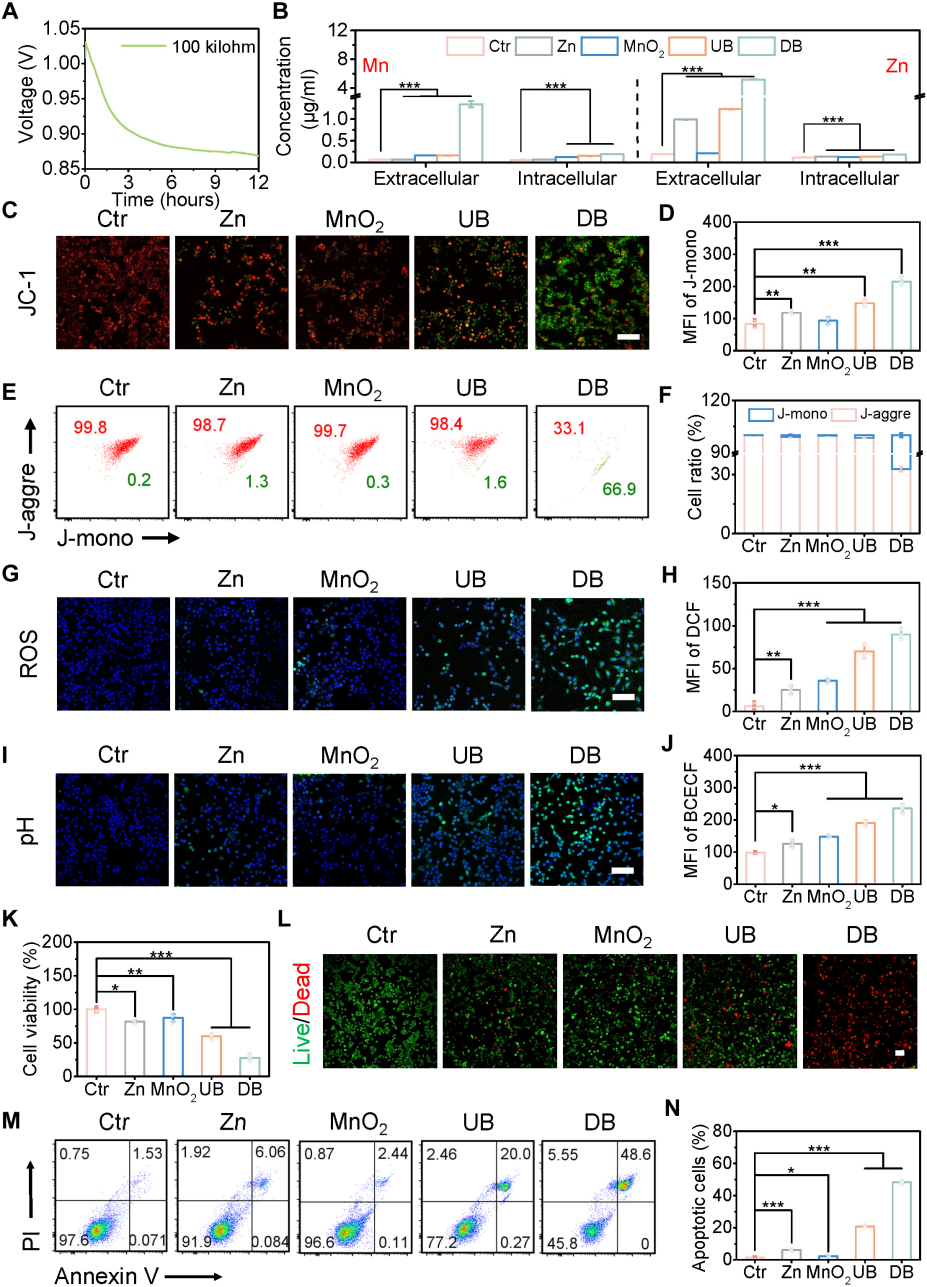
Battery-mediated microenvironment regulation and toxicity evaluation on 4T1 cells treated with various groups. (**A**) The discharge curve of Zn//MnO_2_ battery in Dulbecco’s modified Eagle’s medium (DMEM) medium. (**B**) The extracellular and intracellular concentrations of Zn and Mn ions. (**C**) Fluorescence images of ΔΨm using the JC-1 probe. (**D**) Quantification of JC-1 monomer expression. (**E**) FCM analysis and (**F**) quantitative analysis of ΔΨm. (**G**) Representative confocal images and (**H**) quantitative fluorescence intensities of ROS generation. (**I**) Fluorescence images and (**J**) corresponding quantitative intensity of cell-permeant pH changes detected by BCECF AM. (**K**) Cell viability of 4T1 cells. (**L**) Confocal microscopy images of calcein AM/PI–stained 4T1 cancer cells. (**M**) Flow cytometric apoptosis analysis and (**N**) quantification of apoptotic results of annexin V–FITC/PI–stained 4T1 cells. MFI, mean fluorescence intensity. All data are expressed as means ± SD (*n* = 3), and the differences were assessed by one-way analysis of variance (ANOVA) followed by Dunnett’s multiple comparisons tests. *P < 0.05, **P < 0.01, and ***P < 0.001. Scale bars, 100 μm.

First, the appropriate resistance value was selected (fig. S8). The battery with a 100-kilohm resistor exhibited a discharge plateau (0.86 V; Fig. 3A and fig. S9A) and metal ion generation (intracellular Zn: 66.3 ng/ml, extracellular Zn: 3.2 μg/ml, intracellular Mn: 56.3 ng/ml, and extracellular Mn: 1.6 μg/ml; fig. S9, B to E). As the resistance value increased, the discharge plateau gradually increased, ion release decreased, and cytotoxicity was reduced accordingly (fig. S9F). In contrast, decreasing the resistance value led to high ion release, increased cytotoxicity, and shortened battery life. Considering the content of metal ions and the therapeutic effect, a resistance value of 100 kilohm was selected for all subsequent experiments.

Subsequently, we have meticulously explored the dynamic regulatory effects of single electrode (Zn electrode and MnO_2_ electrode), UB, and discharging battery (DB) on the TME. As shown in Fig. 3B, compared to the control group, after coculture with different treatments, noticeable differences in intracellular and extracellular concentrations of Zn and Mn were observed because of the chemical reaction of the electrode and/or the presence of discharge current. These differences can affect on the microenvironment to varying degrees. Therefore, the changes in intracellular mitochondrial membrane potential (ΔΨm) of 4T1 cells after incubated with different treatments were first measured by using a JC-1 fluorescent probe, with green fluorescence intensity indicating the level of damage. As shown in Fig. 3 (C and D) and fig. S10, the MnO_2_ electrode showed almost no green fluorescence compared to the control group, indicating normal mitochondria function. In contrast, the Zn electrode group and the UB group exhibited a greater reduction in mitochondrial membrane potential due to the release of Zn ions (intracellular: 137.3 ng/ml) from electrode chemical reaction (Fig. 3B). It is worth noting that tumor cells treated with the DB group absorbed the highest amount of Zn ions (184.0 ng/ml), resulting in the greatest mitochondrial damage. The variations in ΔΨm were further confirmed by flow cytometry (FCM, CytoFlex, Beckman) analysis (Fig. 3, E and F).

The intracellular ROS level was investigated using a 2,7-dichlorofluorescein diacetate (DCFH-DA) probe, and the intensity of green fluorescence is positively correlated with the level of ROS. Compared to the control group, the single-electrode and UB groups increased the levels of ROS to 4.0-, 5.8-, and 11.3-fold (Fig. 3, G and H, and fig. S11), corresponding to the high concentrations of Mn and Zn ions generated by chemical reactions. It is worth noting that the DB-treated 4T1 cells presented the highest ROS level compared to other treatments. The production of ROS under different treatments was also verified by flow cytometry (fig. S12). In addition, the discharge current may play a role in regulating the microenvironment. To verify this hypothesis, we used electrochemically inert Pt and pure carbon cloth electrodes to observe ROS changes within the TME. An optimized dc voltage (860 mV) was applied, corresponding to the voltage plateau (0.86 V) of Zn//MnO_2_ battery with a 100-kilohm resistor incubated with 4T1 cells (Fig. 3A). Compared to the control group, the levels of ROS increased to 13.2-fold (fig. S13), which was lower than the 14.5-fold increase in the DB group (Fig. 3H). As oxygen cannot accept electrons to generate ROS (LSV and EPR results; figs. S2 and S3), this ROS increase in the inert electrode (IE) group can be attributed to the discharge current and the presence of H_2_O_2_ in TME.

To detect the change of intracellular pH, the 2′,7′-bis-(2-carboxyethyl)-5-(and-6)-carboxyfluorescein probe was used to incubate with 4T1 cells. As shown in Fig. 3 (I and J) and fig. S14, the intracellular pH values of all experimental groups remarkably increased to varying degrees, caused by the effect of metal ions and/or discharge current. The strongest green fluorescence was observed in DB-treated 4T1 cells, with an intensity approximately 2.5-fold that of the control group. The pH values of the treated 4T1 cell supernatant were measured at different time points (fig. S15), which showed similar trends as observed in the fluorescence results. GSH content in the microenvironment was studied using 5,5′-dithiobis(2-nitrobenzoic acid) (DTNB). In contrast to the patterns observed in the other indicators, the GSH contents in the single-electrode, UB, and DB groups were remarkably lower than in the control group, further confirming the up-regulated ROS level (fig. S16).

In addition, to observe the dynamic regulation of the microenvironment by the DB group, we also assessed changes in the microenvironment at 1, 3, and 6 hours (fig. S17). After 3 hours of incubation, the intracellular concentration of Zn was 39.3 ng/ml, while the intracellular concentration of Mn was 18.7 ng/ml. These accumulated metal ions had an obvious regulatory effect on the microenvironment, manifested in the remarkably increased green fluorescence intensity in both pH and ROS. As the incubation time increased, the generation of metal ions also rose, further enhancing the regulatory effects of the battery on the microenvironment, including an increase in pH and ROS levels and a decrease in GSH levels.

Increasing evidence suggests that microenvironment regulation, such as an increase in the ROS level, affects cell viability by inducing lipid peroxidation. We investigated the in vitro cytotoxicity effect of Zn//MnO_2_ battery toward 4T1 cells using standard methylthiazolyldiphenyl-tetrazolium bromide (MTT) assay (Fig. 3K). The Zn electrode and MnO_2_ electrode groups showed good biocompatibility with cell viability of ≥80%, indicating that the single-electrode treatment was ineffective under TME conditions. In contrast, the cell viability decreased to 59.9% in the UB group. It is worth noting that cell death was remarkably enhanced using DB, ascribed to the discharge current and the highest ion production inducing the strongest ROS levels (*41*, *50*). All the aforementioned phenomena also appeared in the MTT experiments on cervical (HeLa), melanoma (B16-F10), and liver (HepG2 and Hepa 1-6) cancer cell lines (fig. S18, A to D). In addition, the cell scratch experiments also showed that the DB group achieved a stronger cell migration inhibition than other groups (fig. S19).

The in vitro tumor therapeutic impact induced by the Zn//MnO_2_ battery was further confirmed using a calcein acetoxymethyl ester (calcein-AM)/propidium iodide (PI) costaining assay (Fig. 3L and fig. S20). Consistent with the MTT tests, the DB group induced the highest percentage of 4T1 cell death, as reflected by the strongest red fluorescence signal. In addition, DB treatment reached an apoptosis rate of 48.6% as shown in the flow cytometry, much higher than the UB treatment (20.0%; Fig. 3, M and N, and fig. S21). These results suggest that the DB group induces tumor cell death by aggravating oxidative damage. To explore the cell type selectivity of the Zn//MnO_2_ battery, we selected fibroblast (3T3) and myocardial (HL-1) cell lines to evaluate the cell viability after being treated with various groups. As shown in fig. S18 (E and F), the Zn//MnO_2_ battery induced cytotoxicity of less than 20% in these environments. This can be explained as the amount of Zn and Mn produced in the normal cellular environment is much lower than that in the TME (fig. S22), which suggests minimal damage to normal tissue.

### Battery-induced ICD and in vitro antitumor immune response

**Fig. 4.**
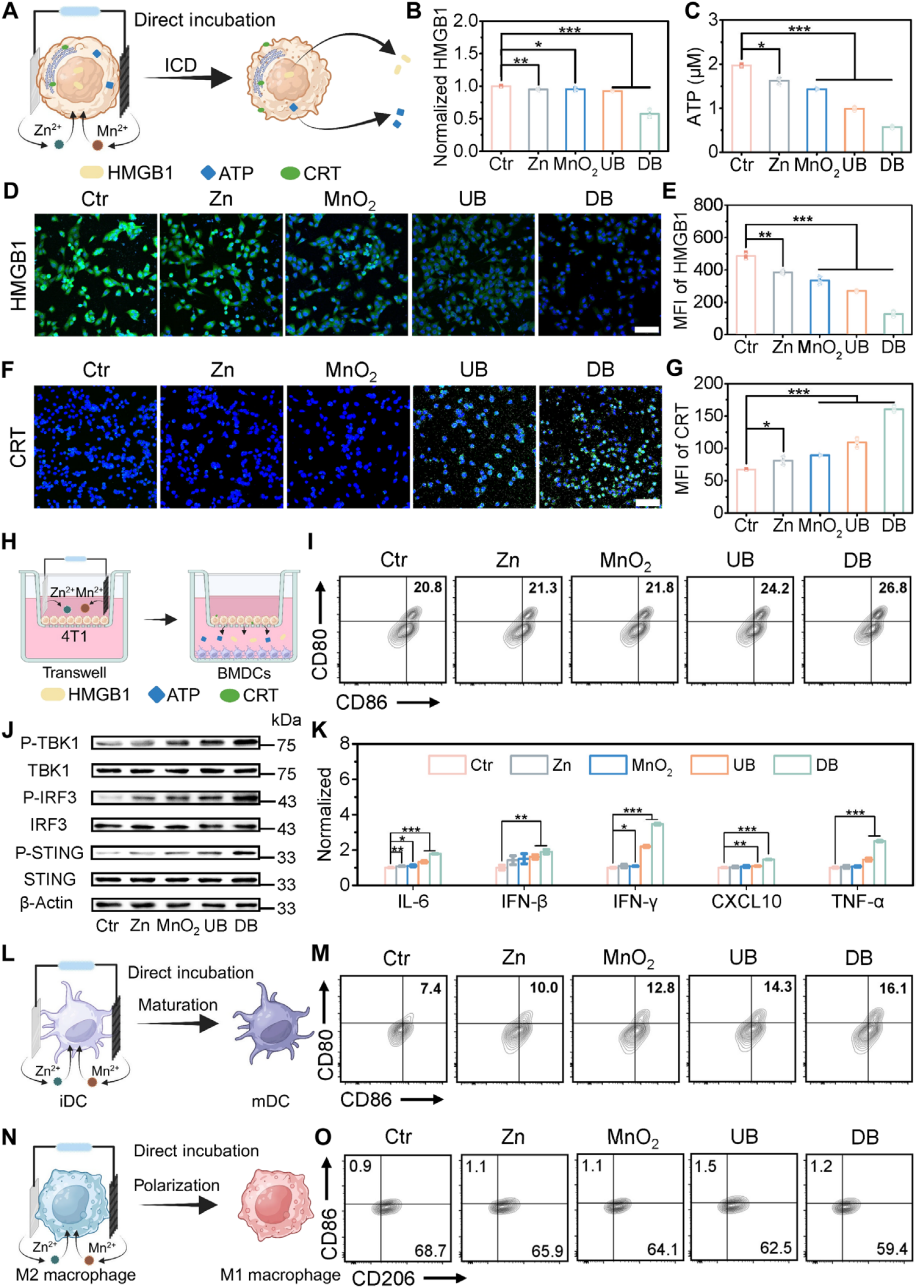
Battery-mediated ICD and tumor immunosuppressive microenvironment reversal. (**A**) Schematic illustration of battery-induced ICD in 4T1 cells. Changes in intracellular levels of (**B**) HMGB1 and (**C**) ATP after various treatments. (**D**) Confocal microscopy images and (**E**) corresponding quantitative intensity analysis of HMGB1. Scale bar, 100 μm. (**F**) Confocal microscopy images and (**G**) corresponding quantitative intensity analysis of CRT exposure on the tumor cell surface. Scale bar, 100 μm. (**H**) Schematic illustration of BMDCs coincubated with different treated 4T1 cell groups by the Transwell system. FCM analysis of (**I**) BMDC maturation stimulated by 4T1 cell groups or (**M**) direct incubation treatments. (**J**) Western blot analysis of STING, P-STING, IRF3, P-IRF3, TBK1, P-TBK1, and β-actin pathways in BMDCs incubated with various treatments. (**K**) The secretion level of cytokines by BMDCs incubated with various treatments. (**L**) Schematic illustration of battery-induced BMDC maturation. (**N**) Schematic illustration of battery-induced polarization of M2 macrophages into M1. mDC, mature dendritic cell. (**O**) FCM analysis of the M1- and M2-type macrophage in BMDMs. All data are expressed as mean ± SD (*n* = 3), and the differences were assessed by one-way ANOVA followed by Dunnett’s multiple comparisons tests. *P < 0.05, **P < 0.01, and ***P < 0.001.

Excessive ROS can induce the ICD effect by oxidizing proteins, lipids, and DNA, activating the endoplasmic reticulum stress response, and releasing calcium ions. During this process, changes in damage-associated molecular patterns (DAMPs) occur, including the high expression of calreticulin (CRT) on the dying tumor cell surfaces and the release of signaling molecules, such as high mobility group protein 1 (HMGB1) and adenosine triphosphate (ATP) (Fig. 4A). First, we demonstrated the extracellular release of HMGB1 and ATP by measuring changes in the content of intracellular signaling molecules using an enzyme-linked immunosorbent assay (ELISA) (Fig. 4, B and C, and fig. S23). Compared with the control group, the HMGB1 and ATP in DB-treated 4T1 cells were 0.4- and 0.7-fold lower, respectively. The results from confocal laser scanning microscopy (CLSM) and western blotting (WB) showed the same decreasing trend (Fig. 4, D and E, and figs. S24 and S25). These released molecules act as a “find me” signal to facilitate the phagocytosis of apoptotic cells and stimulate specific antitumor immune effects. In addition, we evaluated the surface expression of CRT in 4T1 cells stained with an anti-calreticulin (CALR) polyclonal antibody [fluorescein isothiocyanate (FITC) conjugated] by fluorescence microscopy. The brightest green fluorescent signal in tumor cells was observed after DB treatment (Fig. 4, F and G, and fig. S26), indicating effective expression of CRT on the cell membrane surface. In contrast, other group–treated cells showed less or negligible fluorescence. All the above results revealed that the Zn//MnO_2_ battery–induced ICD in tumor cells can up-regulate immune signals.

These DAMPs can recruit DCs to process tumor-associated antigens (TAAs), thus promoting antigen presentation and subsequently eliciting an antitumor immune response. Therefore, we evaluated the stimulation of the bone marrow–derived dendritic cells (BMDCs) caused by ICD via a Transwell system. The 4T1 cells were inoculated in the upper layer of the diaphragm, while the lower layer was BMDCs (Fig. 4H). Costimulatory molecules CD80 and CD86 were chosen as the sign of BMDC maturity. Figure 4I and fig. S27A showed that after incubation with DB-treated 4T1 cells, the signals of CD80^+^CD86^+^ cells in CD11c^+^ cells were the strongest, with the maturation of BMDCs reaching 26.8%, which is 1.3-fold higher level than that of the control group.

Accumulating evidence has indicated that Zn^2+^ and Mn^2+^ can further activate the classical cGAS-STING signaling pathway, promoting the expression and secretion of IFN-I and IFN-stimulated genes to initiate innate immunity (*51*–*53*). As mentioned above, besides ICD-stimulated antitumor immunity, the Zn//MnO_2_ battery can generate Mn^2+^ and Zn^2+^ through redox reactions, which is expected to trigger an antitumor immune response by activating the STING pathway in antigen-presenting cells (such as DCs and macrophages). Therefore, we first analyzed the downstream signaling activation of the cGAS-STING pathway in BMDCs after coincubation with various treatments by Western blot assay. According to the phosphorylation status of key proteins analyzed by Western blotting, the expression of phosphorylation-stimulator of interferon genes (P-STING), phosphorylation-interferon regulatory factor 3 (P-IRF3), and phosphorylation-TANK binding kinase 1 (P-TBK1) was remarkably increased when using the Zn//MnO_2_ battery stimulation, demonstrating an up-regulated activation of the cGAS-STING pathway (Fig. 4J and figs. S28 and S29).

Then, the secretion of various types of proinflammatory cytokines following STING activation was investigated (Fig. 4K and fig. S30). The secretion of IFN-β increased 1.9-fold in DB-treated BMDCs compared with the control group, which can promote antigen presentation and support natural killer (NK) cell function. In addition, the DB group induced a more pronounced increase in IFN-γ levels, which can be generated by T lymphocytes, NK cells, and antigen-presenting cells. Meanwhile, we also investigated the secretion of interleukin-6 (IL-6), CXCL10, and tumor necrosis factor–α (TNF-α) proinflammatory cytokines. As expected, the secretion of these cytokines was remarkably enhanced with the DB group, which was 1.8-, 1.5-, and 2.5-fold higher compared with the control group, respectively. Consequently, the maturity of BMDCs after direct incubation with DB increased to 16.1% (Fig. 4, L and M, and fig. S27B), indicating that the DB group can directly stimulate DC maturation as a potential immune adjuvant.

The capability of the Zn//MnO_2_ battery to transfer the TME from M2 phenotype (anti-inflammatory) to M1 phenotype (proinflammatory) was also investigated by coincubating with bone marrow–derived macrophages (BMDMs) (Fig. 4, N and O). Single molecules CD86 and CD206 were chosen as the sign of BMDM polarization to M1 type and M2 type. After incubation with the DB group, the signals of CD86^−^CD206^+^ cells in F4/80^+^ cells were the weakest, as the percentage of M2-type BMDMs was decreased to 59.4%. The expression ratio of CD80:CD206 in the DB group was nearly 2.3 times higher than that of the control group, suggesting its effect on macrophage polarization (fig. S31). These results evidenced that the Zn//MnO_2_ battery could improve the tumor immunogenicity by releasing DAMPs after the ICD effect, activating the cGAS-STING pathway, promoting DC maturation, and reversing the immunosuppressive microenvironment.

### In vivo antitumor efficacy of the battery

**Fig. 5.**
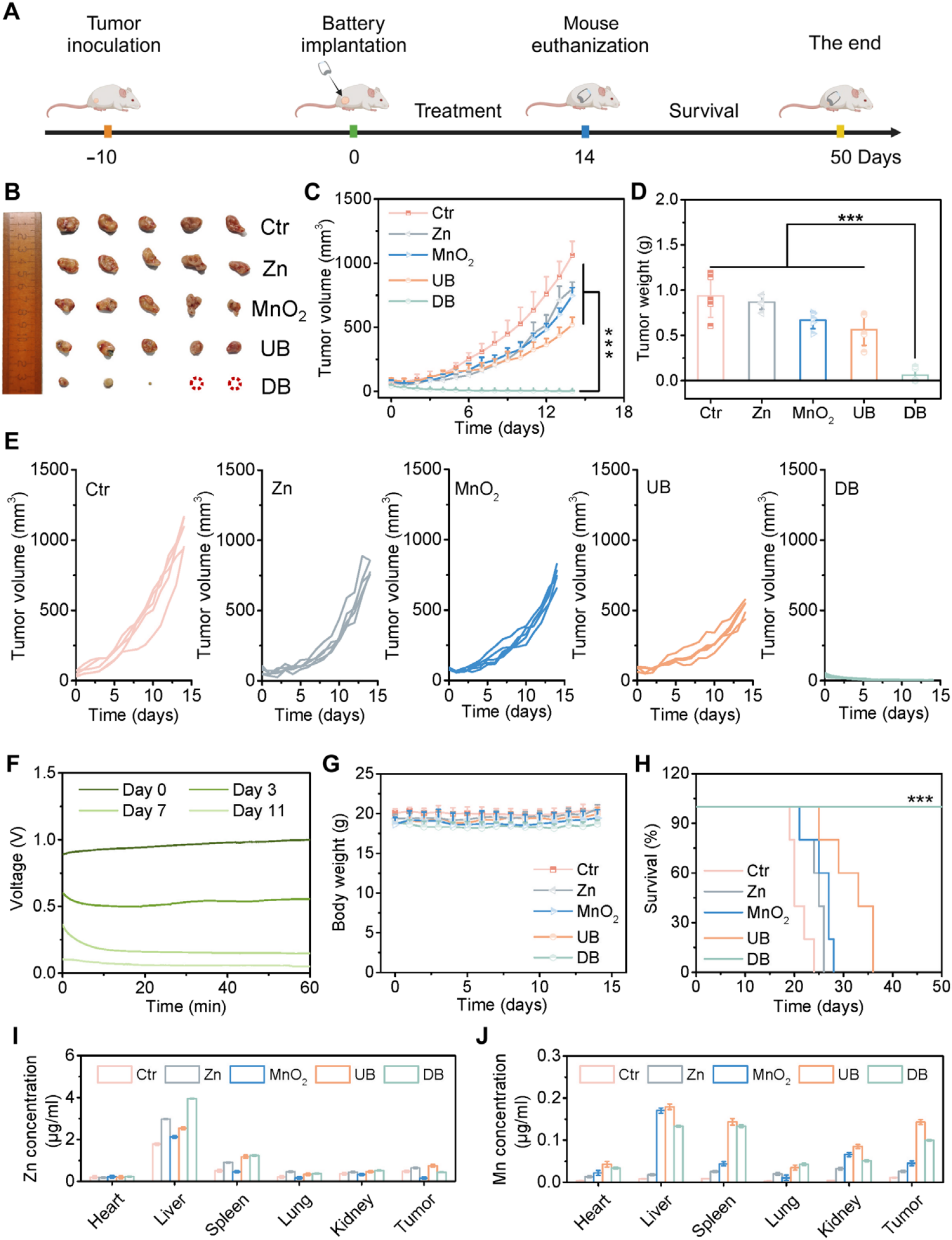
Antitumor therapeutic effect of battery in the 4T1 breast tumor model. (**A**) Schematic diagram of battery-mediated tumor treatment process. (**B**) Representative photos of tumor tissues. (**C**) Average tumor growth curves, (**D**) tumor weight curves, (**E**) tumor growth curves of each mouse, (**G**) body weight curves, and (**H**) Kaplan-Meier survival plots of mice after various treatments. (**F**) Discharging curve of an implanted battery during the treatment. The concentrations of (**I**) Zn and (**J**) Mn ions in hearts, livers, spleens, lungs, kidneys, and tumor tissues of mice after various treatments. All data are expressed as means ± SD (*n* = 5), and the differences were assessed by one-way ANOVA with Turkey’s posttest for (C) and (D) and log-rank (Mantel-Cox) test for (H). ***P < 0.001.

Building on the battery’s capability to effectively regulate TME and immunity, the in vivo antitumor effect was investigated using a 4T1 triple-negative breast cancer model, which is aggressive, poorly immunogenic, and metastasizes spontaneously to distant organs. 4T1-bearing female white mice were randomly divided into five groups (*n* = 5) and treated with Ctr, Zn, MnO_2_, UB, and DB, respectively. The implantation method of the battery is shown in figs. S32 and S33 (*54*). Over the next 14 days, the tumor volumes and body weights of the mice were recorded daily in detail (Fig. 5A). After 14 days of treatment, visual differences in the tumor structures were observed (Fig. 5B). The tumor in the control group exhibited a 21.5-fold increase in volume, implying the fast growth and malignancy of tumor cells. In comparison, all treatment groups showed the abilities in TGIs (Fig. 5, C to E). The Zn electrode and MnO_2_ electrode groups showed slight tumor inhibition with rates of 21.7 and 33.7%, respectively. The UB group demonstrated a moderate TGI rate of 60.4%. In sharp contrast, the DB group achieved superior antitumor efficacy, notably inhibiting tumor growth with a tumor inhibition rate of up to 99.6% (fig. S34). This is probably because the Zn//MnO_2_ battery showed stable output for 11 days, with an average discharge voltage of 0.9 V on day 0, 0.6 V on day 3, 0.3 V on day 7, and 0.1 V on day 11 (Fig. 5F) (*55*). This prolonged discharge could deliver a sustained discharge current and release of Zn and Mn ions, thereby exerting antitumor effects (Fig. 5, I and J). In addition, during the 14-day treatment, the average weights of mice in all groups gradually increased, indicating the good biosafety of this treatment strategy (Fig. 5G). Consistent with the TGI results, survival analysis from the Kaplan-Meier plots showed that the survival time of mice was remarkably prolonged in the DB group compared to other groups, which achieved a 100% survival ratio after 50 days of evaluation (Fig. 5H). All above indicates the excellent antitumor effect of the DB group.

**Fig. 6.**
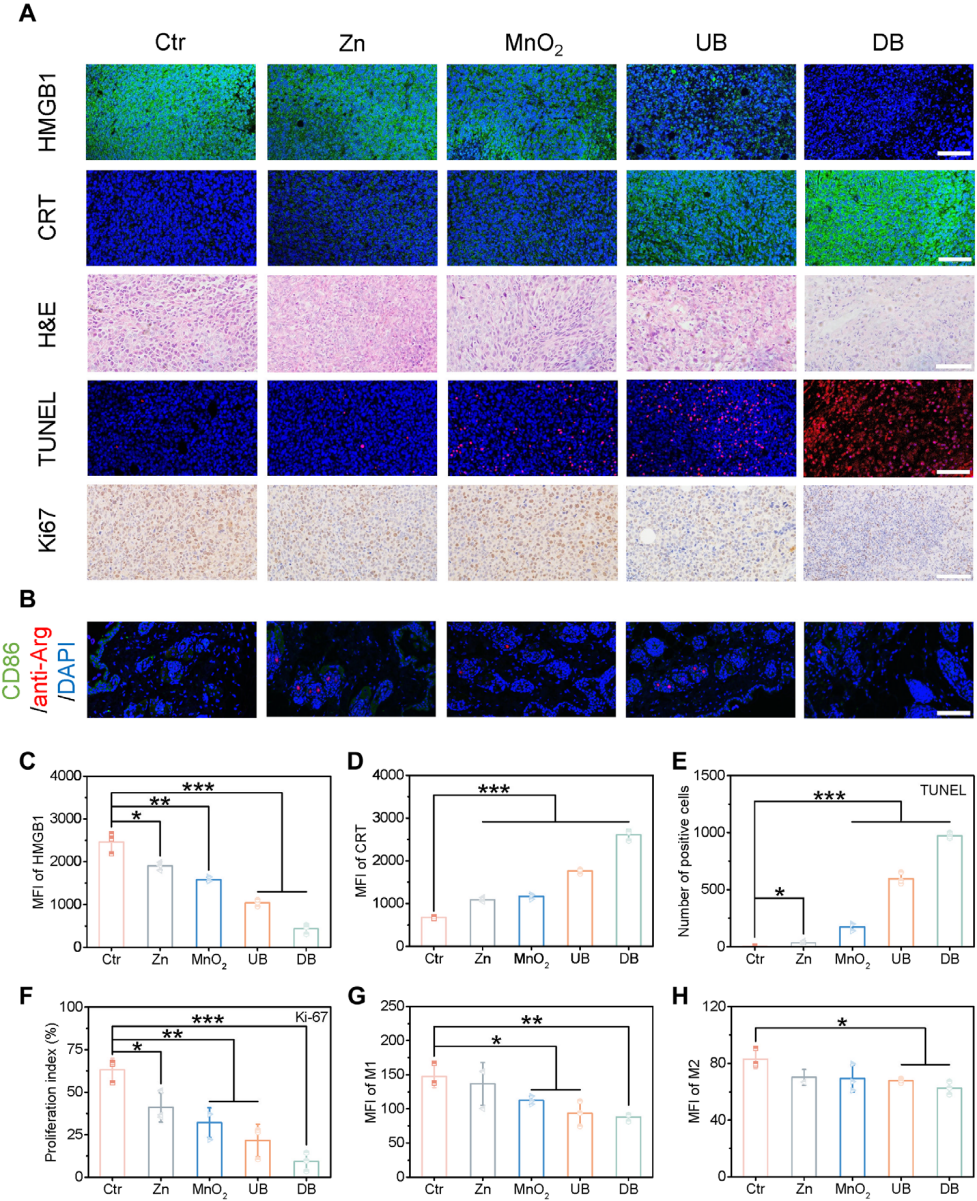
Pathological investigation treated with various groups. (**A**) Ex vivo HMGB1, CRT, H&E, TUNEL, and Ki67 staining of tumor tissues collected from the mice treated with various groups. (**B**) Immunofluorescence staining of the skin tissue around the implanted site. Fluorescence quantitative analysis of (**C**) HMGB1 and (**D**) CRT. (**E**) The quantification of TUNEL-positive cells. (**F**) The proliferation index of Ki67. Quantitative intensity of (**G**) M1- and (**H**) M2-type macrophage. Scale bars, 100 μm. All data are expressed as means ± SD (*n* = 3), and the differences were assessed by one-way ANOVA followed by Dunnett’s multiple comparisons tests. *P < 0.05, **P < 0.01, and ***P < 0.001.

To further investigate the antitumor efficacy, tumor tissues were preserved to validate the battery-mediated ICD effect (Fig. 6, A, C, and D, and figs. S35 to S39). Compared to the control group, the single-electrode groups exhibited the ICD effect, characterized by decreased HMGB1 fluorescence, increased CRT fluorescence, and reduced ATP content. The UB group demonstrated a stronger ICD effect due to the synergistic action of Zn and Mn ions. Notably, the DB group showed the highest release of DAMPs and exposure to CRT protein, which induced the strongest ICD effect and promoted cell death. Furthermore, as shown in hematoxylin and eosin (H&E) staining on the 14th day, the tumor cells in the UB and DB groups showed severe tissue necrosis and nuclear pyknosis across a large tumor area. In particular, the presence of cell nuclei was hardly observed in the DB group, while the cell morphology in the control group remained normal. Terminal deoxynucleotidyl transferase–mediated deoxyuridine triphosphate nick end labeling (TUNEL) staining showed that the tumor tissue slices treated with the DB group exhibited a strong red fluorescence signal, representing the occurrence of a large number of tumor cell death. In comparison, local red fluorescence was present in other groups (Fig. 6E and fig. S40). In addition, the staining and proliferation index of Ki67 further demonstrated that the DB group has the most obvious ability to inhibit tumor cell proliferation (Fig. 6F). All the results implied that the Zn//MnO_2_ battery effectively induced cell apoptosis and inhibited tumor growth. In addition, to further evaluate the biosafety of the Zn//MnO_2_ battery, we harvested the main organs and the skin tissue (around the material implanted site) of mice after various treatments for H&E staining and M1-type/M2-type macrophage staining (Fig. 6, B, G, and H, and figs. S41 and S42). Negligible damage or inflammation was observed, proving the excellent biocompatibility and biosafety of the Zn//MnO_2_ battery antitumor system.

### Evaluation of the antitumor immune response in vivo

**Fig. 7.**
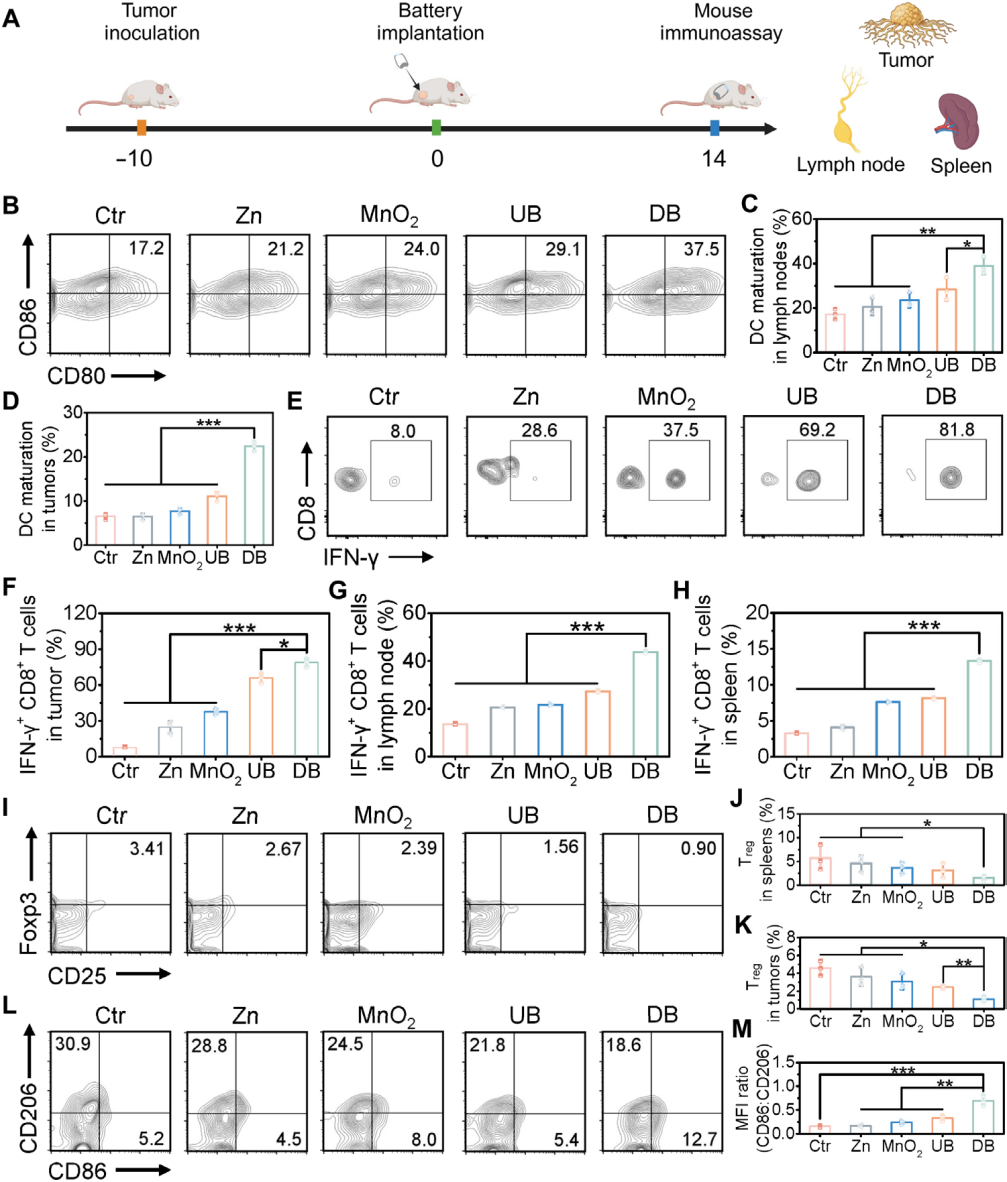
Battery-mediated immune activation by multiple mechanisms. (**A**) Schematic diagram of battery-mediated immunoassay. (**B**) FCM analysis and (**C**) quantification of mature DC in LNs. (**D**) Quantification of DC maturation in TDLNs. (**E**) Expression of IFN-γ^+^ in CD8^+^ T cells within tumor detected by flow cytometry. Statistical analysis of IFN-γ^+^ in CD8^+^ T cells within (**F**) tumor, (**G**) LNs, and (**H**) spleen. (**I**) FCM analysis and (**J**) quantification of T_regs_ in spleens. (**K**) Quantitative analysis of T_regs_ in tumors. (**L**) FCM analysis of the proinflammatory M1 phenotype macrophage and the protumor M2 phenotype macrophage in tumor tissues. (**M**) Quantitative analysis of the M1-type/M2-type macrophage ratio. All data are expressed as means ± SD (*n* = 3), and the differences were assessed by one-way ANOVA with Tukey’s posttest. *P < 0.05, **P < 0.01, and ***P < 0.001.

Inspired by the superior therapeutical efficacy of the Zn//MnO_2_ battery, we next explored the in vivo antitumor immune response in depth. The lymph nodes (LNs) and spleen are the most crucial immune tissue and organ, containing numerous lymphocytes which are the centers of the body’s immunity. In addition, tumor-draining lymph nodes (TDLNs) are the first sites where TAAs reach through lymphatic vessels, are taken up by DCs, and are presented to T cells for adaptive immune responses to eliminate tumor cells. Therefore, we collected the LNs, spleens, and tumors from mice to evaluate the percentage of immune-related indicators by the FCM (FACSCalibur, BD) (Fig. 7A). In vitro studies showed that the discharged Zn//MnO_2_ battery exhibited the capability to effectively activate DCs. Therefore, we first stained the single-cell suspensions from LNs with anti-CD11c antibody [phycoerythrin (PE) conjugated], anti-CD80 antibody (PE/cyanince 7 conjugated), and anti-CD86 antibody (FITC conjugated) to investigate DC maturation in vivo. Compared with the control group, the single-electrode groups only induced minor DC maturation, while the expression of the surface molecules CD80 and CD86 was markedly enhanced to 29.1% in the UB group (Fig. 7, B and C). Notably, mice treated with the DB group showed a much stronger ability in the promotion of DC maturation with CD80^+^CD86^+^ being increased to 37.5% due to the massive release of Mn^2+^ and Zn^2+^ and the effect of discharge current. Moreover, DB treatment also promoted more DC maturation in tumors (Fig. 7D and fig. S43). These results suggested that the DB group effectively induced DC maturation in vivo, which was beneficial to initiate the downstream immune response by modulating the proliferation of T cells.

T cells are major components of the adaptive immune response. For example, CD8^+^ T cells play an essential role in recognizing TAAs and mediating tumor cell killing, while CD4^+^ helper T cells participate in stimulating DC priming and activating CD8^+^ T cells. Therefore, we further evaluated the effect of Zn//MnO_2_ battery on local and systemic immune activation by counting CD4^+^ and CD8^+^ T cells in LNs, spleens, and tumors. Compared with other groups, DB treatment remarkably increased the proportion of CD4^+^/CD8^+^ in LNs and spleens to 4.2 and 7.6 (fig. S44, A, B, E, and F). A similar increase in CD4^+^ and CD8^+^ T cells was also observed in tumors, where the DB group enhanced them by 2.9- and 3.1-fold, respectively (fig. S44, C, D, G, and H). However, it is worth noting that the content of CD8^+^ T cells was decreased after being treated with different groups in LNs and spleens (fig. S44, A and B). This may be because, after 14-day treatment, the immune therapy has entered the effector phase. Activated CD8^+^ T cells in the LN and spleen infiltrated into the tumor site to exert their cytotoxic effects. Since tumor elimination mainly relies on cytotoxic T lymphocytes (CTLs), the proportion of IFN-γ^+^ in CD8^+^ T cells was assessed in the LNs, spleen, and tumor to further identify immune responses induced by the Zn//MnO_2_ battery (Fig. 7, E to H). The DB group showed the highest level of IFN-γ^+^ in CD8^+^ T cells with 81.8% in the tumor, compared to the control group (only 8.0%), the single-electrode groups (28.6 and 37.5%), and the UB group (69.2%). In the LNs and spleen, the control group showed a very small proportion of IFN-γ*^+^* in CD8^+^ T cells. In sharp contrast, a remarkably higher proportion was obtained in the DB group (fig. S45). These results demonstrated that a robust T cell immune response was triggered in the LN, spleen, and tumor.

In addition, to explore the activating effect of Zn//MnO_2_ battery in the early stage of the immune response, we also examined the content of CD4^+^ T cells, CD8^+^ T cells, and IFN-γ^+^ T cells in the LNs, spleen, and tumor after a 7-day treatment (figs. S46 and S47). Compared to the control group, the single-electrode (Zn and MnO_2_) and UB groups showed increased levels of CD8^+^ T cells (29.0, 30.1, and 30.2%) and IFN-γ^+^ T cells (2.57, 2.87, and 3.94%) in LNs. These results showed that day 7 may represent an early stage of immune activation, marked by initial CD8^+^ T cell infiltration and increased IFN-γ^+^ levels. The strongest immune activation observed in the DB group may be attributed to the metal ions and discharge current. In addition, similar trends were observed in the spleen and tumor tissues. These results suggest that the Zn//MnO_2_ battery effectively primed T cell maturation and expansion in vivo.

The immunosuppressive TME contributes to the escape of tumors from immunotherapy. Regulatory T cells (T_regs_) mediate immunosuppression by suppressing T effector cell function in the immune microenvironment, facilitating tumor development and progression. Massive tumor-associated macrophages (TAMs) are converted into the M2 type, which protects tumor cells from immune surveillance and promotes tumor development. To clarify whether the Zn//MnO_2_ battery could reverse the immunosuppressive TME, we analyzed the expression of T_reg_ in spleens and tumors and TAM in the tumor by flow cytometry (Fig. 7, I to K, and fig. S48). DB treatment remarkably reduced the percentage of T_regs_ in spleens and tumors to 0.90 and 0.88%, which was 3.41 and 2.75% of mice treated with the control group. The population of M1-type macrophages and M2-type macrophages in tumors was also investigated (Fig. 7, L and M). The DB group markedly increased the expression of the M1/M2 ratio to 4.4-, 4.2-, 2.9-, and 2.1-fold higher than after treatment with other groups, indicating that the relieved immunosuppression enhanced the killing effect of immune cells.

To sum up, the Zn//MnO_2_ battery induced potent antitumor immune responses, which were mediated through multiple mechanisms. On one hand, the battery effectively generated synergistic immune activation by promoting DC maturation, CD8 cytotoxic T cell secretion, and CD4 helper T cell secretion. On the other hand, it alleviated the immunosuppression in the TME by obviously reducing the ratio of T_regs_ and polarizing the M2 macrophages to the M1 macrophages, unleashing a cascade adaptive immune response. This created an immune-supportive TME enhancing the long-lasting response of immune cells and inhibiting tumor growth.

The foreign body response (FBR) was studied to verify whether the implantation itself may cause a notable immune response in the body. Therefore, we implanted electrochemically inert Pt and pure carbon cloth electrodes of the same size as the DB group in the 4T1 tumor–bearing mice. The levels of inflammatory cytokines in the serum were measured after different treatments (fig. S49). Compared to the control group, implantation showed no obvious changes in the levels of inflammatory cytokines after 7- and 14-day treatments, indicating that the implantation did not trigger a notable inflammatory response. Notably, the DB group induced the highest secretion level of inflammatory cytokines with the continuous generation of Zn, Mn, and discharge current, indicating that the Zn//MnO_2_ battery activated potent systemic immunity. In addition, it will be interesting to implant longer-lasting devices before tumor inoculation for letting the FBR pass, which would provide a clearer understanding of the inflammation state of the TME without any influence from surgical procedure and FBR.

**Fig. 8.**
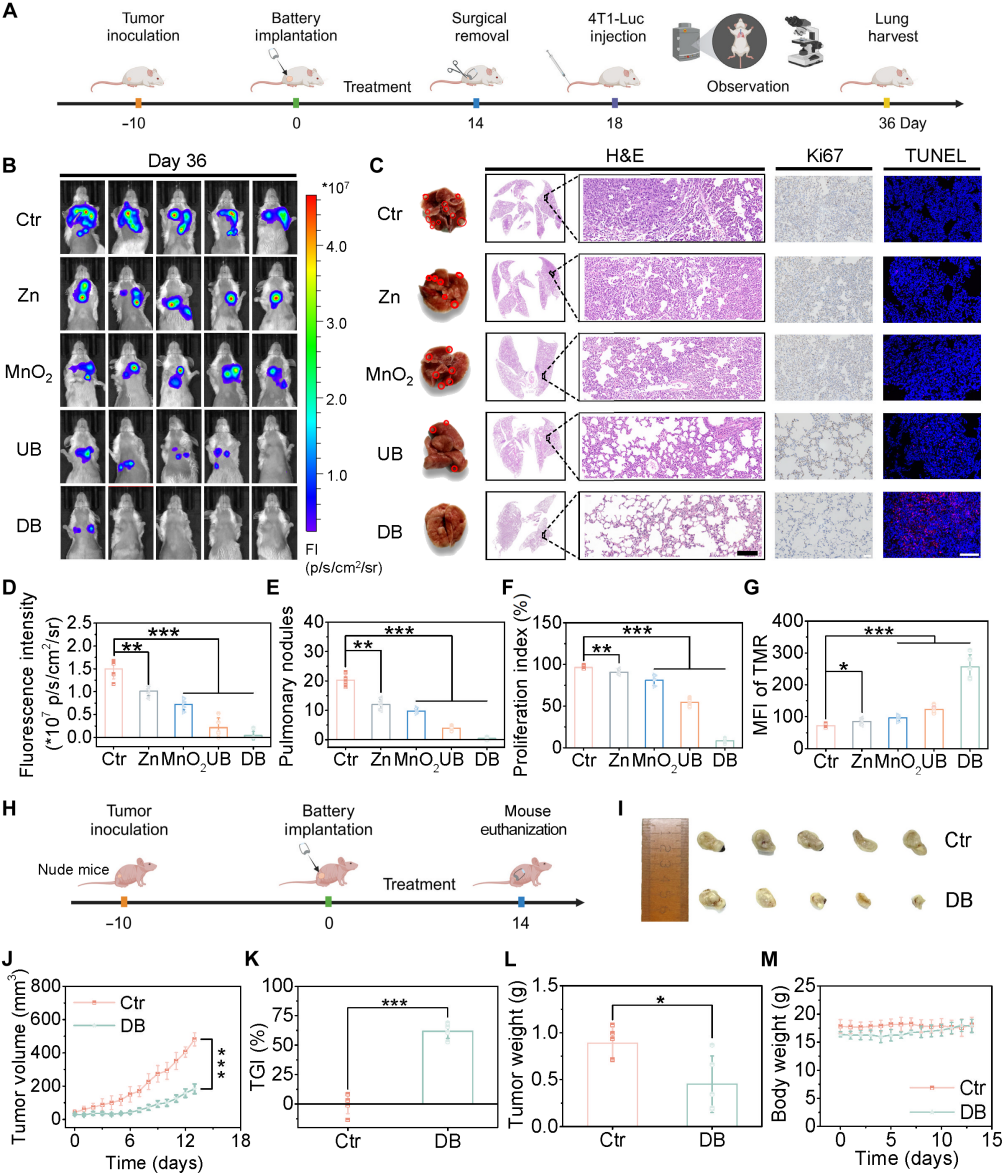
Antimetastatic and non-immune effect of battery in vivo. (**A**) Schematic diagram of battery-mediated therapy against lung metastasis. (**B**) The living bioluminescence imaging signals of mice and (**D**) quantification of luciferase signal on day 36. (**C**) Digital photographs, ex vivo H&E staining, Ki67 staining, and TUNEL staining of the representative lung on day 36. Quantification of (**E**) pulmonary nodules, (**F**) the proliferation index of Ki67, and (**G**) the TUNEL-positive cells on day 36. (**H**) Schematic diagram of battery-mediated non-immune treatment process. (**I**) Representative photos of tumor tissues. (**J**) Average tumor volume curves, (**K**) TGI rates, (**L**) tumor weight curves, and (**M**) body weight curves of mice after various treatments. FI, fluorescence intensity. Scale bars, 100 μm. All data are expressed as means ± SD (*n* = 5), and the differences were assessed by one-way ANOVA followed by Dunnett’s multiple comparisons tests for (D) to (G) and two-tailed Student’s *t* test for (J) to (L). *P < 0.05, **P < 0.01, and ***P < 0.001.

To evaluate the long-term in vivo immune memory effect of Zn//MnO_2_ battery, the tumor metastasis model was established by a second exposure to 4T1-Luc cells (Fig. 8A). First, to monitor the lung metastasis of tumor cells, in vivo bioluminescence imaging was performed on day 36 (Fig. 8, B and D). The result highlighted the impact of the DB group in inhibiting cancer cell metastasis, as evidenced by the almost complete absence of luminescence in the DB group compared to the high luminescence observed in the control group, which is attributed to the combined effect of discharge current and metal ions. The luminescence intensity also showed a reduction in the single-electrode and the UB groups, due to the small amount of metal ions released by chemical reactions (H^+^ and/or GSH). Furthermore, all mice were euthanized after imaging to dissect the lung tissues, and the lung nodules were labeled (Fig. 8C). An obvious difference between the control group and the DB group could be observed by bare eyes. The histopathological sections and quantitative analysis of lung metastatic nodules indicated that the DB group almost completely blocked the possibility of tumor lung metastasis (Fig. 8E). The proliferation of tumor cells in the lungs was determined using Ki67 staining (Fig. 8F). Compared to the highest proliferation in the control group, the experimental groups showed varying degrees of inhibition, with the DB group exhibiting the lowest cell proliferation signals. The same conclusion was further validated by TUNEL staining (Fig. 8G and fig. S50). All the above results indicate that the DB group is the most effective treatment for promoting immune memory and combating tumor metastasis in the lungs.

In addition, to demonstrate the immune-activating properties of the Zn//MnO_2_ battery, we constructed a nude mice model for cancer treatment (Fig. 8H). The control group showed rapid tumor growth due to the malignant proliferation of 4T1 cells, while the DB group exhibited an inhibition effect of 61.8% (Fig. 8, I to K, and fig. S51). The changes in tumor weights showed similar results (Fig. 8L). It is worth noting that when the battery-mediated immune activation effects were excluded in the nude mice, the therapy effect of the battery was remarkably decreased compared to the 99.6% inhibition rate in BALB/c mice (fig. S34). During the whole period of treatment, the body weights of mice showed no obvious decrease, which further proved the safety of the Zn//MnO_2_ battery (Fig. 8M).

## DISCUSSION

In summary, we introduce an implantable Zn//MnO_2_ battery that uses TME fluids as the electrolyte, enabling extended discharge time, increased discharge current, and sustained release of Zn and Mn ions for drug-free cancer immunotherapy. This local electrochemical immunotherapy strategy permits sustained delivery of metal ions within tumor cells by initiating battery discharge in TME. Compared to other therapeutic methods, this implantable battery could reduce systemic toxicity and maintain continuous discharge for 11 days with single implantation, thus improving patients’ compliance and alleviating issues associated with short retention time of conventional injectable nanoagonists. Battery discharge generates Zn^2+^ and Mn^2+^ to induce ICD effects and release DAMPs in tumor cells. On the other hand, Mn^2+^ facilitated the cascade activation of the cGAS-STING pathway, leading to increased secretion of IFN-I and proinflammatory cytokines. These two mechanisms collectively promoted the maturation of DCs and the infiltration of T lymphocytes, boosting the body’s immune response. In addition, the battery reversed the tumor immunosuppressive microenvironment by reducing the level of T_regs_ and increasing the M1/M2 macrophages ratio. Under the combined effects mentioned above, this battery achieved a TGI rate of 99.6% in the mouse breast cancer model. Other diseases involving immune-related physiology or pathology, such as infectious, inflammatory, and autoimmune diseases, will also benefit from this battery delivery system. Overall, this work demonstrates that a microenvironment-activated battery effectively delivers metal ions and activates immune responses, making it a promising therapeutic device.

## MATERIALS AND METHODS

### Materials

Zinc sulfate heptahydrate (ZnSO_4_·7H_2_O, ≥99.5%), manganese sulfate monohydrate (MnSO_4_·H_2_O, ≥99.0%), and potassium permanganate (KMnO_4_, ≥99.5%) were purchased from Shanghai Aladdin Biochemical Technology Co. Ltd. Sodium dihydrogen phosphate and disodium hydrogen phosphate were purchased from Sinopharm Chemical Reagent Co. Ltd. A Calcein-AM/PI double staining kit, an annexin V–FITC/PI apoptosis detection kit, a mitochondrial membrane potential assay kit (JC-1), Hoechst 33342, DCFH-DA, an enhanced ATP assay kit, and a prestained protein ladder were acquired from Dalian Meilun Biotechnology Co. Ltd. 2',7'-Bis-(2-Carboxyethyl)-5-(and-6)-Carboxyfluorescein (BCECF acetoxymethyl ester) was bought from Shanghai Maokang Biotechnology Co. Ltd. DTNB was supplied by Energy Chemical Reagent Co. Ltd. A mouse HMGB1 ELISA kit was obtained from Wuhan Fine Biotech Co. Ltd. Anti-CALR polyclonal antibody was purchased from Beijing Solarbio Science and Technology Co. Ltd. A mouse IL-6 ELISA kit, a mouse IFN-γ ELISA kit, a mouse IFN-β ELISA kit, a mouse TNF-α ELISA kit, a mouse CXCL10 ELISA kit, and an IRF-3 (phospho-Ser^396^) polyclonal antibody were acquired from Beijing 4A Biotech Co. Ltd. Phospho-TMEM173/STING (Ser^366^) antibody, phospho-TBK1 (Ser^172^) antibody, and TMEM173/STING antibody were bought from Beijing Affinity Biosciences. Anti–β-actin mouse monoclonal antibody (1C7), goat anti-mouse immunoglobulin G (IgG) [horseradish peroxidase (HRP)], goat anti-rabbit IgG (HRP), and a super-rapid protein quantification kit [bicinchoninic acid assay (BCA) assay] were supplied by Wuhan Abbkine Scientific Co. Ltd. PE-labeled CD11c, PE/Cyanine7-labeled CD80, FITC-labeled CD86, allophycocyanin (APC)–labeled F4/80, PE/Cyanine7-labeled CD206/MMR, CD86 polyclonal antibody, arginase-1 monoclonal antibody, peridinin-chlorophyll-protein complex (PerCP)/Cyanine5.5-labeled CD3, FITC-labeled CD4, PE-labeled CD8a, and APC-labeled Foxp3 were obtained from Elabscience Biotechnology Co. Ltd. 4′,6-Diamidino-2-phenylindole (DAPI) was purchased from Wuhan Servicebio Technology Co. Ltd. GSH was acquired from Shanghai Bide Pharmatech Co. Ltd. All reagents and materials were commercially available without further purification.

### Characterization

X-ray diffraction (XRD) patterns were measured by a D/max2500 PC diffractometer (Cu Kα radiation, 5° to 80°). XPS patterns were captured by ECALAB 250 with Al anode (Thermo Fisher Scientific). Fluorescence images were recorded using a two-photon CLSM (Nikon A1RMP). The cytotoxicity, GSH content, and HMGB1 content were collected on a microplate reader (Bio-Tek Instruments Inc.). The ATP content was captured using a multifunctional enzyme marker. FCM analysis was obtained by CytoFlex (Beckman) and (BD FACSCalibur). The changes in the pH value of the cell supernatant were performed by a PH meter (PHS-3C). The contents of Mn and Zn were measured using an inductively coupled plasma-optical emission spectrometer (ICP-OES; Agilent 725). A battery testing system (Neware BTS 4000) was used to capture the electrochemical performance, and an electrochemical workstation (CHI660E) was used to investigate the CV tests. Bioluminescence images were recorded using a vivo imaging system (PerkinElmer, IVIS Lumina Series III).

### Preparation of α-MnO_2_ electrode

A total of 2.5 g of MnSO_4_·H_2_O and 2 ml of H_2_SO_4_ (0.5 M) were mixed with 80 ml of ultrapure water under stirring constantly for 5 min. Subsequently, 1.6 g of KMnO_4_ was added to the aforementioned solution. Following dissolving, the mixed solution was transferred into a 100-ml Teflon-lined stainless steel autoclave and maintained at 120°C for 24 hours. After the temperature of the solution had reached room temperature, the brown substance was collected by filtration, cleansed with ultrapure water and ethanol, and then dried at 80°C for 12 hours.

The cathode was fabricated by mixing the active materials of α-MnO_2_, polyvinylidene difluoride (average molecular weight, ~1,000,000), and acetylene black (C) in a weight ratio of 7:2:1 onto a clean carbon cloth that had been activated and then dried at 60°C under a vacuum condition for 12 hours. The areal loading density is around 2 to 4 mg cm^−2^.

### Electrochemical test

#### CV of α-MnO_2_

The electrochemical properties of α-MnO_2_ were evaluated using a three-electrode system. The system consisted of a platinum wire as the counter electrode, an Ag/AgCl electrode as the reference electrode, an α-MnO_2_ electrode as the working electrode, and 2 M ZnSO_4_ and 0.5 M MnSO_4_ solution as the electrolytes.

#### CV of Zn//MnO_2_ battery

The electrochemical characterization of Zn//MnO_2_ battery was tested in 2 M ZnSO_4_ and 0.5 M MnSO_4_ electrolytes. Equation 8 was used to determine the relative contributions of diffusion-controlled (k_1_v^1/2^) and capacitive (k_2_v) processes

$$i=k_{1}v+k_{2}v^{\frac{1}{2}}$$ (8)

where k_1_ and k_2_ are adjusted parameters at specific scan rates.

#### CV of MnSO_4_ or GSH

The electrochemical property of MnSO_4_ or GSH was evaluated using a three-electrode system. The system consisted of a platinum wire as the counter electrode, an Ag/AgCl electrode as the reference electrode, a glassy carbon electrode as the working electrode, and 0.5 M MnSO_4_ solution or 6.8 PB and 100 μM GSH solution as the electrolytes.

#### LSV of MnO_2_

MnO_2_ particles (4 mg/ml) were dispersed in a solution with water:ethanol:Nafion ratio of 490:490:20. The mixture was ultrasonicated until no visible particles remained. Then, 20 μl of the sample solution was evenly pipetted onto the rotating disk electrode and dried with an infrared lamp. Before starting the measurement, oxygen was bubbled through the PB electrolyte (pH = 6.8) for half an hour, and the oxygen supply was maintained throughout the experiment. The rotation speed was set to 1600 rpm, and an electrochemical workstation (CHI660e) was used to record the LSV curves.

#### Discharging and charging performances of the Zn//MnO_2_ battery

The Zn//MnO_2_ battery was tested for galvanostatic discharge and charge on a Neware battery tester. Galvanostatic discharge was performed to a voltage of 0.8 V and then galvanostatic charge to 1.9 V. First, 2 M ZnSO_4_ and 0.5 M MnSO_4_ were chosen as the electrolytes. The current densities were 0.1, 0.2, 0.5, 1, and 2 A g^−1^. Each current density was cycled 10 times. Second, the performances were tested in 7.4 PB or 6.8 PB and 100 μM GSH electrolytes at a current density of 25 mA g^−1^.

The battery was tested for constant resistance discharge on an electrochemical workstation. The resistance values of the fixed resistor are 50, 100, and 150 kilohm. α-MnO_2_ was used as the cathode, Zn as the anode, and 7.4 PB or 6.8 PB and 100 μM GSH or cell culture medium containing 4T1 cells were used as the electrolytes. For the in vivo test, copper wires were connected to the battery’s two electrodes using silver paste. To enhance the stability of the connection, the junction was sealed with polydimethylsiloxane. The discharging voltage was measured daily for 1 hour by the electrochemical workstation in eight individual mice, with a representative curve given as the result.

#### EPR test of ·OH

The battery underwent constant resistance discharge with a resistor of 100 kilohm. The electrolyte was PB solution (pH = 6.8). Liquid samples were collected at 12 hours for EPR testing. The capture agent was 5,5-dimethyl-1-pyrroline N-oxide (DMPO).

#### XRD and XPS tests of the Zn//MnO_2_ battery

At different time points of galvanostatic charging and discharging (25 mA g^−1^) of the battery, MnO_2_ cathodes were taken for XRD and XPS testing. Initial electrolytes were 7.4 PB or 6.8 PB and 100 μM GSH.

#### GSH content test

One milligram of MnO_2_ was mixed with 50 ml of 6.8 PB and 1 mM GSH solution. At different time points, 100 μl of the supernatant was collected at 4°C. Then, GSH concentrations were detected by Ellman’s assay.

### Monitoring Zn and Mn ion content

#### In vitro test

After discharging, the electrolyte was collected. The concentration of Zn and Mn ions was determined by ICP-OES. In addition, levels in the supernatants and cell lysates were also quantified.

#### In vivo test

Upon completion of treatment, the hearts, livers, spleens, lungs, kidneys, and tumors of mice were harvested. After the preparation of single-cell suspensions and the implementation of membrane-breaking treatments, ICP-OES was used to detect the concentrations of Zn and Mn in the cell lysates.

### Cell lines

4T1, B16-F10, HepG2, Hepa 1-6, and HeLa cell lines were cultured in the Dulbecco’s modified Eagle’s medium (DMEM) medium containing fetal bovine serum (FBS; 10%) and streptomycin/penicillin (PS; 1%). BMDCs were obtained from bone marrow cells flushed from the femurs and tibias of 6-week-old female Balb/c mice. The harvested cells were cultured in RPMI 1640 medium containing 10% FBS, 1% PS, granulocyte-macrophage colony-stimulating factor (20 ng ml^−1^), and IL-4 (10 ng ml^−1^) for 6 days. Similarly, BMDMs were collected and cultured in the complete DMEM medium containing macrophage colony-stimulating factor (10 ng ml^−1^) for a duration of 1 week. All cells were cultured at 37°C with 5% CO_2_ in a conventional cell incubator.

### Battery-mediated microenvironment regulation

#### ΔΨ*m test*

4T1 cells (5 × 10^5^ cells per well) were cultured in six-well plates and treated with 1× PBS, Zn, MnO_2_, UB, and DB for 12 hours. Then, the cells were collected and stained with a JC-1 probe. All results were acquired by FCM. In addition, the ΔΨm of JC-1–stained 4T1 cells were observed using CLSM.

#### Cell permeation pH study

4T1 cells were incubated with the aforementioned five treatments (1× PBS, Zn, MnO_2_, UB, and DB) and the IEs. Subsequently, the intracellular pH and cell nucleus were labeled with BCECF AM and Hoechst 33342 fluorescent probes, respectively. All the images were obtained by CLSM. Furthermore, the cell supernatants were collected at different time intervals to detect the pH values using a pH detector.

#### Intracellular ROS assessment

To evaluate the intracellular ROS production, the DCFH-DA probe was coincubated with 4T1 cells treated with various groups (1× PBS, Zn, MnO_2_, UB, DB, and IE). The fluorescent emissions of DCF were detected using CLSM. Furthermore, FCM was used to quantitatively evaluate the intracellular ROS level.

#### Intracellular GSH level analysis

4T1 cells (5 × 10^5^ cells per well) were grown in six-well plates and coincubated with 1× PBS, Zn, MnO_2_, UB, DB, and IE. Following lysis through 3 cycles of freezing and thawing, the treated cells were centrifuged, and the supernatants were harvested. Subsequently, GSH concentrations were detected by Ellman’s assay.

### Cytotoxicity and cell apoptosis

#### MTT assay

4T1 cells, B16-F10 cells, HeLa cells, HepG2 cells, Hepa 1-6 cells, 3T3 cells, and HL-1 cells were seeded in 24-well plates at a density of 1 × 10^5^ cells per well and cultured overnight for cell attachment. Subsequently, Zn and MnO_2_ were added to the plate vertically in the form of single electrodes and UB and Zn//MnO_2_ batteries connected with different resistances (50, 100, and 150 kilohm), respectively. After coincubation for 6 hours, the relative cell viabilities were quantified.

#### Live/Dead stain and apoptosis assay

The dead and live results of 4T1 cells treated with various groups for 12 hours were stained by calcein-AM/PI staining using two-photon CLSM. In addition, the apoptosis levels after a duration of 12 hours were determined by an annexin V–FITC/PI kit and then evaluated by FCM.

#### Cell scratch test

4T1 cells were seeded in 10-mm confocal dishes. After the cell proliferation reached 100%, a blank area was created artificially. Subsequently, the cells were incubated with various treatments (1× PBS, Zn, MnO_2_, UB, and DB), and the migration of cells was observed at different time points using CLSM.

### Immune activation in vitro

#### Determination of ICD markers

4T1 cells were seeded in six-well plates or confocal dishes at a density of 5 × 10^5^ cells per well and incubated with various treatments (1× PBS, Zn, MnO_2_, UB, and DB) for 12 hours. The treated cells were collected and lysed to assess the intracellular levels of HMGB1 and ATP using the corresponding ELISA kit. To visualize the surface expression of CRT/HMGB1, the treated cells in confocal dishes were incubated with anti-CALR polyclonal antibody (FITC conjugated)/anti-HMGB1 polyclonal antibody (FITC conjugated) and Hoechst 33342. Then, CRT/HMGB1 expression was observed by CLSM. In addition, the expression of HMGB1 was also tested by Western blot.

#### ICD-induced BMDC maturation

Six-well Transwell systems with 0.4-μm porous membrane were used to detect the maturation of BMDCs. Specifically, 4T1 cells were seeded in the upper wells individually and treated with different groups (1× PBS, Zn, MnO_2_, UB, and DB). Then, the treated 4T1 cells were coincubated with BMDCs in the bottom wells. FCM was used to gather fluorescence signals of CD80^+^CD86^+^ (PE/Cyanine7 and FITC conjugated) in CD11c^+^ (PE conjugated).

#### Battery-stimulated BMDC maturations

BMDCs were cultured on six-well plates and coincubated with various treatments (1× PBS, Zn, MnO_2_, UB, and DB) directly. After staining with anti-CD11c (PE conjugated), anti-CD80 (PE/Cyanine7 conjugated), and anti-CD86 (FITC conjugated) antibodies, the treated BMDCs were analyzed by FCM. In addition, corresponding ELISA kits were used to quantify the secretion level of cytokines in the supernatants of 24-well plates cell cultures, including IL-6, IFN-β, IFN-γ, CXCL10, and TNF-α, to evaluate the ICD and battery activation of BMDCs.

#### Activation of downstream signaling pathways of STING

BMDCs were seeded in six-well plates prior and coincubated with various groups (1× PBS, Zn, MnO_2_, UB, and DB) or treated 4T1 cells. Proteins were extracted from cells, and the concentrations were determined using a BCA protein assay kit. The expression levels of STING, P-STING, TBK1, P-TBK1, IRF-3, and P-IRF-3 were assessed via Western blot to evaluate the activation of the STING pathway.

#### Battery-induced BMDM polarization

BMDMs were seeded in six-well plates and coincubated with 1× PBS, Zn, MnO_2_, UB, and DB directly. Following staining with anti-F4/80 (APC conjugated), anti-CD86 (FITC conjugated), and anti-CD206 (PE/Cyanine7 conjugated) antibodies, the polarization of M2 macrophages into M1 macrophages was detected by FCM.

### Animal

Balb/c mice (female, 6 weeks) were purchased from Changchun Yisi Experimental Animal Technology Co. Ltd., and BALB/c-nu mice (female, 6 weeks) were obtained from Liaoning Changsheng Biotechnology Co. Ltd. All animal procedures were performed following the Guidelines for Care and Use of Laboratory Animals, and all procedures were approved by the Animal Care and Use Committee of Jilin University (SY202501006).

### In vivo antitumor efficacy of battery

4T1 cells (1 × 10^6^ per mouse) in PBS (100 μl) were inoculated into the right flank of mice to establish the xenograft tumor models. When the tumor volume reached 50 to 60 mm^3^, 4T1 tumor–bearing mice were randomly divided and implanted with 1× PBS, Zn, MnO_2_, UB, DB, and IEs. The tumor volume and weight of mice were recorded every day using a vernier caliper and a balance. The subsequent formula was used to calculate the tumor volume

$$V=\frac{1}{2}ab^{2}$$ (9)

where *a* and *b* represent the length and width of the tumor, respectively. TGI was computed using the following equation

$$\text{TGI}\;\left(\%\right)=\left(1-\frac{{\text{RTV}}_{\text{treatment}}}{{\text{RTV}}_{\text{control}}}\right)\times 100$$ (10)

where RTV_treatment_ denotes the relative tumor volume of the groups after treatment, and RTV_control_ signifies the 1× PBS group after treatment. At the end of treatments (14 days), parts of mice were euthanized, and the hearts, livers, spleens, lungs, kidneys, tumors, LNs, and epidermis were harvested for the subsequent histological analysis and immunological effect studies. The remaining mice in each group continued to measure tumor volume to determine the survival duration.

### In vivo antitumor metastasis efficacy of battery

First, 4T1 cells (1 × 10^6^ per mouse) were inoculated into the right flank of BALB/c mice. When tumors reached 50 to 60 mm^3^, treatments with 1× PBS, Zn, MnO_2_, UB, and DB were administered. On day 14 posttreatment, the remaining tumors were surgically excised. Subsequently, mice were injected with 2 × 10^5^ 4T1-Luc cells via the tail vein to develop lung metastasis. Bioluminescence imaging of lung tumors was conducted on day 36 using the in vivo imaging system. Lungs were then excised for H&E, TUNEL, and Ki-67 staining to evaluate the effectiveness of the Zn//MnO_2_ battery in inhibiting lung metastasis.

### Staining analysis of the organs and tissues

#### Histology examination

The hearts, livers, spleens, lungs, kidneys, and tumor tissues were washed and placed in 4% paraformaldehyde solution overnight. All samples were sectioned into 5-μm-thick slices and stained with H&E for histological analysis. In addition, the tumor tissues were also stained by TUNEL and Ki-67. The stained samples were imaged on a confocal fluorescence microscope and a white light microscope.

#### Inflammation staining analysis

The epidermal tissues were rinsed and immersed in 4% paraformaldehyde solution overnight and cut into 5-μm-thick slices. After staining with CD86 polyclonal antibody (M1 type), arginase-1 monoclonal antibody (M2 type), and DAPI (nucleus), the samples were observed using a confocal fluorescence microscope.

#### HMGB1 and CRT analysis

Tumor tissues were washed and fixed in 4% paraformaldehyde overnight. All samples were sectioned into 5-μm-thick slices and stained with anti-HMGB1 polyclonal antibody (FITC conjugated) or anti-CRT polyclonal antibody (FITC conjugated). In addition, the nucleus was stained with DAPI. Imaging was performed on a confocal fluorescence microscope.

### Immunoassay *in vivo*

#### T lymphocyte immune activation in vivo

To assess the effect of the immune response of T lymphocytes, the spleens, tumors, and LNs of immunized mice were collected and processed into single-cell suspensions. After staining with anti-CD3 (PerCP/Cyanine5.5 conjugated), anti-CD4 (FITC conjugated), and anti-CD8 (PE conjugated) antibodies, the percentage of T lymphocytes was detected by FCM.

#### CTL activation in vivo

Cells were stained with anti-CD3 (PerCP/Cyanine5.5 conjugated), anti–IFN-γ (FITC conjugated), and anti-CD8 (PE conjugated) antibodies, and the percentage of CTLs was detected by FCM.

#### DC maturation in vivo

DC maturation in tumors and LNs can trigger specific T cell immune responses. Anti-CD11c, anti-CD80, and anti-CD86 antibodies were used to identify the expression of CD11c^+^CD80^+^CD86^+^ using FCM.

#### Macrophage polarization in vivo

M2-type macrophages promote tumor growth, while M1-type macrophages exert antitumor immunity. The tumor tissues were homogenized into single-cell suspensions and coincubated with anti-F4/80 (APC conjugated), anti-CD86 (FITC conjugated), and anti-CD206 (PE/Cyanine7 conjugated) antibodies. FCM was used to ascertain the ratio of M1/M2 (CD86:CD206).

#### T_reg_ analysis in vivo

Single-cell suspensions of tumors and spleens were stained with anti-CD4 (FITC conjugated), anti-CD25 (PE conjugated), and anti-Foxp3 (APC conjugated) antibodies to evaluate the mitigating effect of battery on the immunosuppressive TME of mice using an FCM.

#### Detection of inflammatory cytokines in serum

After treatment, the mice were euthanized, and blood was collected from the orbital sinus into 2-ml centrifuge tubes. The samples were kept at 4°C for at least 2 hours before centrifugation at 3000 rpm for 20 min. The supernatants were collected, and the centrifugation step was repeated once. The final supernatants were aliquoted and stored at −80°C. During testing, the serum samples were diluted 10-fold, and inflammatory cytokines (IL-6, TNF-α, IFN-β, IFN-γ, and CXCL10) were detected using ELISA kits.

## Supplementary Materials

### This PDF file includes:

Figs. S1 to S51 Tables S1 and S2 References

## References

[1] Y. Chao, Z. Liu, Biomaterials tools to modulate the tumour microenvironment in immunotherapy. Nat. Rev. Bioeng. 1, 125–138 (2023).

[2] Z. Chen, C. Meng, J. Mai, Y. Liu, H. Li, H. Shen, An mRNA vaccine elicits STING-dependent antitumor immune responses. Acta Pharm. Sin. B 13, 1274–1286 (2023). 36970194 10.1016/j.apsb.2022.11.013 PMC10031366

[3] X. Xue, H. Qu, Y. Li, Stimuli-responsive crosslinked nanomedicine for cancer treatment. Exp. Dermatol. 2, 20210134 (2022). 10.1002/EXP.20210134 PMC10190936 37324805

[4] S. K. Alsaiari, S. S. Qutub, S. Sun, W. Baslyman, M. Aldehaiman, M. Alyami, A. Almalik, R. Halwani, J. Merzaban, Z. Mao, N. M. Khashab, Sustained and targeted delivery of checkpoint inhibitors by metal-organic frameworks for cancer immunotherapy. Sci. Adv. 7, eabe7174 (2021). 33523955 10.1126/sciadv.abe7174 PMC10964966

[5] Z. Deng, M. Xi, C. Zhang, X. Wu, Q. Li, C. Wang, H. Fang, G. Sun, Y. Zhang, G. Yang, Z. Liu, Biomineralized MnO_2_ nanoplatforms mediated delivery of immune checkpoint inhibitors with STING pathway activation to potentiate cancer radio-immunotherapy. ACS Nano 17, 4495–4506 (2023). 36848115 10.1021/acsnano.2c10352

[6] C. Zhang, J. Huang, Z. Zeng, S. He, P. Cheng, J. Li, K. Pu, Catalytical nano-immunocomplexes for remote-controlled sono-metabolic checkpoint trimodal cancer therapy. Nat. Commun. 13, 3468 (2022). 35710545 10.1038/s41467-022-31044-6 PMC9203767

[7] B. Choi, H. Choi, H. Kim, A. Choi, S.-W. Kwon, S. K. Mouli, R. J. Lewandowski, D.-H. Kim, Z-domain protein nano-bio interfaced MRI visible anti-program death ligand-1 nanoconjugates for enhanced local immune checkpoint inhibitor immunotherapy. Nano Today 45, 101552 (2022).

[8] X. Xu, Z. Zhang, J. Du, Y. Xue, X. Chen, J. Zhang, X. Yang, D. Chang, J. Xie, S. Ju, Recruiting T-cells toward the brain for enhanced glioblastoma immunotherapeutic efficacy by co-delivery of cytokines and immune checkpoint antibodies with macrophage-membrane-camouflaged nanovesicles. Adv. Mater. 35, e2209785 (2023). 37101060 10.1002/adma.202209785

[9] Y. Agarwal, L. E. Milling, J. Y. H. Chang, L. Santollani, A. Sheen, E. A. Lutz, A. Tabet, J. Stinson, K. Ni, K. A. Rodrigues, T. J. Moyer, M. B. Melo, D. J. Irvine, K. D. Wittrup, Intratumourally injected alum-tethered cytokines elicit potent and safer local and systemic anticancer immunity. Nat. Biomed. Eng. 6, 129–143 (2022). 35013574 10.1038/s41551-021-00831-9 PMC9681025

[10] A. M. Nash, M. I. Jarvis, S. Aghlara-Fotovat, S. Mukherjee, A. Hernandez, A. D. Hecht, P. D. Rios, S. Ghani, I. Joshi, D. Isa, Y. Cui, S. Nouraein, J. Z. Lee, C. Xu, D. Y. Zhang, R. A. Sheth, W. Peng, J. Oberholzer, O. A. Igoshin, A. A. Jazaeri, O. Veiseh, Clinically translatable cytokine delivery platform for eradication of intraperitoneal tumors. Sci. Adv. 8, eabm1032 (2022). 35235346 10.1126/sciadv.abm1032 PMC8890714

[11] S. Liang, J. Yao, D. Liu, L. Rao, X. Chen, Z. Wang, Harnessing nanomaterials for cancer sonodynamic immunotherapy. Adv. Mater. 35, e2211130 (2023). 36881527 10.1002/adma.202211130

[12] M. Zhou, S. Liang, D. Liu, K. Ma, Y. Peng, Z. Wang, Engineered nanoprobes for immune activation monitoring. ACS Nano 16, 19940–19958 (2022). 36454191 10.1021/acsnano.2c09743

[13] G. Ji, L. Ma, H. Yao, S. Ma, X. Si, Y. Wang, X. Bao, L. Ma, F. Chen, C. Ma, L. Huang, X. Fang, W. Song, Precise delivery of obeticholic acid via nanoapproach for triggering natural killer T cell-mediated liver cancer immunotherapy. Acta Pharm. Sin. B 10, 2171–2182 (2020). 33304784 10.1016/j.apsb.2020.09.004 PMC7715527

[14] C. Ding, Z. Song, A. Shen, T. Chen, A. Zhang, Small molecules targeting the innate immune cGAS–STING–TBK1 signaling pathway. Acta Pharm. Sin. B 10, 2272–2298 (2020). 33354501 10.1016/j.apsb.2020.03.001 PMC7745059

[15] A. Decout, J. D. Katz, S. Venkatraman, A. Ablasser, The cGAS–STING pathway as a therapeutic target in inflammatory diseases. Nat. Rev. Immunol. 21, 548–569 (2021). 33833439 10.1038/s41577-021-00524-z PMC8029610

[16] J. Kwon, S. F. Bakhoum, The cytosolic DNA-sensing cGAS–STING pathway in cancer. Cancer Discov. 10, 26–39 (2020). 31852718 10.1158/2159-8290.CD-19-0761 PMC7151642

[17] M. Jiang, P. Chen, L. Wang, W. Li, B. Chen, Y. Liu, H. Wang, S. Zhao, L. Ye, Y. He, C. Zhou, cGAS-STING, an important pathway in cancer immunotherapy. J. Hematol. Oncol. 13, 81 (2020). 32571374 10.1186/s13045-020-00916-z PMC7310007

[18] X. Kong, H. Zuo, H.-D. Huang, Q. Zhang, J. Chen, C. He, Y. Hu, STING as an emerging therapeutic target for drug discovery: Perspectives from the global patent landscape. J. Adv. Res. 44, 119–133 (2023). 35636721 10.1016/j.jare.2022.05.006 PMC9936525

[19] K.-P. Hopfner, V. Hornung, Molecular mechanisms and cellular functions of cGAS–STING signalling. Nat. Rev. Mol. Cell Biol. 21, 501–521 (2020). 32424334 10.1038/s41580-020-0244-x

[20] L. Deng, H. Liang, M. Xu, X. Yang, B. Burnette, A. Arina, X.-D. Li, H. Mauceri, M. Beckett, T. Darga, X. Huang, T. F. Gajewski, Z. J. Chen, Y.-X. Fu, R. R. Weichselbaum, STING-dependent cytosolic DNA sensing promotes radiation-induced type I interferon-dependent antitumor immunity in immunogenic tumors. Immunity 41, 843–852 (2014). 25517616 10.1016/j.immuni.2014.10.019 PMC5155593

[21] R. Zhang, C. Wang, Y. Guan, X. Wei, M. Sha, M. Yi, M. Jing, M. Lv, W. Guo, J. Xu, Y. Wan, X.-M. Jia, Z. Jiang, Manganese salts function as potent adjuvants. Cell. Mol. Immunol. 18, 1222–1234 (2021). 33767434 10.1038/s41423-021-00669-w PMC8093200

[22] X. Sun, Y. Zhang, J. Li, K. S. Park, K. Han, X. Zhou, Y. Xu, J. Nam, J. Xu, X. Shi, L. Wei, Y. L. Lei, J. J. Moon, Amplifying STING activation by cyclic dinucleotide–manganese particles for local and systemic cancer metalloimmunotherapy. Nat. Nanotechnol. 16, 1260–1270 (2021). 34594005 10.1038/s41565-021-00962-9 PMC8595610

[23] C. Wang, Y. Guan, M. Lv, R. Zhang, Z. Guo, X. Wei, X. Du, J. Yang, T. Li, Y. Wan, X. Su, X. Huang, Z. Jiang, Manganese increases the sensitivity of the cGAS-STING pathway for double-stranded DNA and is required for the host defense against DNA viruses. Immunity 48, 675–687.e7 (2018). 29653696 10.1016/j.immuni.2018.03.017

[24] M. Lv, M. Chen, R. Zhang, W. Zhang, C. Wang, Y. Zhang, X. Wei, Y. Guan, J. Liu, K. Feng, M. Jing, X. Wang, Y.-C. Liu, Q. Mei, W. Han, Z. Jiang, Manganese is critical for antitumor immune responses via cGAS-STING and improves the efficacy of clinical immunotherapy. Cell Res. 30, 966–979 (2020). 32839553 10.1038/s41422-020-00395-4 PMC7785004

[25] J. Li, H. Ren, Q. Qiu, X. Yang, J. Zhang, C. Zhang, B. Sun, J. F. Lovell, Y. Zhang, Manganese coordination micelles that activate stimulator of interferon genes and capture in situ tumor antigens for cancer metalloimmunotherapy. ACS Nano 16, 16909–16923 (2022). 36200692 10.1021/acsnano.2c06926

[26] N. Fan, K. Chen, R. Zhu, Z. Zhang, H. Huang, S. Qin, Q. Zheng, Z. He, X. He, W. Xiao, Y. Zhang, Y. Gu, C. Zhao, Y. Liu, X. Jiang, S. Li, Y. Wei, X. Song, Manganese-coordinated mRNA vaccines with enhanced mRNA expression and immunogenicity induce robust immune responses against SARS-CoV-2 variants. Sci. Adv. 8, eabq3500 (2022). 36563159 10.1126/sciadv.abq3500 PMC9788765

[27] X. Sun, X. Zhou, X. Shi, O. A. Abed, X. An, Y. L. Lei, J. J. Moon, Strategies for the development of metalloimmunotherapies. Nat. Biomed. Eng. 8, 1073–1091 (2024). 38914800 10.1038/s41551-024-01221-7 PMC11410547

[28] B. Ding, P. Zheng, F. Jiang, Y. Zhao, M. Wang, M. Chang, P. a. Ma, J. Lin, MnO_x_ nanospikes as nanoadjuvants and immunogenic cell death drugs with enhanced antitumor immunity and antimetastatic effect. Angew. Chem. Int. Ed. Engl. 59, 16381–16384 (2020). 32484598 10.1002/anie.202005111

[29] Y. Wang, Y. Li, Z. Zhang, L. Wang, D. Wang, B. Z. Tang, Triple-jump photodynamic theranostics: MnO_2_ combined upconversion nanoplatforms involving a type-I photosensitizer with aggregation-induced emission characteristics for potent cancer treatment. Adv. Mater. 33, e2103748 (2021). 34423484 10.1002/adma.202103748

[30] J. Ou, H. Tian, J. Wu, J. Gao, J. Jiang, K. Liu, S. Wang, F. Wang, F. Tong, Y. Ye, L. Liu, B. Chen, X. Ma, X. Chen, F. Peng, Y. Tu, MnO_2_-based nanomotors with active fenton-like Mn^2+^ delivery for enhanced chemodynamic therapy. ACS Appl. Mater. Interfaces 13, 38050–38060 (2021). 34369138 10.1021/acsami.1c08926

[31] S. Chattopadhyay, Y.-H. Liu, Z.-S. Fang, C.-L. Lin, J.-C. Lin, B.-Y. Yao, C.-M. J. Hu, Synthetic immunogenic cell death mediated by intracellular delivery of STING agonist nanoshells enhances anticancer chemo-immunotherapy. Nano Lett. 20, 2246–2256 (2020). 32160474 10.1021/acs.nanolett.9b04094

[32] S. Ma, W. Song, Y. Xu, X. Si, S. Lv, Y. Zhang, Z. Tang, X. Chen, Rationally designed polymer conjugate for tumor-specific amplification of oxidative stress and boosting antitumor immunity. Nano Lett. 20, 2514–2521 (2020). 32109068 10.1021/acs.nanolett.9b05265

[33] M. L. Zhou, S. Liang, D. Liu, K. S. Ma, K. Q. Yun, J. J. Yao, Y. X. Peng, L. N. Hai, Q. Zhang, Z. H. Wang, Manganese-enriched zinc peroxide functional nanoparticles for potentiating cancer immunotherapy. Nano Lett. 23, 10350–10359 (2023). 37930173 10.1021/acs.nanolett.3c02941

[34] J. P. Liu, J. Z. Zhan, Y. Zhang, L. Huang, J. Yang, J. Feng, L. W. Ding, Z. Y. Shen, X. Y. Chen, Ultrathin clay nanoparticles-mediated mutual reinforcement of ferroptosis and cancer immunotherapy. Adv. Mater. 36, e2309562 (2024). 37939375 10.1002/adma.202309562

[35] J. Liu, Y. Zhang, B. Yang, Y. Jia, R. T. Liu, L. Ding, Z. Shen, X. Chen, Synergistic glutathione depletion and STING activation to potentiate dendritic cell maturation and cancer vaccine efficacy. Angew. Chem. Int. Ed. Engl. 63, e202318530 (2024). 38196070 10.1002/anie.202318530

[36] H. Jia, J. Lin, D. Wang, X. Lv, Q. Wang, Z. Wang, J. Liu, L. Yang, J. Liu, A Mn^2+^-assisted nanofiber-hydrogel adjuvant for simultaneous enhancement of humoral and cellular immune responses. Adv. Funct. Mater. 34, 2315442 (2024).

[37] H. Zhong, G. Chen, T. Li, J. Huang, M. Lin, B. Li, Z. Xiao, X. Shuai, Nanodrug augmenting antitumor immunity for enhanced TNBC therapy via pyroptosis and cGAS-STING activation. Nano Lett. 23, 5083–5091 (2023). 37220198 10.1021/acs.nanolett.3c01008

[38] D. Liu, S. Liang, K. Ma, Q. F. Meng, X. Li, J. Wei, M. Zhou, K. Yun, Y. Pan, L. Rao, X. Chen, Z. Wang, Tumor microenvironment-responsive nanoparticles amplifying STING signaling pathway for cancer immunotherapy. Adv. Mater. 36, 2304845 (2023). 10.1002/adma.202304845 37723642

[39] X. Li, S. Khorsandi, Y. Wang, J. Santelli, K. Huntoon, N. Nguyen, M. Yang, D. Lee, Y. Lu, R. Gao, B. Y. S. Kim, C. de Gracia Lux, R. F. Mattrey, W. Jiang, J. Lux, Cancer immunotherapy based on image-guided STING activation by nucleotide nanocomplex-decorated ultrasound microbubbles. Nat. Nanotechnol. 17, 891–899 (2022). 35637356 10.1038/s41565-022-01134-z PMC9378430

[40] R. Verbeke, I. Lentacker, L. Wayteck, K. Breckpot, M. Van Bockstal, B. Descamps, C. Vanhove, S. C. De Smedt, H. Dewitte, Co-delivery of nucleoside-modified mRNA and TLR agonists for cancer immunotherapy: Restoring the immunogenicity of immunosilent mRNA. J. Control. Release 266, 287–300 (2017). 28987878 10.1016/j.jconrel.2017.09.041

[41] G. Wang, J. Li, L. Wang, Y. Yang, J. Wu, W. Tang, H. Lei, L. Cheng, Manganese-doped potassium chloride nanoelectrodes to potentiate electrochemical immunotherapy. ACS Nano 18, 10885–10901 (2024). 38587876 10.1021/acsnano.4c01132

[42] J. Huang, P. Yu, M. Liao, X. Dong, J. Xu, J. Ming, D. Bin, Y. Wang, F. Zhang, Y. Xia, A self-charging salt water battery for antitumor therapy. Sci. Adv. 9, eadf3992 (2023). 37000876 10.1126/sciadv.adf3992 PMC10065443

[43] H. Chen, H. Kuang, F. Liu, Y. Wu, S. Cai, M. Xu, S.-J. Bao, A self-healing neutral aqueous rechargeable Zn/MnO_2_ battery based on modified carbon nanotubes substrate cathode. J. Colloid Interf. Sci. 600, 83–89 (2021). 10.1016/j.jcis.2021.04.097 34004432

[44] X. Guo, J. Zhou, C. Bai, X. Li, G. Fang, S. Liang, Zn/MnO_2_ battery chemistry with dissolution-deposition mechanism. Mater. Today Energy 16, 100396 (2020).

[45] S. Liu, T. Zeng, Z. He, M. Zuo, S. Chen, Y. Liu, Z. Fan, H. He, Q. Kong, Z. Zhou, L. Han, Synergistic effect of Ru single atoms and MnO_2_ to boost oxygen reduction/evolution activity via strong electronic interaction. Chem. Eng. J. 499, 156051 (2024).

[46] J. Xie, Y. Chen, Z. He, S. Liu, Y. Liu, B. Li, T. Xu, X. Ning, S. Chen, T. Zeng, H. He, Single-atom Ni anchored on α-MnO_2_ nanorods as an electrocatalyst for the oxygen evolution and oxygen reduction reactions. ACS Appl. Nano Mater. 7, 18027–18035 (2024).

[47] Z.-L. He, L.-Q. Wang, M. Jiang, J.-N. Xie, S. Liu, J.-C. Ren, R. Sun, W.-B. Lv, W.-B. Guo, Y.-L. Liu, B. Li, Q. Liu, H. He, Surface engineering on MnO_2_ nanorods by La single atoms to accelerate oxygen reduction kinetics. Rare Met. 43, 4302–4311 (2024).

[48] J. Lei, Y. Yao, Z. Wang, Y.-C. Lu, Towards high-areal-capacity aqueous zinc–manganese batteries: Promoting MnO_2_ dissolution by redox mediators. Energy Environ. Sci. 14, 4418–4426 (2021).

[49] W. Chen, G. Li, A. Pei, Y. Li, L. Liao, H. Wang, J. Wan, Z. Liang, G. Chen, H. Zhang, J. Wang, Y. Cui, A manganese–hydrogen battery with potential for grid-scale energy storage. Nat. Energy 3, 428–435 (2018).

[50] E. Mylod, G. Conlon, E. P. W. Jenkins, G. G. Malliaras, C. M. Gardiner, Tumor-treating fields increase cytotoxic degranulation of natural killer cells against cancer cells. Cell Rep. Phys. Sci. 5, 102119 (2024).

[51] Y. Wang, F. Gao, L. Zhao, Y. Wu, C. Li, H. Li, Y. Jiang, Enhancing cancer treatment via “Zn^2+^ interference” with Zn-based nanomaterials. Coord. Chem. Rev. 500, 215535 (2024).

[52] L. Ding, M. Liang, Y. Li, M. Zeng, M. Liu, W. Ma, F. Chen, C. Li, R. L. Reis, F. R. Li, Y. Wang, Zinc-organometallic framework vaccine controlled-release Zn^2+^ regulates tumor extracellular matrix degradation potentiate efficacy of immunotherapy. Adv. Sci. 10, 2302967 (2023). 10.1002/advs.202302967 PMC10520680 37439462

[53] X. Zeng, Z. Wang, A. Zhao, Y. Wu, Z. Wang, A. Wu, Q. Wang, X. Xia, X. Chen, W. Zhao, B. Li, Z. Lu, Q. Lv, G. Li, Z. Zuo, F. Wu, Y. Zhao, T. Wang, G. Nie, S. Li, G. Zhang, Zinc nanoparticles from oral supplements accumulate in renal tumours and stimulate antitumour immune responses. Nat. Mater. 24, 287–296 (2025). 39815063 10.1038/s41563-024-02093-7

[54] Y. Lv, X. Liu, J. Liu, S. Wu, S. Sun, P. Wu, Y. Wang, Y. Ding, Implantable and bio-compatible Na-O_2_ battery. Chem 10, 1885–1896 (2024).

[55] H. Wu, Y. Wang, H. Li, Y. Hu, Y. Liu, X. Jiang, H. Sun, F. Liu, A. Xiao, T. Chang, L. Lin, K. Yang, Z. Wang, Z. Dong, Y. Li, S. Dong, S. Wang, J. Chen, Y. Liu, D. Yin, H. Zhang, M. Liu, S. Kong, Z. Yang, X. Yu, Y. Wang, Y. Fan, L. Wang, C. Yu, L. Chang, Accelerated intestinal wound healing via dual electrostimulation from a soft and biodegradable electronic bandage. Nat. Electron. 7, 299–312 (2024).

[56] Y. Yin, X. Ge, J. Ouyang, N. Na, Tumor-activated in situ synthesis of single-atom catalysts for O_2_-independent photodynamic therapy based on water-splitting. Nat. Commun. 15, 2954 (2024). 38582750 10.1038/s41467-024-46987-1 PMC11258260

[57] L. Liu, H. Lei, G. Hou, L. Zhang, Y. Chen, Y. Lu, Z. Pei, J. Ge, J. Wu, J. Zhou, L. Cheng, Gas-amplified metalloimmunotherapy with dual activation of pyroptosis and the STING pathway for remodeling the immunosuppressive cervical cancer microenvironment. ACS Nano 18, 12830–12844 (2024). 38709246 10.1021/acsnano.4c00017

[58] Y. Huang, J. Zou, J. Huo, M. Zhang, Y. Yang, Sulfate radical based in situ vaccine boosts systemic antitumor immunity via concurrent activation of necroptosis and STING pathway. Adv. Mater. 36, e2407914 (2024). 39148154 10.1002/adma.202407914

[59] D. Li, E. Ha, Z. Zhou, J. Zhang, Y. Zhu, F. Ai, L. Yan, S. He, L. Li, J. Hu, “Spark” PtMnIr nanozymes for electrodynamic-boosted multienzymatic tumor immunotherapy. Adv. Mater. 36, e2308747 (2024). 38108600 10.1002/adma.202308747

[60] H. Li, C. Chen, Z. Wang, Y. Huang, G. He, Y. Liu, P. Jiang, Z. L. Wang, Triboelectric immunotherapy using electrostatic-breakdown induced direct-current. Mater. Today 64, 40–51 (2023).

[61] W. Q. Huang, Y. Q. Zhu, F. Gao, W. You, G. Chen, X. Nie, L. Xia, L. H. Wang, C. Y. Hong, Z. Zhang, F. Wang, Y. Yu, Y. Z. You, Nanogalvanic cells release highly reactive electrons in tumors to effectively eliminate tumors. Adv. Mater. 36, 2404199 (2024). 10.1002/adma.202404199 38734974

