# Supplementary material for: Tumor microenvironment–activated Zn//MnO2 battery for sustained and local electrochemical immunotherapy
Source: Sci Adv. 2025 May 28;11(22):eadu1647. doi: 10.1126/sciadv.adu1647 (PMC12118630; doi:10.1126/sciadv.adu1647)
Supplement: Supplementary file 1 — Figs. S1 to S51 Tables S1 and S2 References [file sciadv.adu1647_sm.pdf]

Science Advances
AAAS

Supplementary Materials for

Tumor microenvironment–activated Zn//MnO_2 battery for sustained and local electrochemical immunotherapy

Xiaoran Ding *et al.*

Corresponding author: Xiaoteng Jia, xtjia@jlu.edu.cn; Meiying Xin, myxin@jlu.edu.cn;
Caiyun Wang, caiyun@uow.edu.au

Sci. Adv. **11**, eadu1647 (2025)
DOI: 10.1126/sciadv.adu1647

This PDF file includes:

Figs. S1 to S51
Tables S1 and S2
References

Supplementary Figures

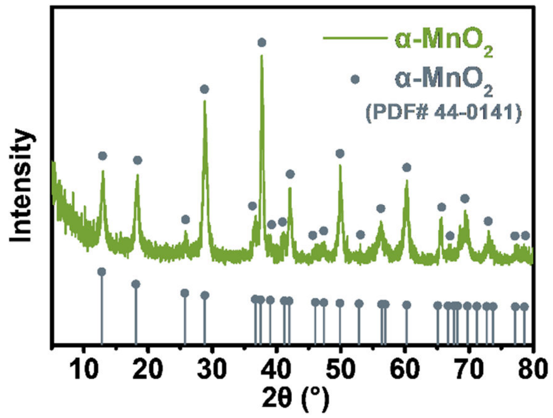

fig. S1. XRD pattern of the pristine α-MnO_2 electrode.

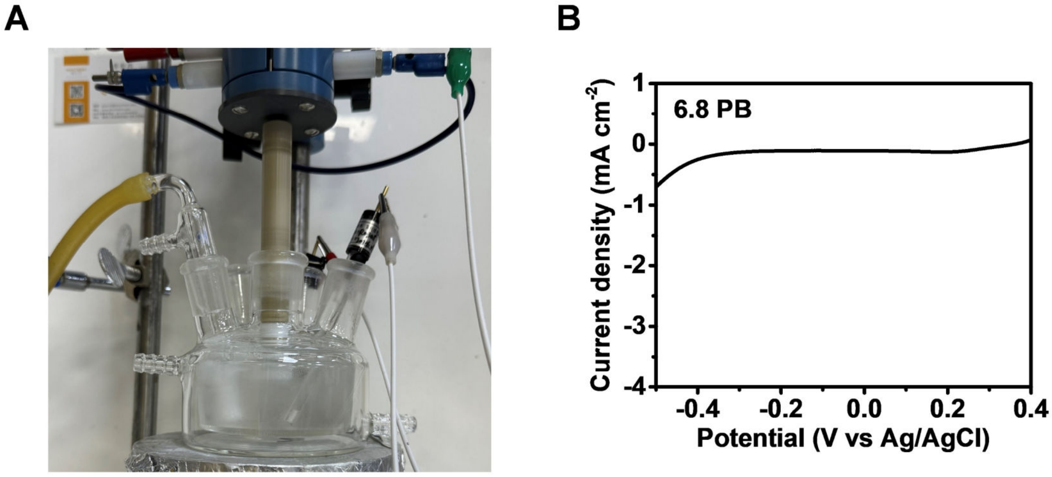

fig. S2. ORR performance of the MnO_2 electrode. (**A**) Schematic diagram of the rotating disk electrode. (**B**) The LSV curve recorded at 1600 rpm in an O_2-saturated PB electrolyte (pH = 6.8).

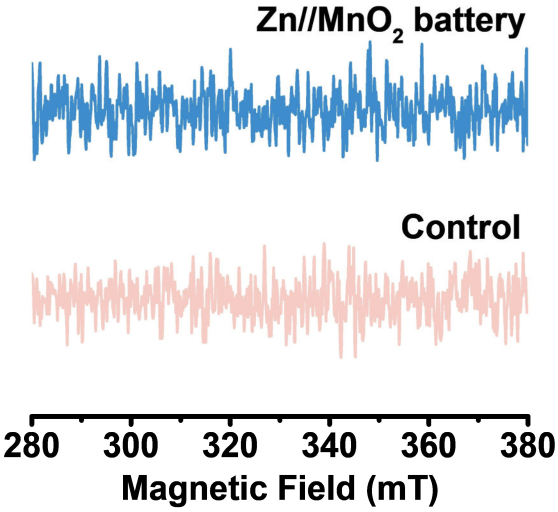

fig. S3. Electron spin resonance spectra of the solution after the battery discharge for 12 h in a full O_2 environment (6.8 PB). The capture agent is DMPO.

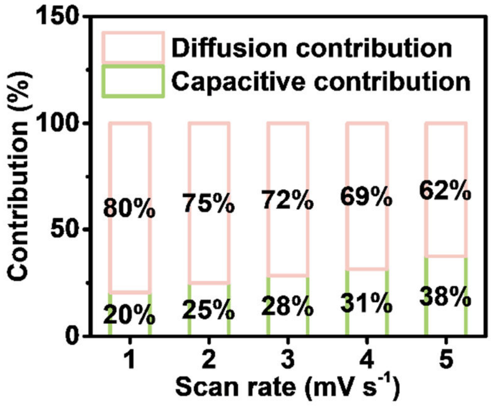

fig. S4. Capacity contribution ratios at different scan rates of Zn//MnO_2 battery in 2 M $ZnSO_4$ and 0.5 M $MnSO_4$ electrolytes.

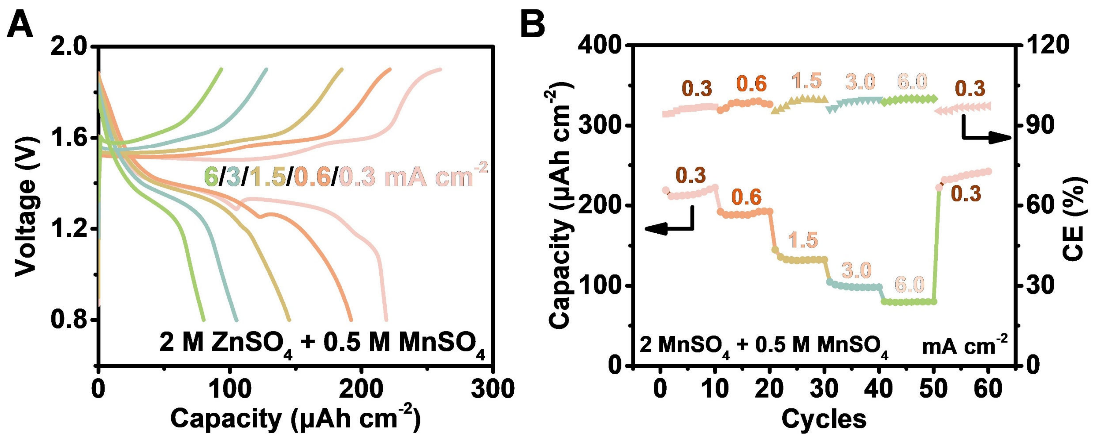

fig. S5. Electrochemical performance of Zn//MnO_2. (**A**) Galvanostatic charge/discharge curves of coin cell with α-MnO_2 cathode, Zn anode in 2 M $ZnSO_4$ and 0.5 M $MnSO_4$ electrolytes under different charge/discharge current density from 0.3-6 mA cm^{-2}. (**B**) Rate performance of Zn//MnO_2 coin cell.

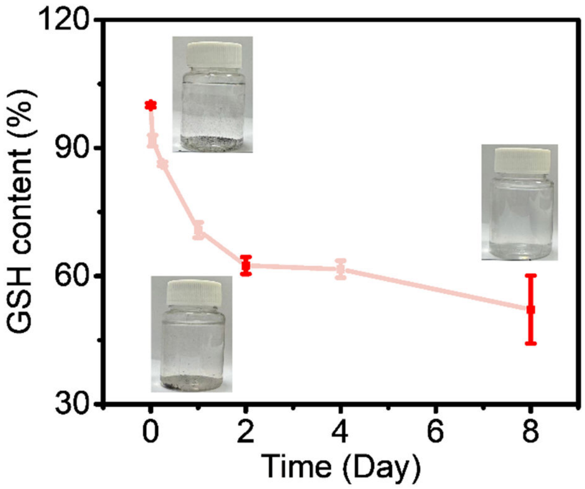

fig. S6. The changes of GSH content detected by DTNB solution. The illustrations were the samples on Day 0, Day 2, and Day 8. All dates are expressed as mean ± SD (n = 3).

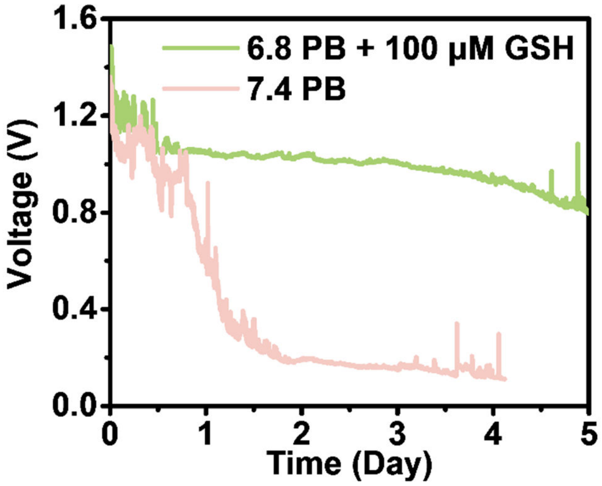

fig. S7. Time-resolved discharge curves of $Zn//MnO_2$ at a fixed value resistance (100 kohm).

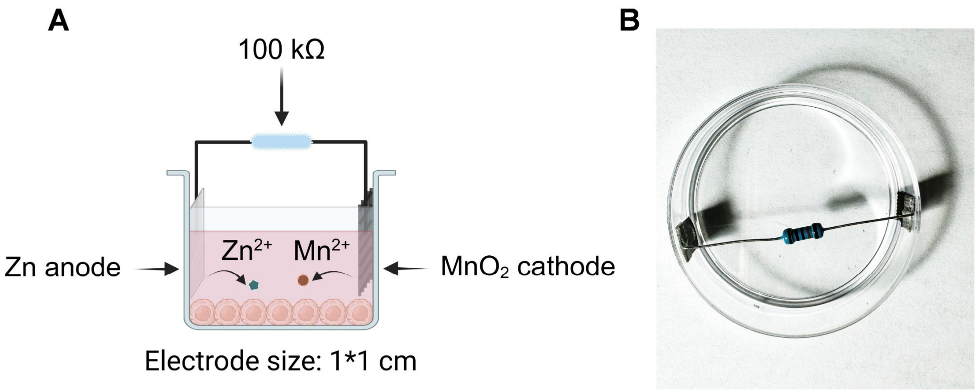

fig. S8. *In vitro* device structure of the Zn//MnO_2 battery. (**A**) Schematic diagrams and (**B**) photographs.

Structure of Zn//MnO_2 battery *in vitro*: The Zn and MnO_2 electrodes were 1.0 cm by 1.0 cm in size. The resistor was connected to the electrodes with silver paste and the joints were sealed with PDMS to ensure connection stability. The electrolyte was the completed DMEM medium.

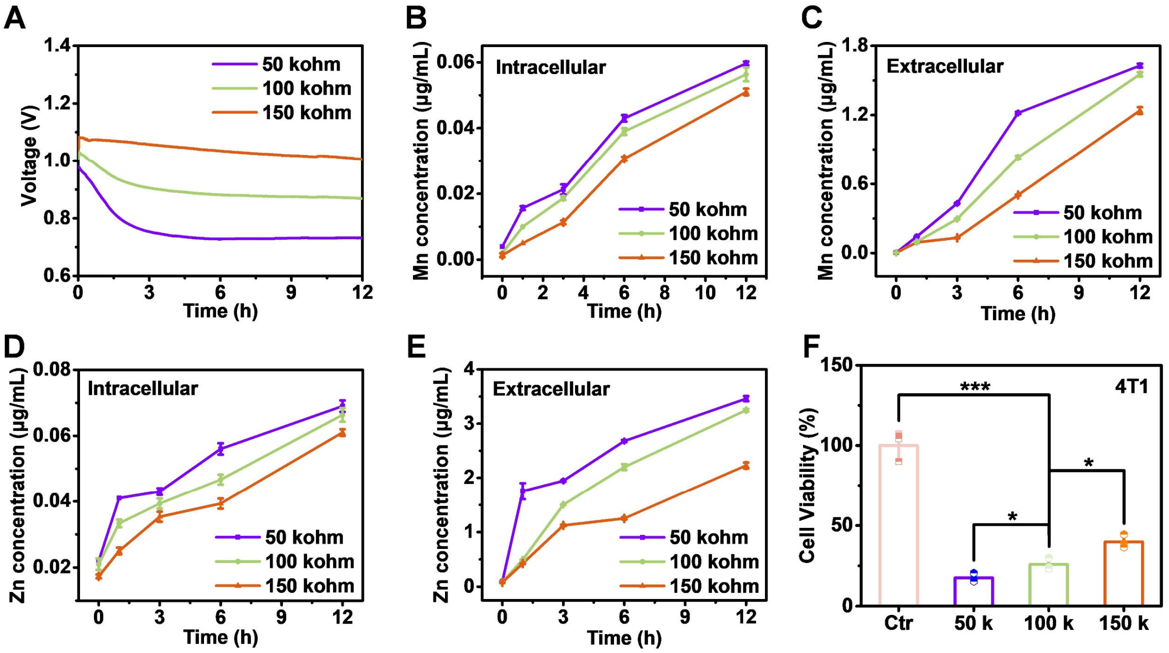

fig. S9. The screening of the resistance value (50 kohm, 100 kohm, and 150 kohm). (**A**) The discharge curves of Zn//MnO_2 battery connected with different resistors. (**B**, **C**) The concentration of Mn ions at different time points. (**D**, **E**) The concentration of Zn ions at different time points. (**F**) The cell viability of 4T1 cells cultured for 12 h. All dates are expressed as mean ± SD (n = 3), and the differences were assessed by one-way ANOVA with Tukey's post-test. *$p < 0.05$ and ***$p < 0.001$.

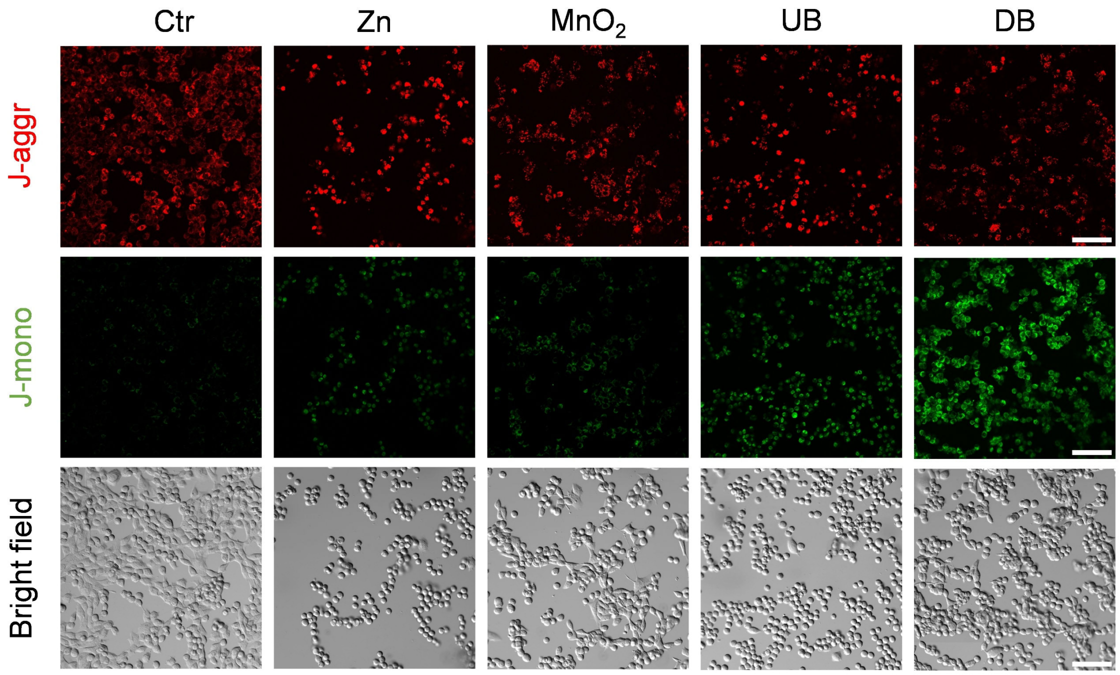

fig. S10. Fluorescence images showing the change of mitochondrial membrane potential after incubation with different treatments. Scale bar, 100 µm.

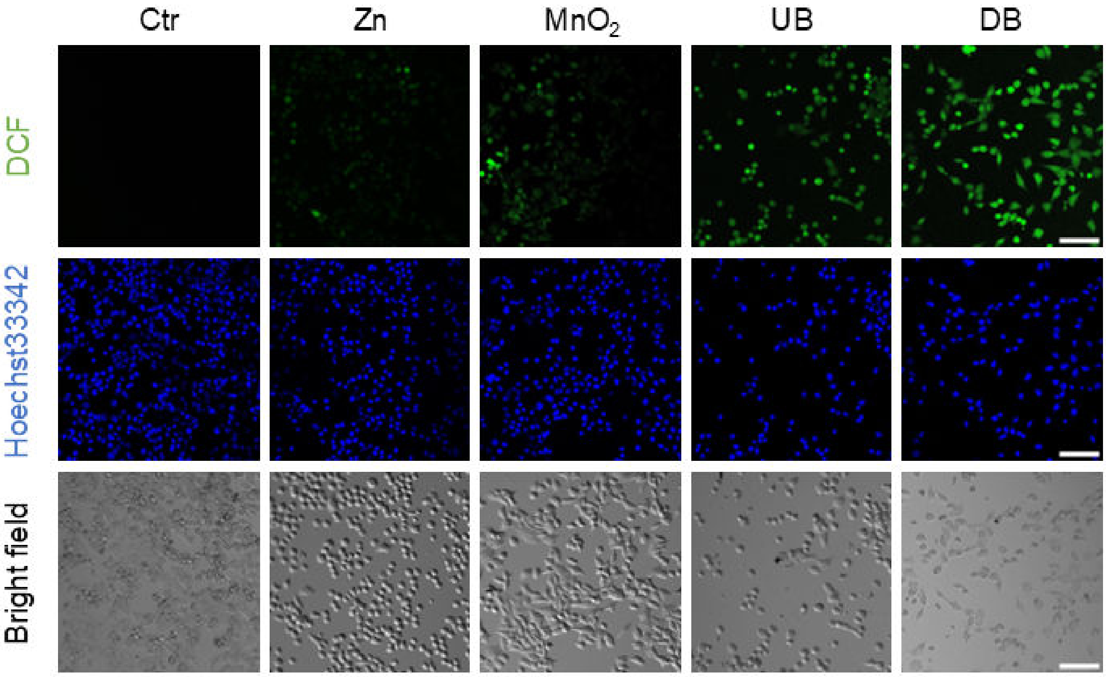

fig. S11. Representative confocal images of ROS after different treatments. Scale bar, 100 μm.

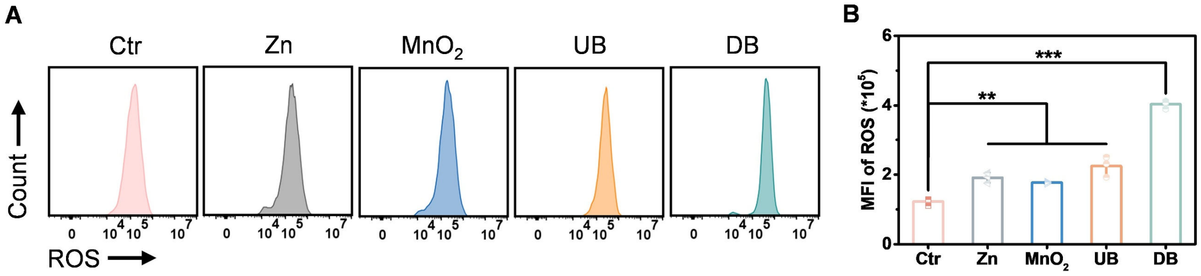

fig. S12. Determination of ROS in microenvironment after various treatments. (**A**) Flow cytometry analysis and (**B**) mean fluorescence intensity (MFI) of ROS level. All dates are expressed as mean ± SD (n = 3), and the differences were assessed by one-way ANOVA followed by Dunnett's multiple comparisons tests. $^{**}p < 0.01$ and $^{***}p < 0.001$.

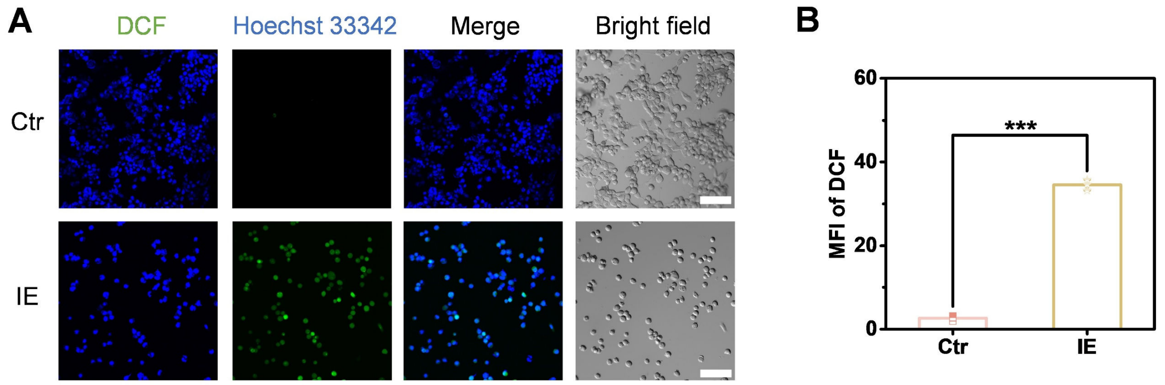

fig. S13. Discharge current-mediated microenvironment regulation using inert electrodes (IE) on 4T1 cells. (**A**) Representative confocal images and (**B**) quantitative fluorescence intensity of ROS. Scale bar, 100 μm. All dates are expressed as mean ± SD (n = 3), and the differences were assessed by two-tailed Student's *t*-test. ***$p < 0.001$.

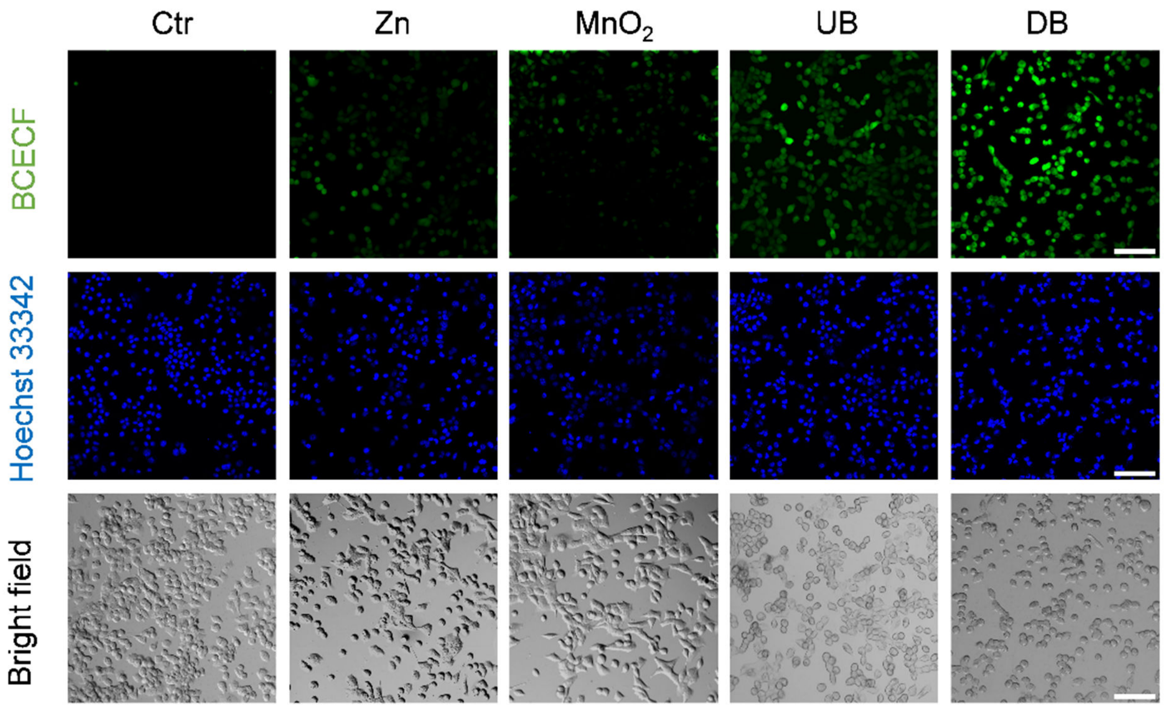

fig. S14. Fluorescence images of intracellular pH change after being treated with various groups. Scale bar, 100 μm.

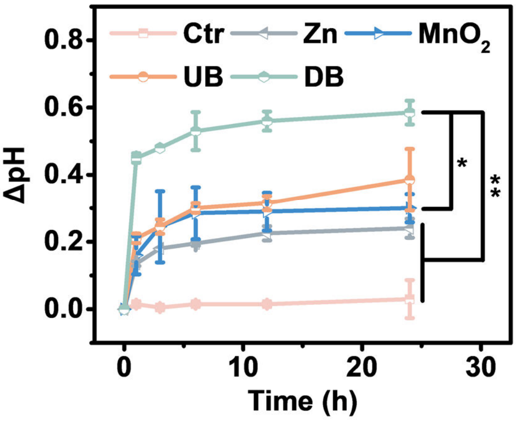

fig. S15. The pH changes in cell supernatant detected by a pH meter. All dates are expressed as mean ± SD (n = 3), and the differences were assessed by one-way ANOVA followed by Dunnett's multiple comparisons tests. $*p < 0.05$ and $**p < 0.01$.

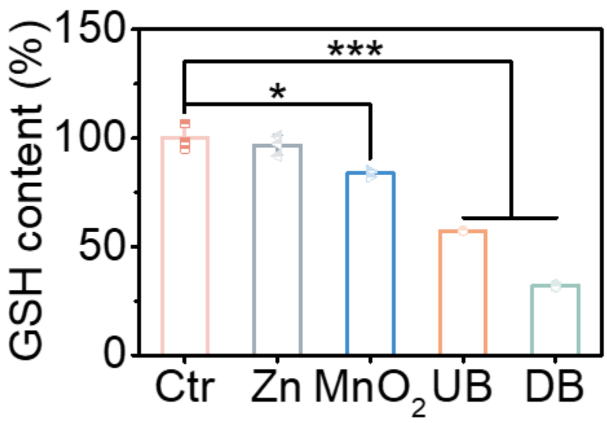

fig. S16. The GSH changes detected by DTNB solution. All dates are expressed as mean ± SD (n = 3), and the differences were assessed by one-way ANOVA followed by Dunnett's multiple comparisons tests. *$p < 0.05$ and ***$p < 0.001$.

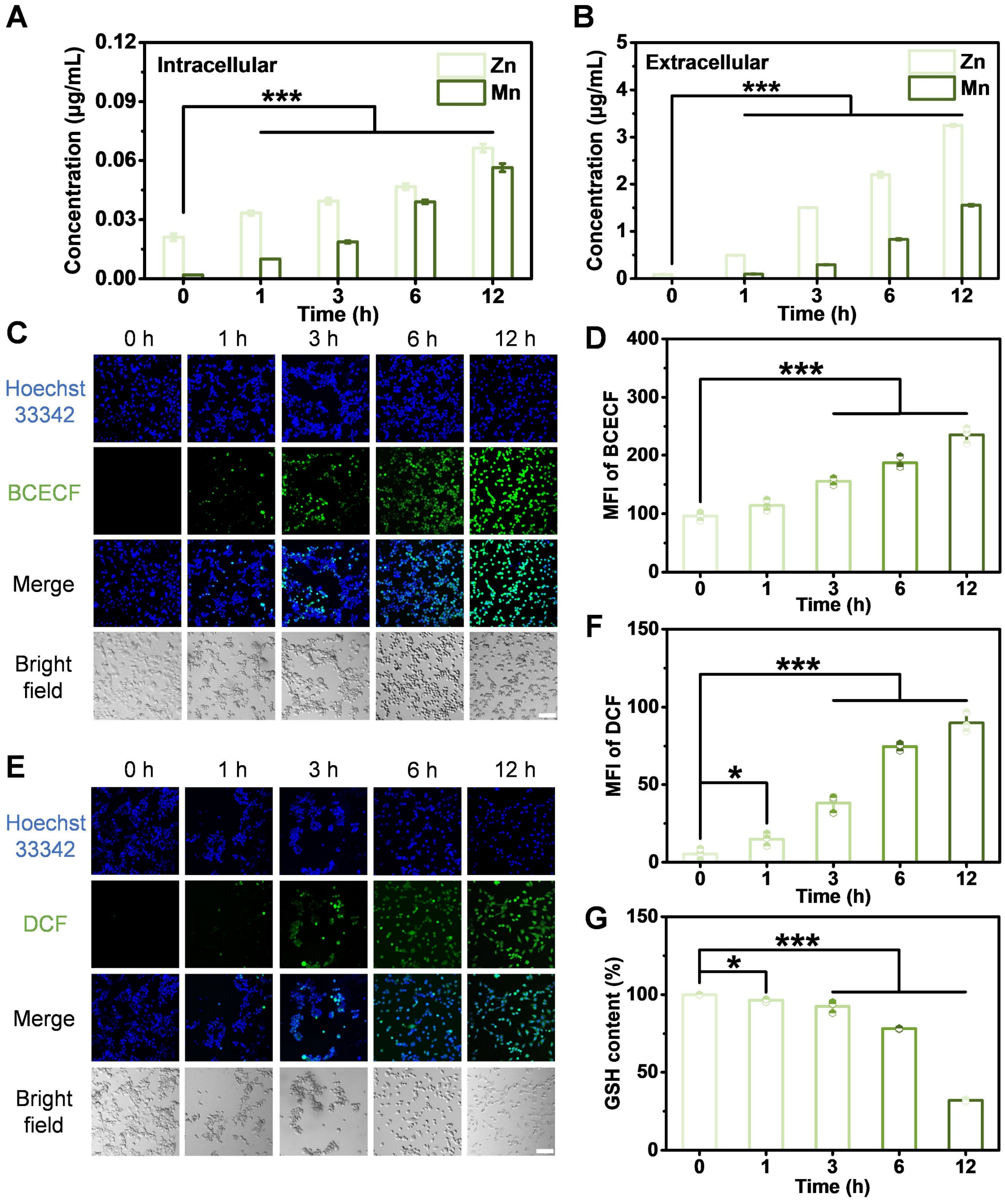

fig. S17. Battery-induced dynamic regulation of the microenvironment. (**A**) The intracellular and (**B**) the extracellular concentrations of Zn and Mn ions. (**C**) Representative confocal images and (**D**) quantitative fluorescence intensity of cell-permeant pH changes at different time points. (**E**) Fluorescence images and (**F**) corresponding quantitative intensity of ROS at different time points. (**G**) The changes of GSH. Scale bar, 100 μm. All dates are expressed as mean ± SD (n = 3), and the differences were assessed by one-way ANOVA followed by Dunnett's multiple comparisons tests. *p < 0.05 and ***p < 0.001.

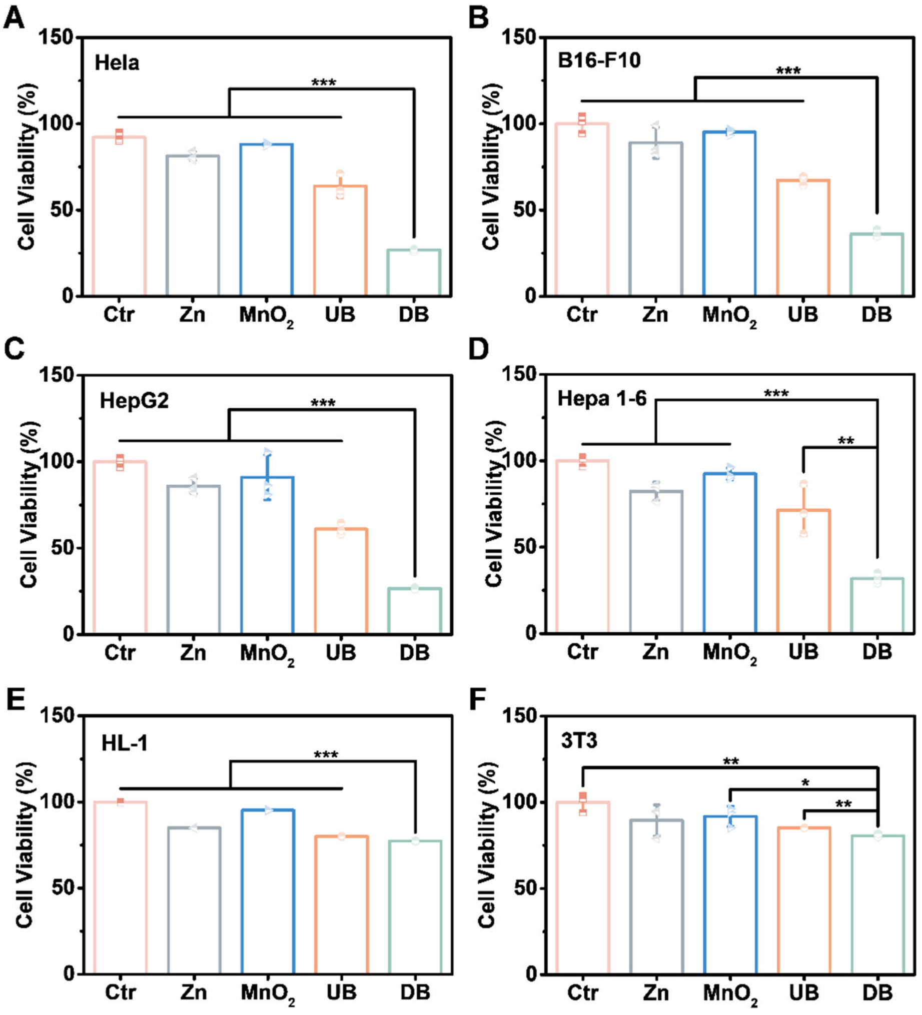

fig. S18. Cell viability incubated with different treatments by the MTT assay. (**A**) Hela cells, (**B**) B16-F10 cells, (**C**) HepG2 cells, (**D**) Hepa1-6 cells, (**E**) HL-1 cells, and (**F**) 3T3 cells. All dates are expressed as mean ± SD (n = 3), and the differences were assessed by one-way ANOVA followed by Dunnett's multiple comparisons tests. *$p < 0.05$, **$p < 0.01$ and ***$p < 0.001$.

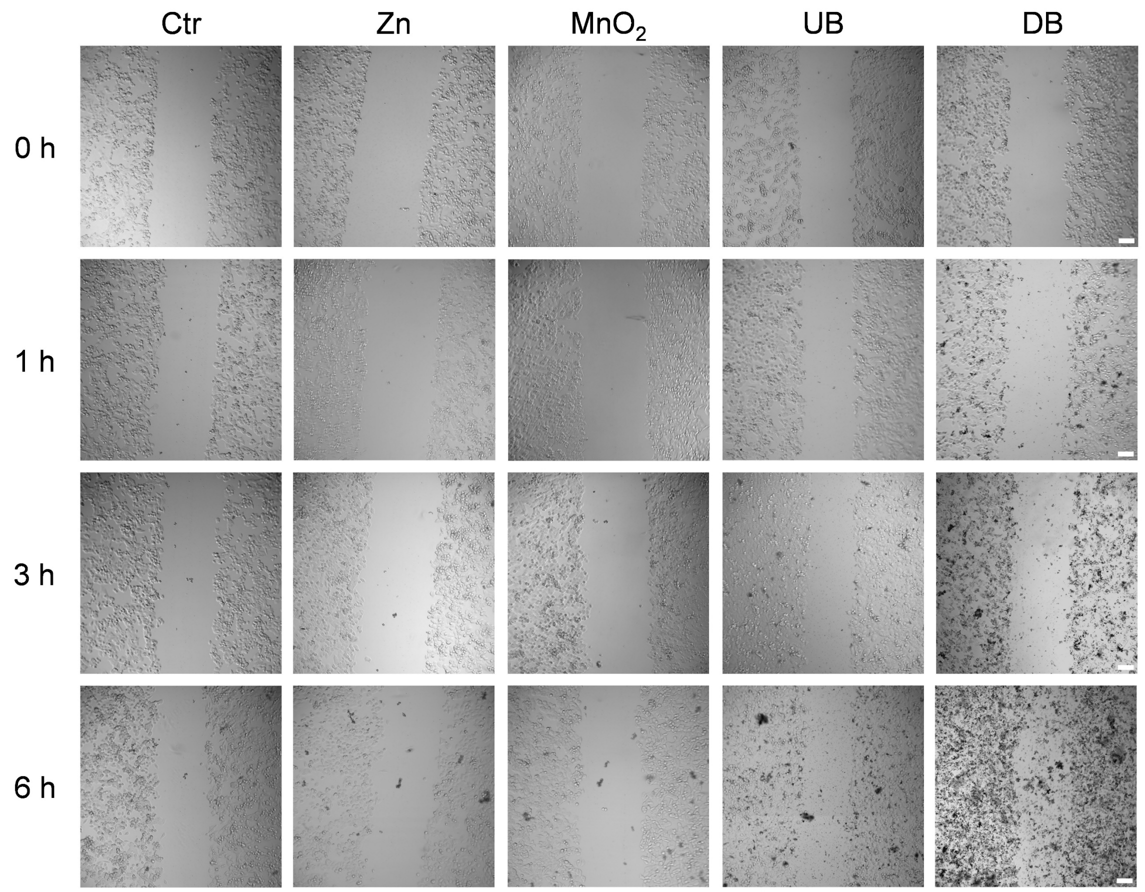

fig. S19. Optical micrographs of 4T1 cells migration at different incubation times with various treatments. Scale bar, 100 μm.

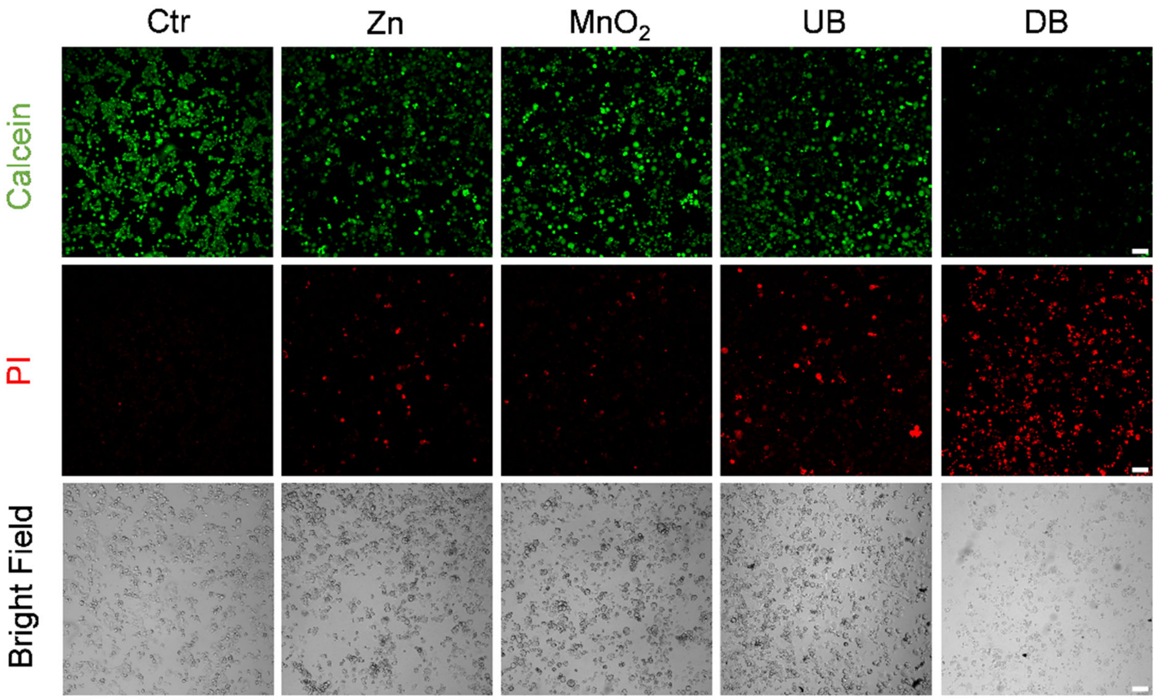

fig. S20. Confocal microscopy images of 4T1 cells stained with Calcein-AM&PI after various treatments. Scale bar, 100 µm.

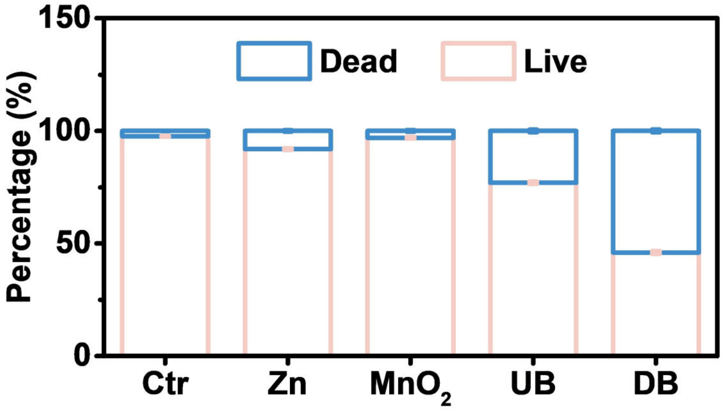

fig. S21. The statistical graph of live and dead 4T1 cells after various treatments using Annexin V and PI. All dates are expressed as mean ± SD (n = 3).

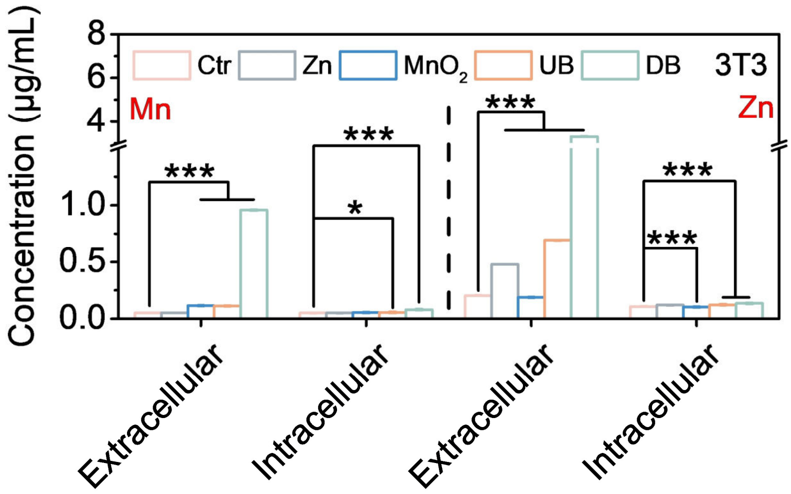

fig. S22. The concentrations of Zn and Mn ions in the supernatant and cytochylema of 3T3 cells treated with different groups. All dates are expressed as mean ± SD (n = 3), and the differences were assessed by one-way ANOVA followed by Dunnett's multiple comparisons tests. *$p < 0.05$ and ***$p < 0.001$.

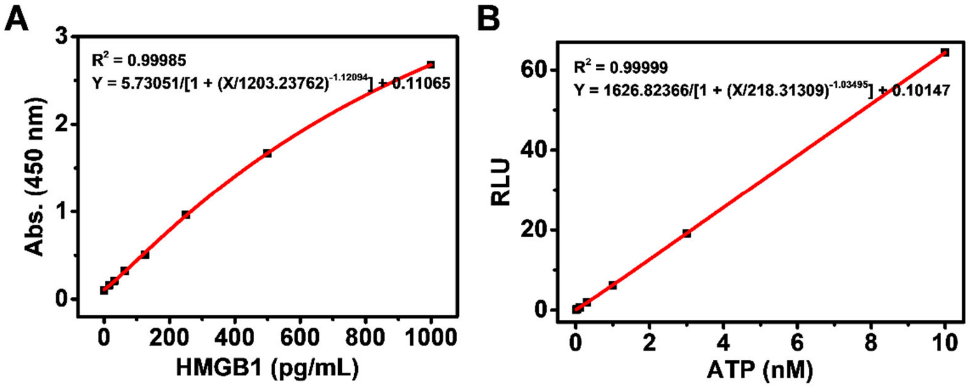

fig. S23. The standard absorbance curves. (**A**) HMGB1 and (**B**) ATP.

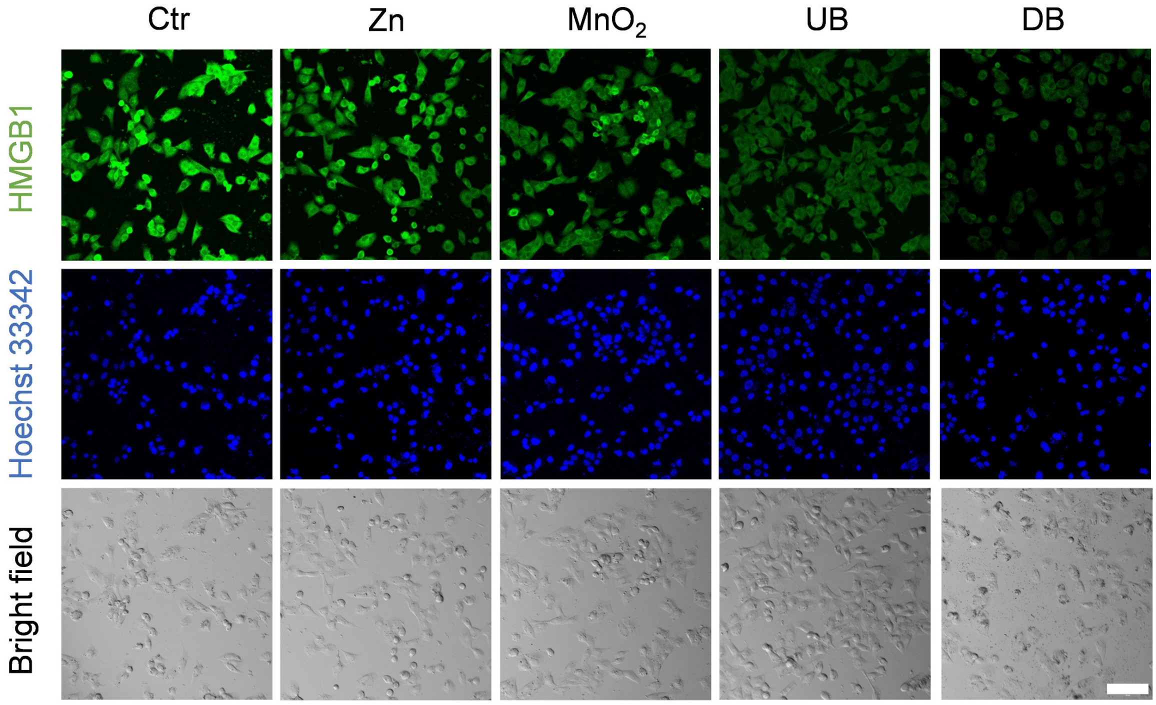

fig. S24. Representative confocal images of HMGB1 after different treatments. Scale bar, 100 μm.

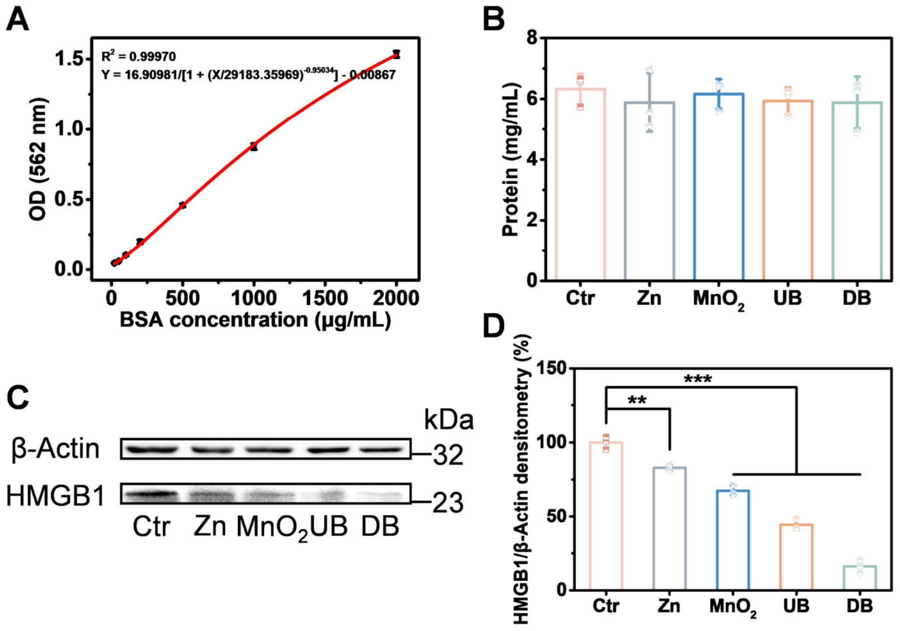

fig. S25. Intracellular levels of HMGB1 in 4T1 cells incubated with different groups. (**A**) The standard curve of BSA. (**B**) The concentrations of protein. (**C**) Western blot analysis and (**D**) quantitative analysis of HMGB1. All dates are expressed as mean ± SD (n = 3), and the differences were assessed by one-way ANOVA followed by Dunnett's multiple comparisons tests. $^{**}p < 0.01$ and $^{***}p < 0.001$.

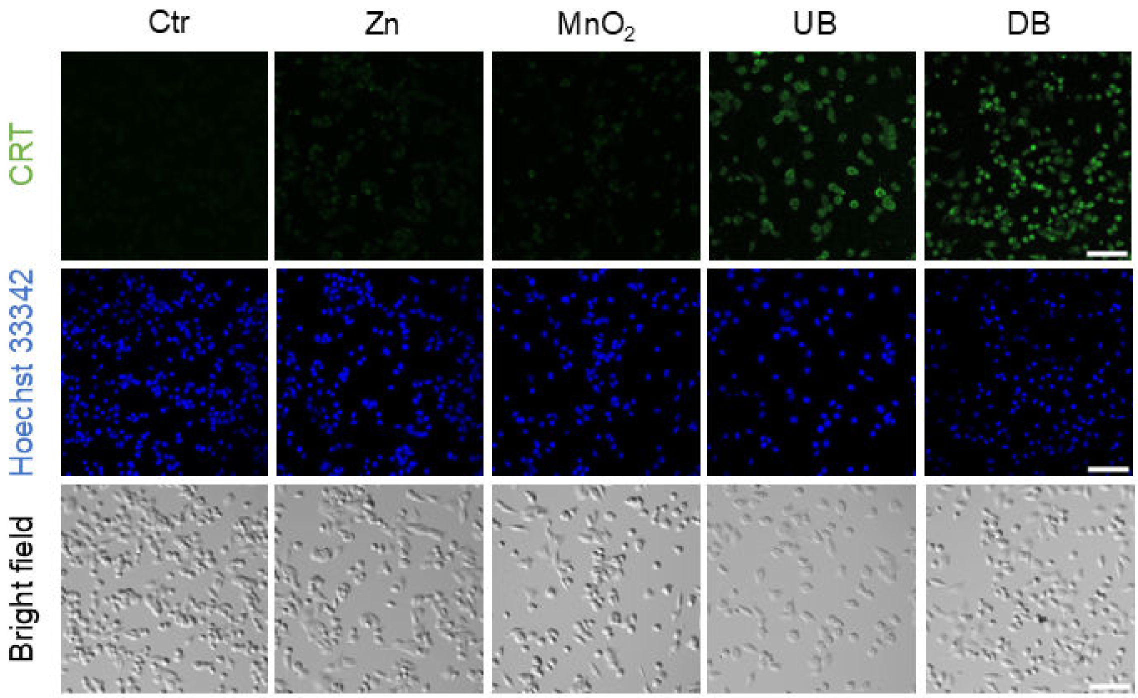

fig. S26. Representative confocal images of CRT after different treatments. Scale bar, 100 μm.

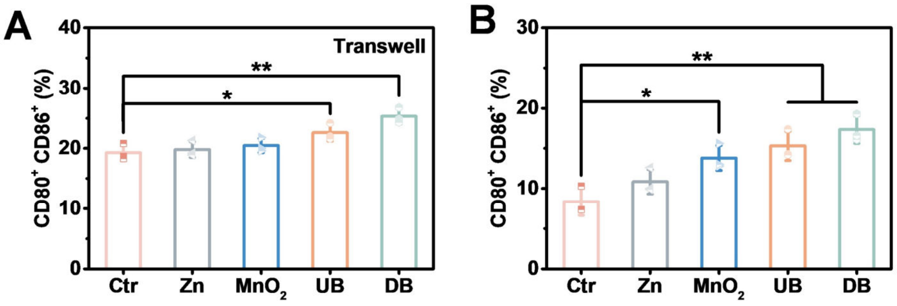

fig. S27. Quantification of BMDCs maturation stimulated by different methods. (**A**) 4T1 cells and (**B**) various treatment groups. All dates are expressed as mean ± SD (n = 3), and the differences were assessed by one-way ANOVA followed by Dunnett's multiple comparisons tests. $*p < 0.05$ and $**p < 0.01$.

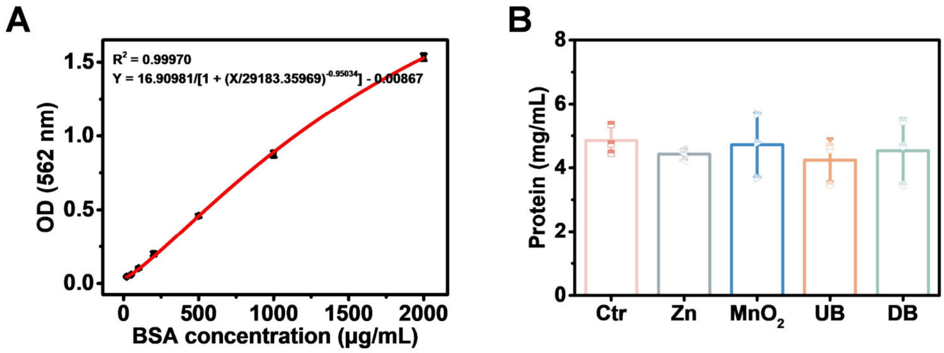

fig. S28. The determination of mature BMDCs incubated with various treatments. (**A**) The standard absorbance curve of BSA at 562 nm. (**B**) The total protein content. All dates are expressed as mean ± SD (n = 3).

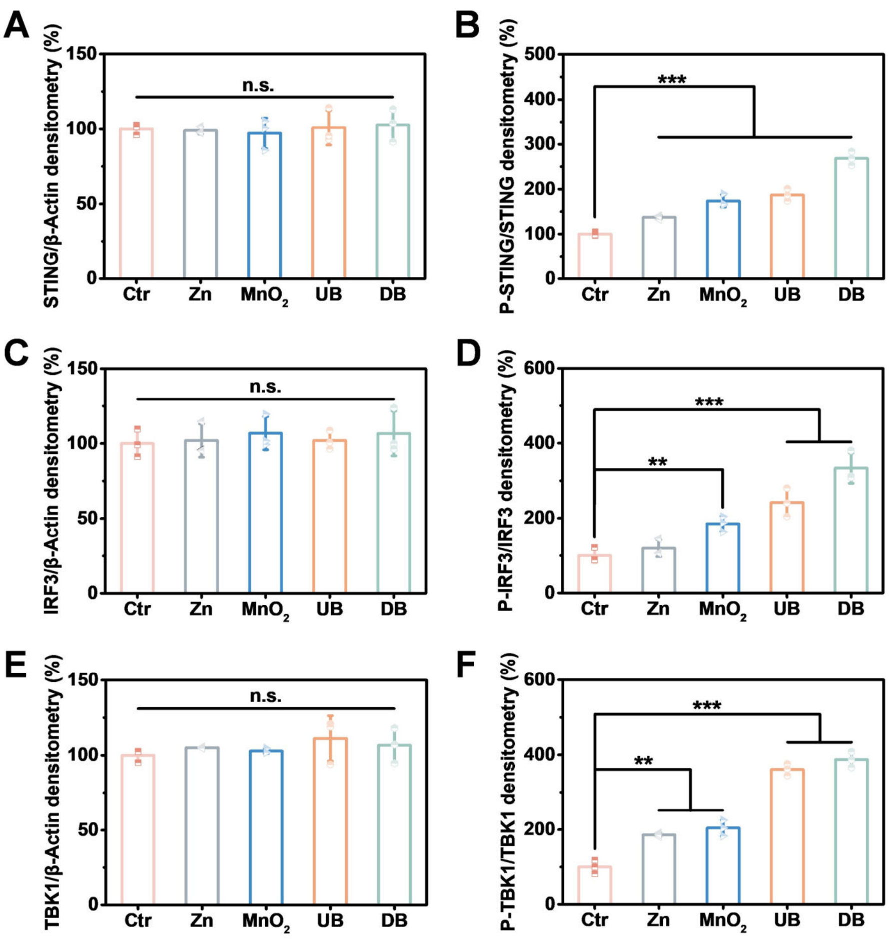

fig. S29. Quantitative analysis of the proteins. (**A**) STING, (**B**) P-STING, (**C**) IRF3, (**D**) P-IRF3, (**E**) TBK1, and (**F**) P-TBK1. All dates are expressed as mean ± SD (n = 3), and the differences were assessed by one-way ANOVA followed by Dunnett's multiple comparisons tests. n.s.: no significance, $p > 0.05$, $**p < 0.01$ and $***p < 0.001$.

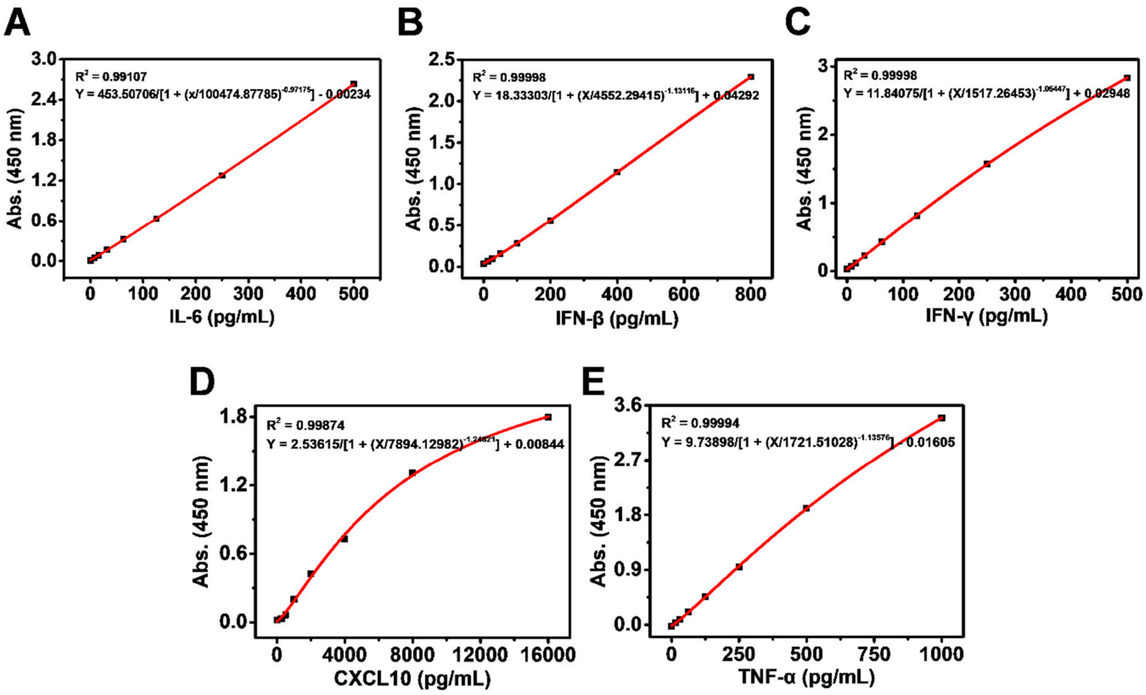

fig. S30. The standard absorbance curves at 450 nm. (**A**) IL-6, (CXCL10, **B**) IFN-β, (**C**) IFN-γ, (**D**) and (**E**) TNF-α.

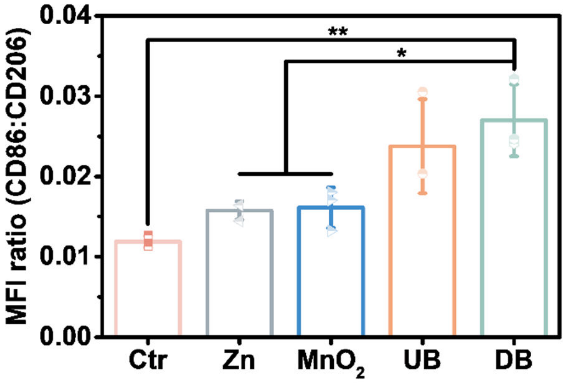

fig. S31. Quantification of the M1- and M2-type macrophage in BMDMs after various treatments. All dates are expressed as mean ± SD (n = 3), and the differences were assessed by one-way ANOVA with Tukey's post-test. **p* < 0.05 and ***p* < 0.01.

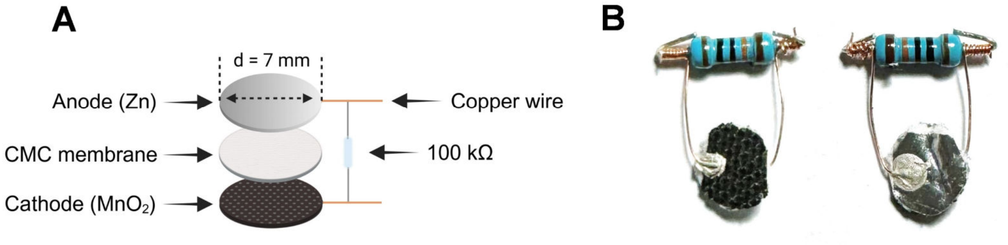

fig. S32. ***In vivo*** **device structure of the Zn//MnO$_2$ battery.** (**A**) Schematic diagram and (**B**) photographs.

Structure of Zn//MnO$_2$ battery *in vivo*: The Zn and MnO$_2$ electrodes were circular each with a diameter of 0.7 cm. They were separated by a CMC membrane and the adhesion was achieved using a glass adhesive (Permatex8173) with good biocompatibility. Copper wires were connected to the electrodes with silver paste and the joints were sealed with PDMS to ensure connection stability. The other two ends of the copper wires were connected to a 100 kohm resistor. The electrolyte was the body fluid at the implantation site.

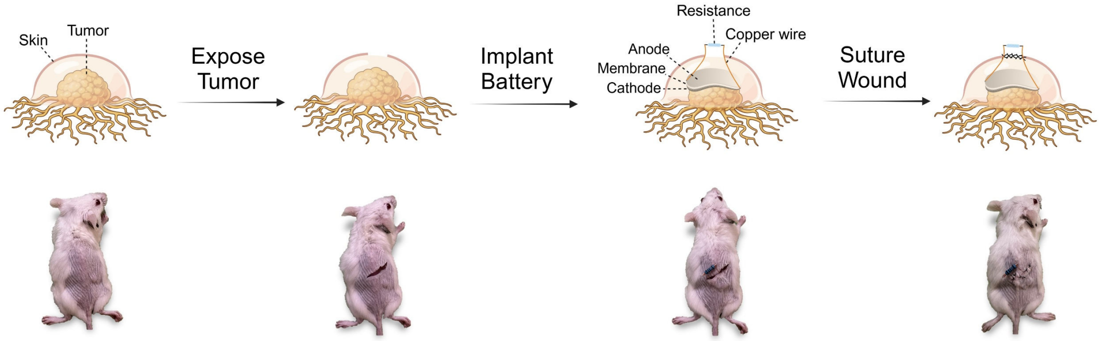

fig. S33. Schematic diagram of the Zn//MnO_2 battery implantation *in vivo*. Mn ions were the main agonists for regulating the tumor microenvironment, therefore the cathode was attached to the tumor. The Zn anode, not attached to the tumor surface, was also electrochemically corroded in the tumor microenvironment to play its role.

The experimental procedures were as follows:

① Anesthesia: 2,2,2-tribromoethanol anesthetic (2.5 wt%) was intraperitoneally injected into mice at a dose of 100 μL/10 g.

② Exposure: Used surgical scissors to make an incision on the skin near the tumor on the back of the mouse. Then, gently cleared the subcutaneous mucosa with surgical forceps to expose the tumor tissue and provide space for implantation for the battery.

③ Implantation: Attached the MnO_2 cathode of the battery to the tumor surface, and placed the resistor outside the body. The MnO_2 electrode adhered to the remaining subcutaneous mucosa, eliminating the need for any additional operations. To ensure that the implanted battery is securely attached to the tumor, the cleaned mucosa area should be minimal just enough to accommodate the battery.

④ Suture: The wound was sutured with a non-absorbable surgical suture and disinfected with povidone-iodine.

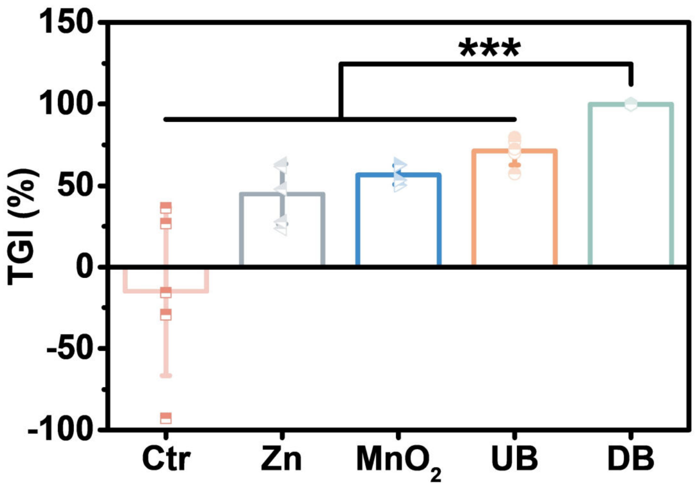

fig. S34. TGI rate of tumor after various treatments. All dates are expressed as mean ± SD (n = 5), and the differences were assessed by one-way ANOVA with Tukey's post-test. ***p < 0.001.

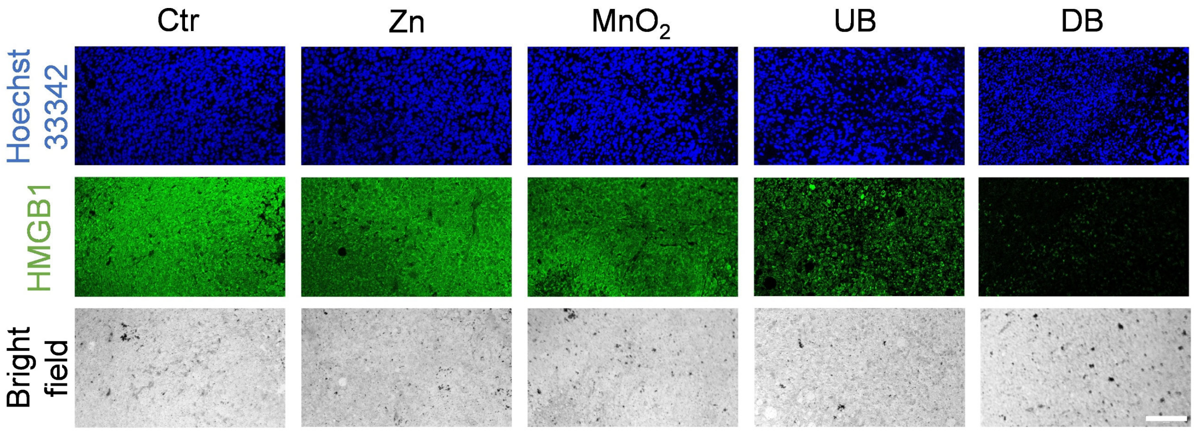

fig. S35. Representative confocal images of HMGB1 in tumor after various treatments *in vivo*. Scale bar, 100 μm.

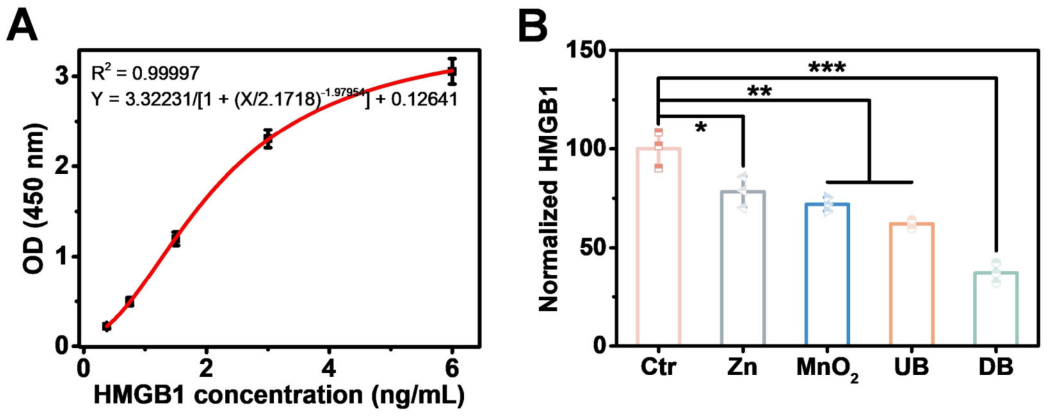

fig. S36. Intracellular levels of HMGB1 in tumors after various treatments *in vivo*. (**A**) The standard curve. (**B**) Normalized HMGB1. All dates are expressed as mean ± SD (n = 3), and the differences were assessed by one-way ANOVA followed by Dunnett's multiple comparisons tests. **p* < 0.05, ***p* < 0.01 and ****p* < 0.001.

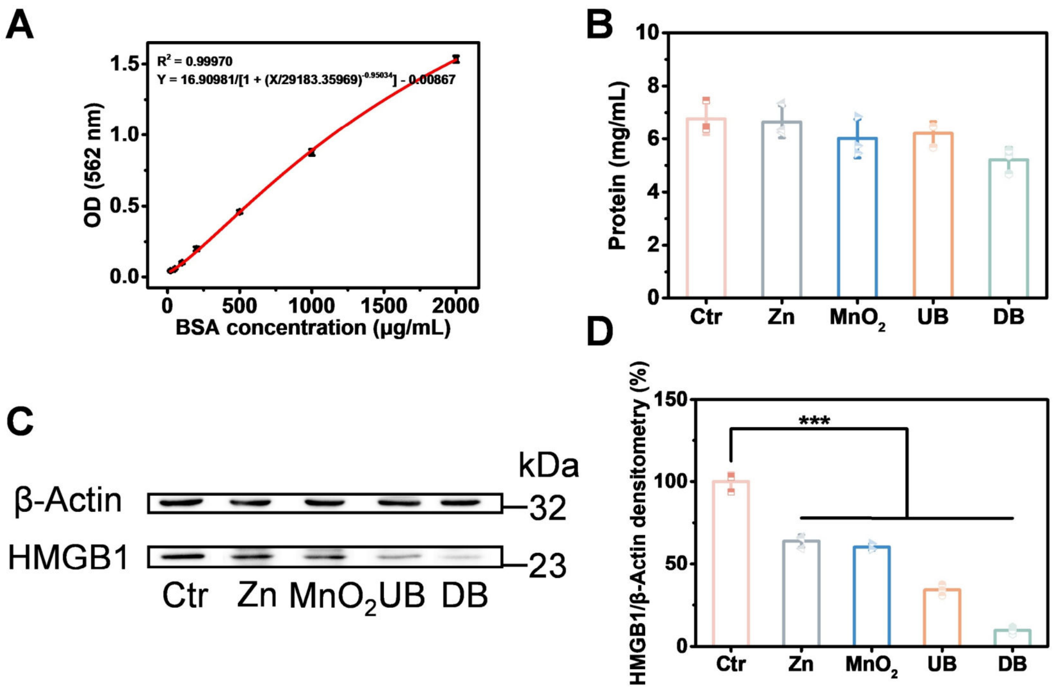

fig. S37. The intracellular levels of HMGB1 in tumors after being treated with different groups. (A) The standard curves of BSA. **(B)** The concentrations of protein. (**C**) Western blot analysis and (**D**) quantitative analysis of HMGB1. All dates are expressed as mean ± SD (n = 3), and the differences were assessed by one-way ANOVA followed by Dunnett's multiple comparisons tests. ***$p < 0.001$.

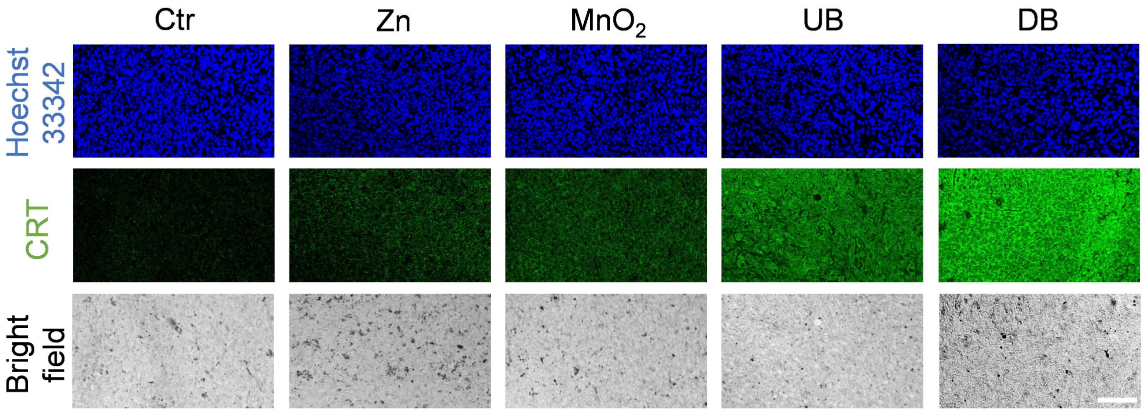

fig. S38. Confocal images of CRT in tumor after various treatments *in vivo*. Scale bar, 100 μm.

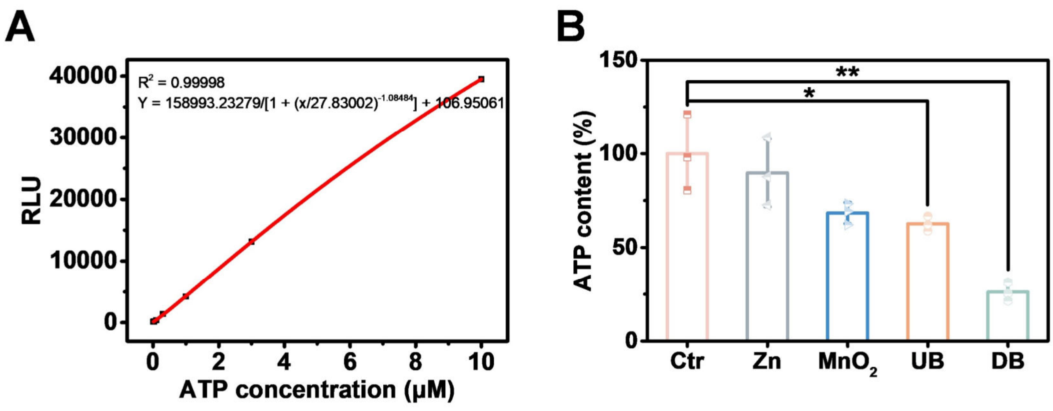

fig. S39. ATP levels in tumors after various treatments *in vivo*. (**A**) The standard curve. (**B**) The ATP content. All dates are expressed as mean ± SD (n = 3), and the differences were assessed by one-way ANOVA followed by Dunnett's multiple comparisons tests. *$p < 0.05$ and **$p < 0.01$.

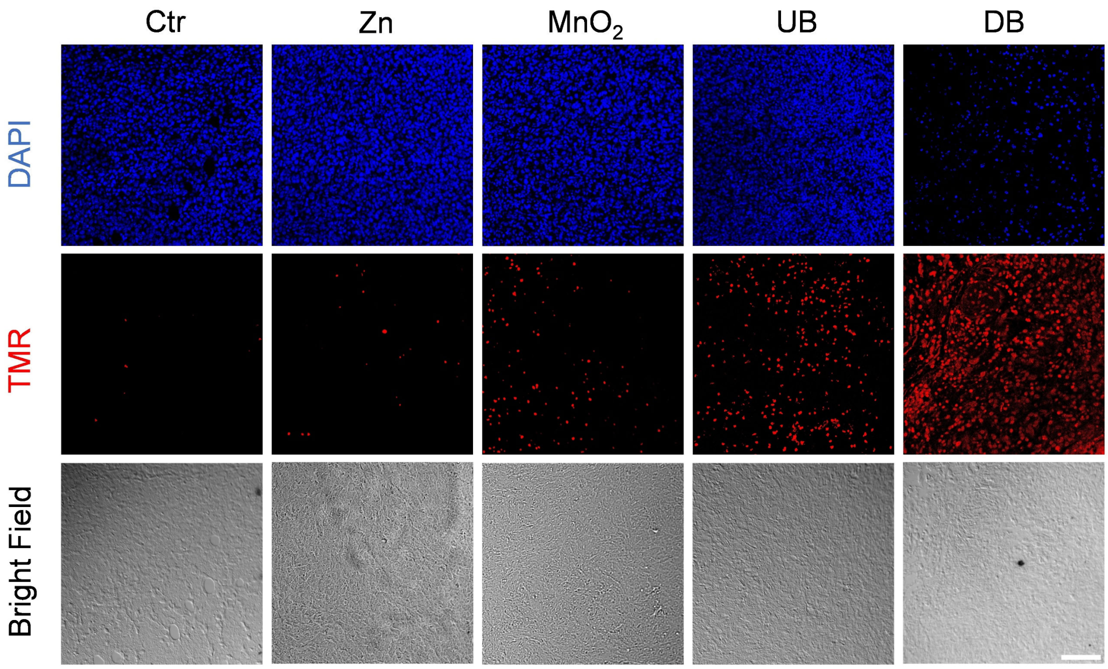

fig. S40. Representative confocal images of TUNEL after different treatments. Scale bar, 100 μm.

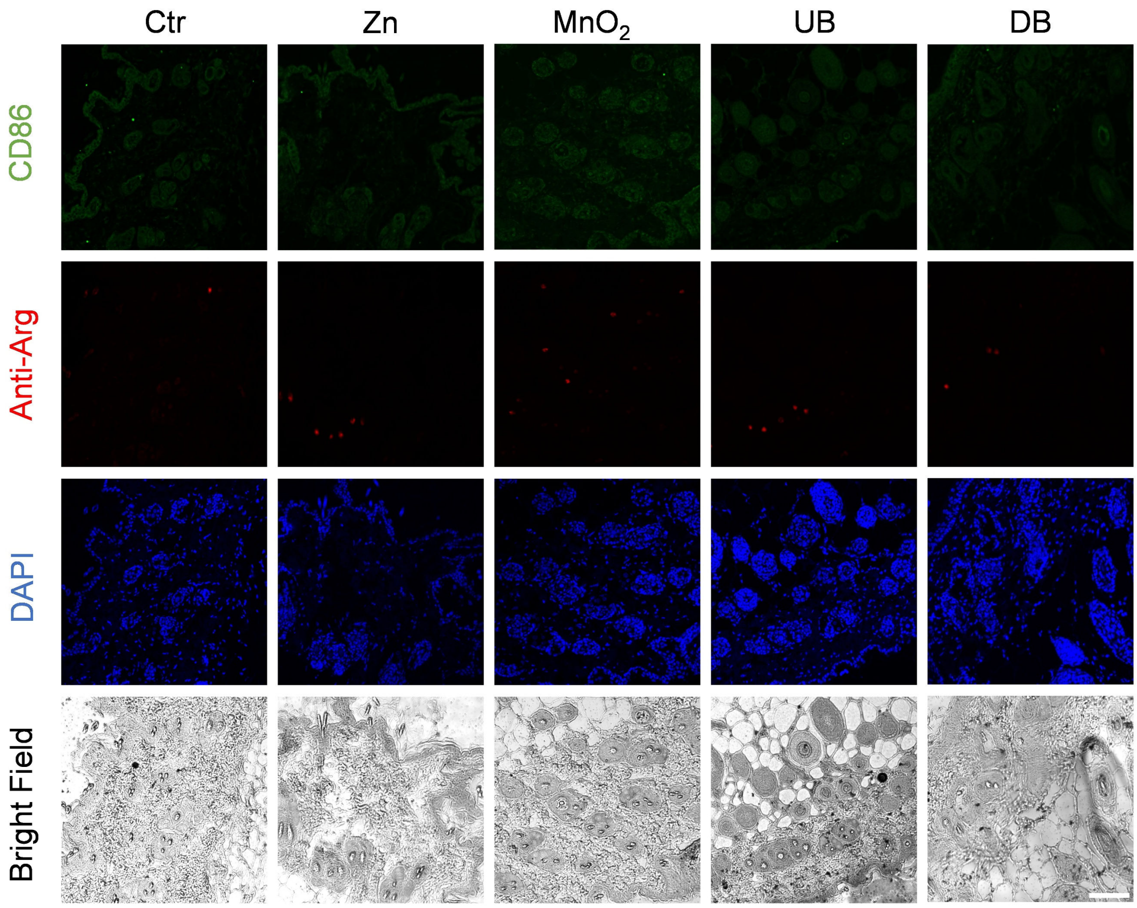

fig. S41. Representative images of immunofluorescence co-staining with the CD86, Arg antibody, and DAPI. Scale bar, 100 μm.

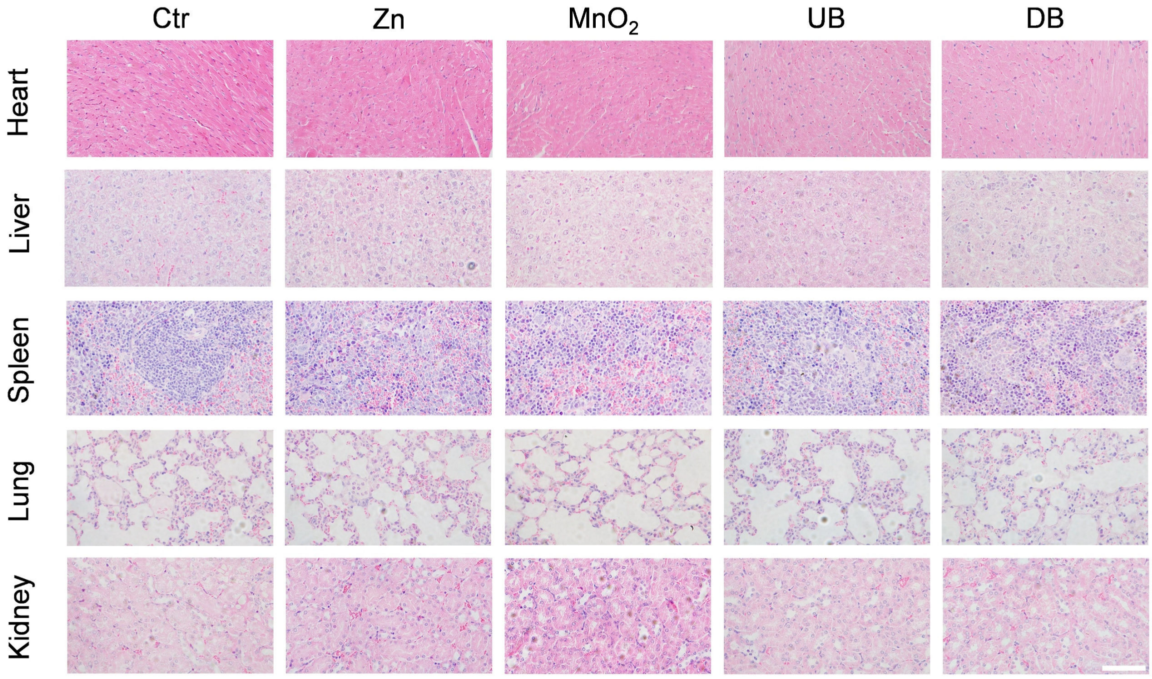

fig. S42. ***Ex vivo*** **H&E staining images of heart, liver, spleen, lung, and kidney tissues after being treated with various groups.** Scale bar, 100 µm.

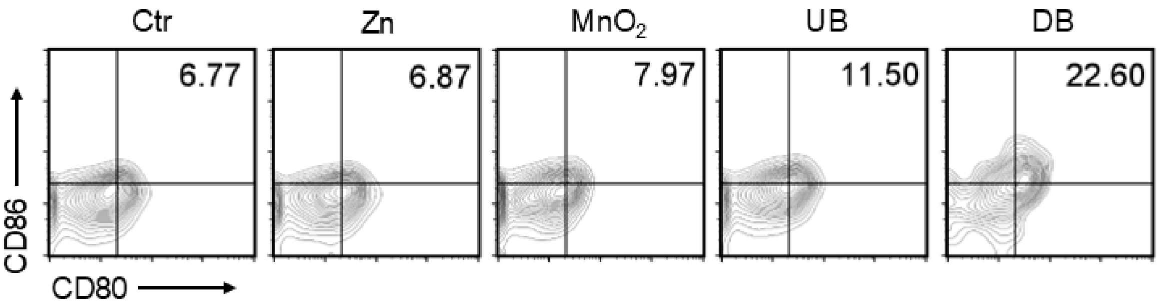

fig. S43. Representative flow cytometric analysis images of DC maturation in tumors cultured with various treatments.

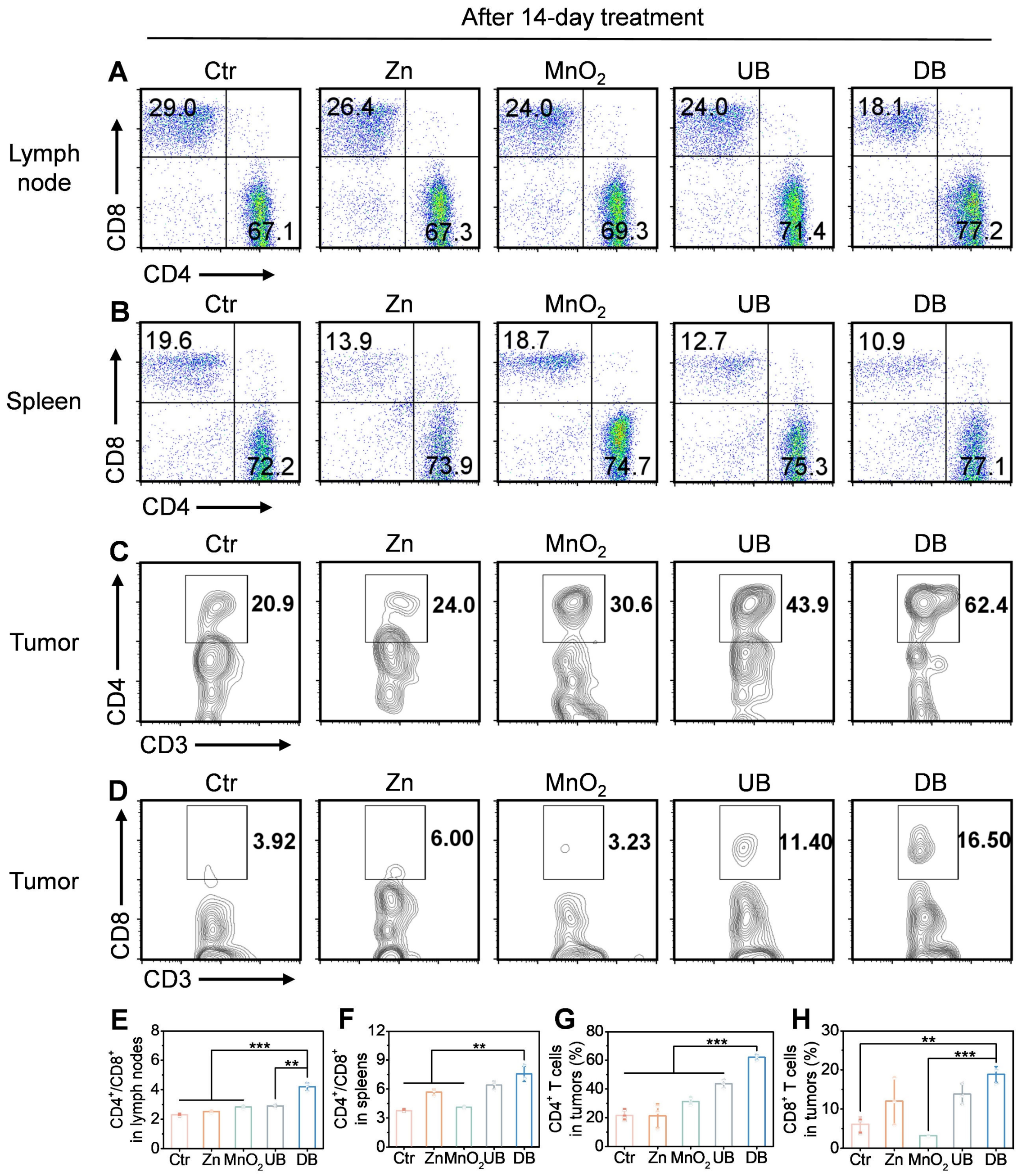

fig. S44. Flow cytometric analysis of T-cell infiltration after various treatments. (**A**) lymph nodes, (**B**) spleens, and (**C** and **D**) tumors. Histogram frequency of CD4$^+$/CD8$^+$ in (**E**) lymph nodes and (**F**) spleens. Histogram frequency of (**G**) CD4$^+$ T cells and (**H**) CD8$^+$ cytotoxic T cells in tumor-draining lymph nodes. All dates are expressed as mean ± SD (n = 3), and the differences were assessed by one-way ANOVA with Tukey's post-test. $^{**}p < 0.01$ and $^{***}p < 0.001$.

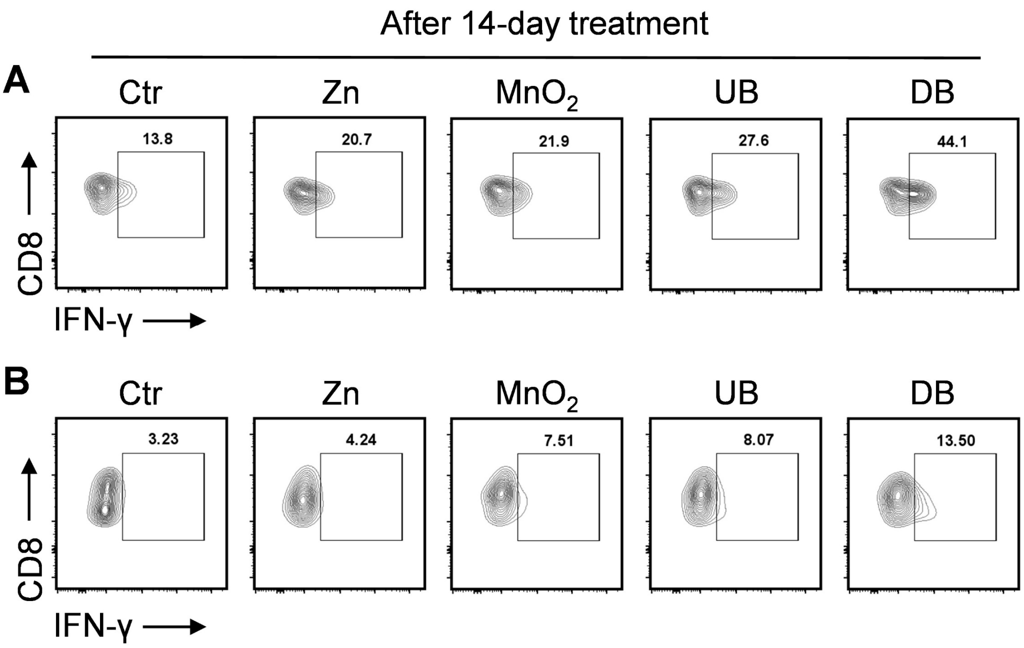

fig. S45. Expression of IFN-γ^+ in CD8$^+$ T cells after 14-day treatment with various groups detected by flow cytometry. (**A**) lymph nodes and (**B**) spleens.

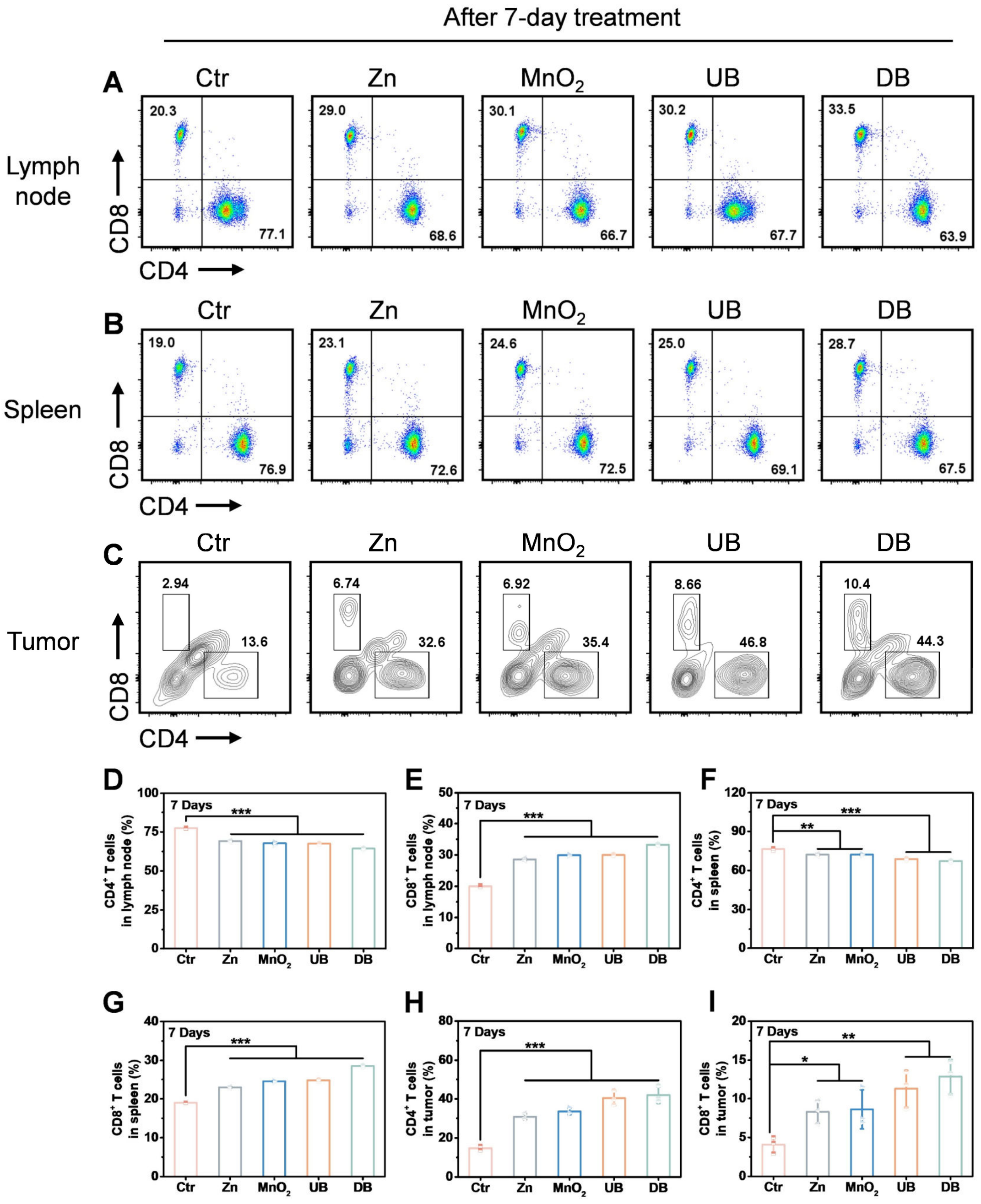

fig. S46. FCM analysis of CD4$^+$ T cells and CD8$^+$ T cells after 7-day treatments. (**A**) lymph nodes, (**B**) spleen, and (**C**) tumor after various treatments and (**D-I**) related quantification. All dates are expressed as mean ± SD (n = 3), and the differences were assessed by one-way ANOVA followed by Dunnett's multiple comparisons tests. *$p < 0.05$, **$p < 0.01$ and ***$p < 0.001$.

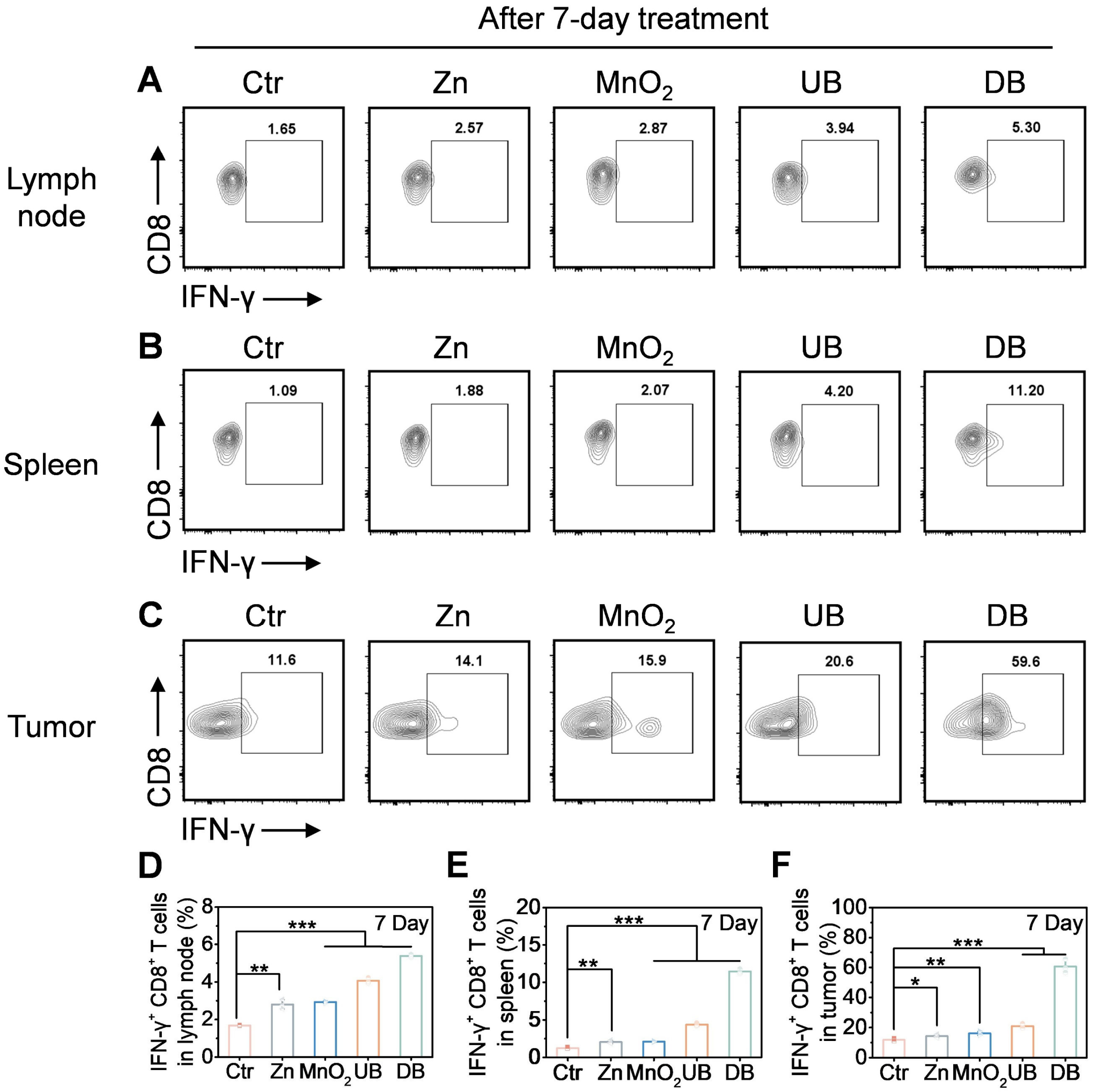

fig. S47. Expression of IFN-γ⁺ in CD8⁺ T cells after 7-day treatments detected by flow cytometry. (**A**) lymph nodes, (**B**) spleens, and (**C**) tumors. (**D-F**) Related quantification of IFN-γ⁺ T cells. All dates are expressed as mean ± SD (n = 3), and the differences were assessed by one-way ANOVA followed by Dunnett's multiple comparisons tests. $*p < 0.05$, $**p < 0.01$ and $***p < 0.001$.

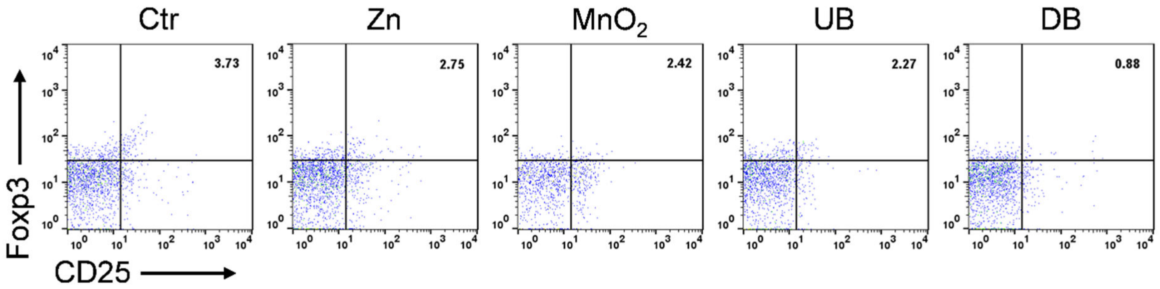

fig. S48. Flow cytometric analysis of Treg cells in tumors with various treatments.

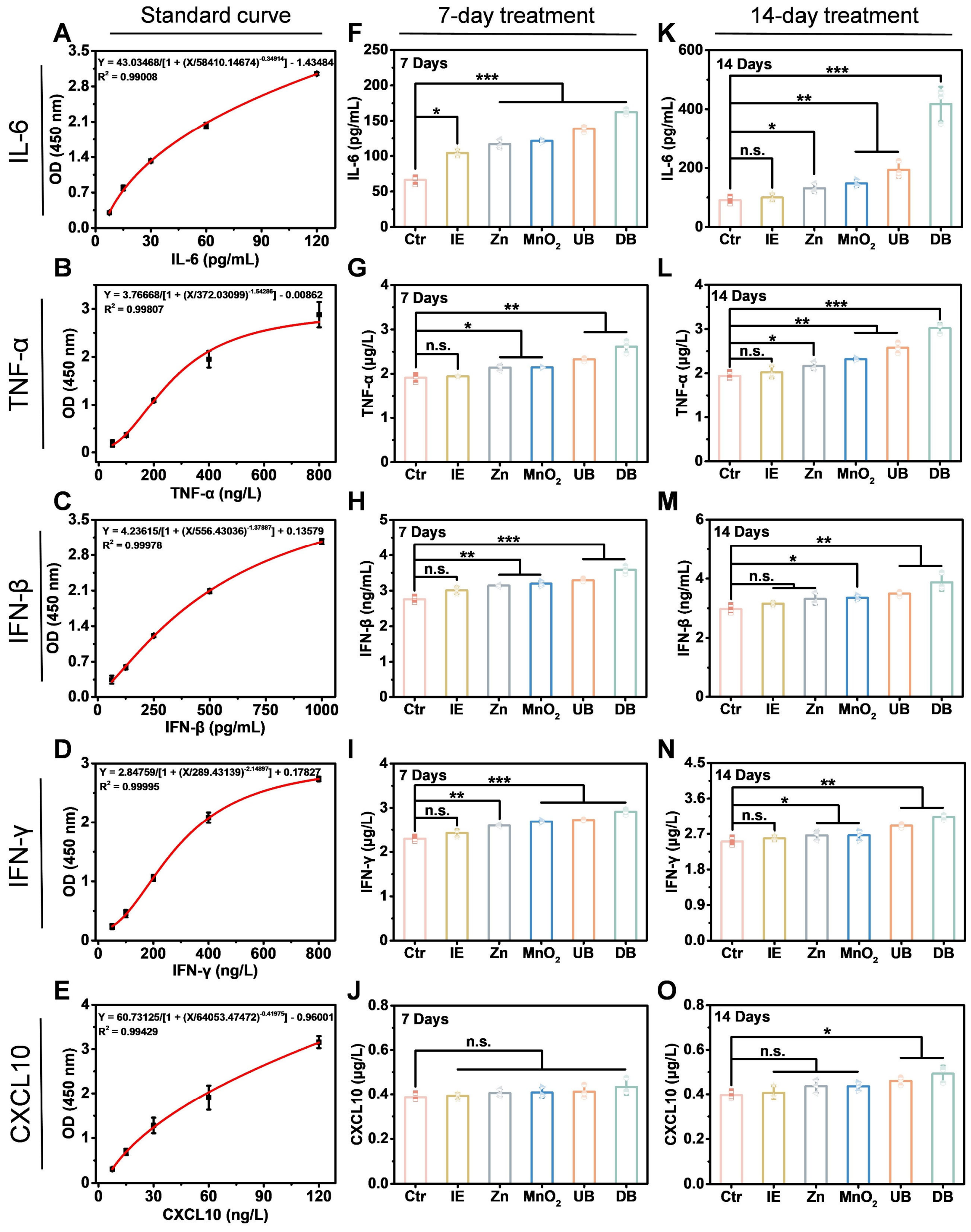

fig. S49. The secretion level of inflammatory cytokines (IL-6, TNF-α, IFN-β, IFN-γ, and CXCL10) in the serum of mice after being treated with various groups for 7 days and 14 days. (**A-E**) The standard curves. (**F-J**) The concentrations of inflammatory cytokines after 7-day treatments. (**K-O**) The concentrations of inflammatory cytokines after 14-day treatments. IE means the electrochemically inert electrode group. All data were expressed as mean ± SD (n = 3), and the

differences were assessed by one-way ANOVA followed by Dunnett's multiple comparisons tests. n.s.: no significance, $p > 0.05$, $^{*}p < 0.05$, $^{**}p < 0.01$ and $^{***}p < 0.001$.

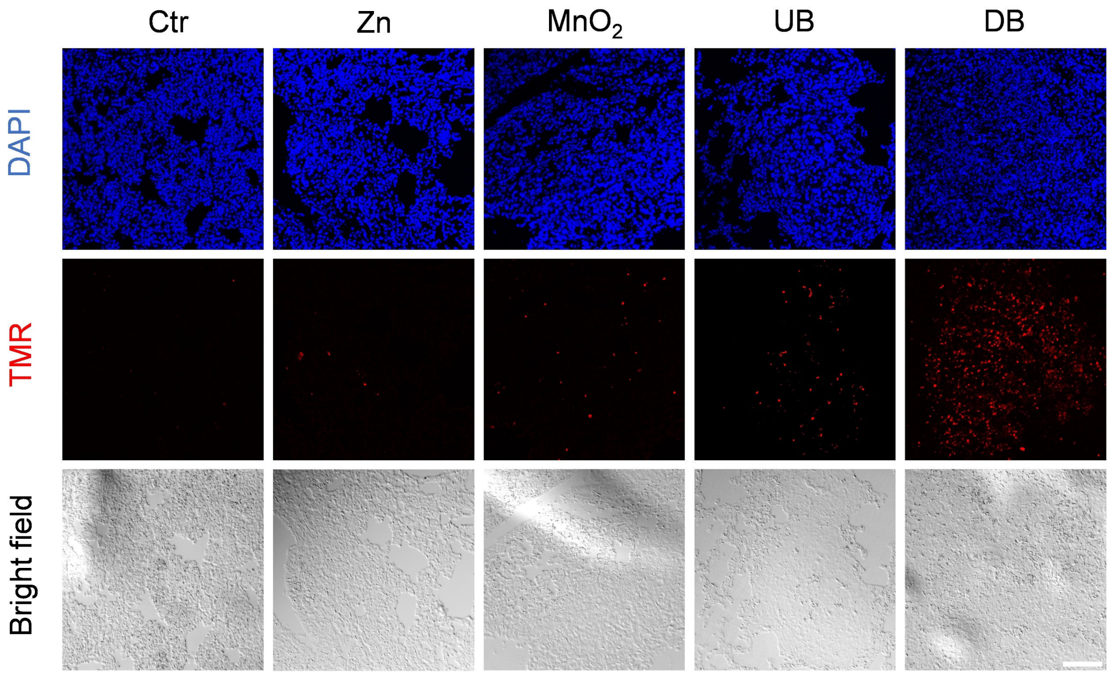

fig. S50. Representative confocal images of TUNEL in lungs after different treatments. Scale bar, 100 μm.

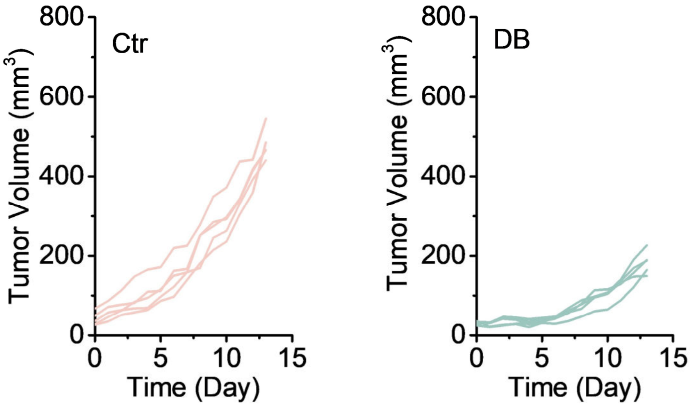

fig. S51. Tumor growth curves of each nude mouse after being treated with Ctr and DB group (n = 5).

table S1. Summary of current Zn or Mn ion delivery for cancer immunotherapy

Materials	Therapeutic approaches (Tumor model)	Treatment times	Material loading capacity	Drug (dose)	Inhibitory effect (day)	Ref
Manganese-enriched zinc peroxide nanoparticles	i.v. (BALB/c-4T1)	Three	Zn^{2+}: 23.4 µg/per	Anti-PD-1 (100 µg/per)	95.47% (14 days)	(*33*)
C_3N_4-Mn single-atom catalyst	i.v. (BALB/c nu-Hela)	Eight	C_3N_4: 100 µg/mL	/	62.40% (15 days)	(*56*)
Ultrathin Mn-based layered double hydroxide nanosheets	i.v. (C57BL/6-MC38)	Four	Mn: 6 mg/kg	/	81.40% (21 days)	(*34*)
	i.t. (C57BL/6-MC38)	Three		Anti-PD-L1 (2 mg/kg)	66.67% (21 days)	
Nanoparticles encapsulating MPLA and manganese tetroxide nanoparticles (PMM NPs)	i.v. (C57BL/6-MC38 and BALB/c-4T1)	Four	PMM: 75 µg/per Mn_3O_4: 20 µg/per MPLA: 5 µg/per	Anti-PD-1 (100 µg/per)	60.00% (21 days)	(*38*)
					/	
Nanofibrous hydrogel (MnP^{gel})	i.m. (C57BL/6)	One	Mn: 200 µg/per or 400 µg/per	/	/	(*36*)
Manganese-doped layered double hydroxide nanoparticles (OVA/Mn-LDH)	s.c. (C57BL/6-B16-OVA)	Two	Mn: 1 mg/kg OVA: 25 mg/kg	/	86.20% (10 days)	(*35*)
PEGylated manganese-doped calcium sulfide nanoparticles	i.t. (BALB/c-U14)	Three	MCSP: 5 mg/kg	/	/	(*57*)
				Anti-PD-1 (5 mg/kg)	/	
Sulfate radical (SO^{4-}) based in situ vaccine	i.t. (BALB/c-4T1)	Three	25 µg/mL	/	/	(*58*)
				Anti-PD-L1	/	
Monodispersed PtMnIr nanozymes	i.v. + Electric field application (BALB/c-B16-F10)	Two	PtMnIr: 20 mg/kg	/	/	(*59*)
Mn-doped potassium chloride nanoelectrodes (MPC NEs)	i.t. + Electric field application (BALB/c-4T1)	Three	PMPC: 5mg/kg	/	91.00% (14 days)	(*41*)
				Anti-CTLA-4 (20 µg/per)	/	
**Zn//MnO_2 battery**	**Orthotopic implantation (BALB/c-4T1)**	**One**	**Mn: 3 mg/per (150 mg/kg)**	/	**99.63% (14 days)**	**This work**

Notes: i.v.: intravenous injection; i.t.: intratumoral injection; i.m.: intramuscular injection; s.c.: subcutaneous injection.

table S2. Summary of current electrical immunotherapy

Materials	Therapeutic approaches	Treatment times	Material loading capacity	Drug (dose)	Inhibitory effect	Ref.
Direct-current triboelectric nanogenerator	Connected with external material (BALB/c-4T1)	Six	/	/	/	(*60*)
Nanoscale galvanic cell (NanoGC)	i.t. (BALB/c-4T1)	Two	NanoGC: 4 mg/kg<br>NanoGC: 7 mg/kg	/	/	(*61*)
**Zn//MnO_2 battery**	**Orthotopic implantation (BALB/c-4T1)**	**One**	**Mn: 3 mg/per (150 mg/kg)**	/	**99.63% (14 days)**	**This work**

Notes: i.t.: intratumoral injection.

REFERENCES AND NOTES

1. Y. Chao, Z. Liu, Biomaterials tools to modulate the tumour microenvironment in immunotherapy. *Nat. Rev. Bioeng.* **1**, 125–138 (2023).

2. Z. Chen, C. Meng, J. Mai, Y. Liu, H. Li, H. Shen, An mRNA vaccine elicits STING-dependent antitumor immune responses. *Acta Pharm. Sin. B* **13**, 1274–1286 (2023).

3. X. Xue, H. Qu, Y. Li, Stimuli-responsive crosslinked nanomedicine for cancer treatment. *Exp. Dermatol.* **2**, 20210134 (2022).

4. S. K. Alsaiari, S. S. Qutub, S. Sun, W. Baslyman, M. Aldehaiman, M. Alyami, A. Almalik, R. Halwani, J. Merzaban, Z. Mao, N. M. Khashab, Sustained and targeted delivery of checkpoint inhibitors by metal-organic frameworks for cancer immunotherapy. *Sci. Adv.* **7**, eabe7174 (2021).

5. Z. Deng, M. Xi, C. Zhang, X. Wu, Q. Li, C. Wang, H. Fang, G. Sun, Y. Zhang, G. Yang, Z. Liu, Biomineralized MnO_2 nanoplatforms mediated delivery of immune checkpoint inhibitors with STING pathway activation to potentiate cancer radio-immunotherapy. *ACS Nano* **17**, 4495–4506 (2023).

6. C. Zhang, J. Huang, Z. Zeng, S. He, P. Cheng, J. Li, K. Pu, Catalytical nano-immunocomplexes for remote-controlled sono-metabolic checkpoint trimodal cancer therapy. *Nat. Commun.* **13**, 3468 (2022).

7. B. Choi, H. Choi, H. Kim, A. Choi, S.-W. Kwon, S. K. Mouli, R. J. Lewandowski, D.-H. Kim, Z-domain protein nano-bio interfaced MRI visible anti-program death ligand-1 nanoconjugates for enhanced local immune checkpoint inhibitor immunotherapy. *Nano Today* **45**, 101552 (2022).

8. X. Xu, Z. Zhang, J. Du, Y. Xue, X. Chen, J. Zhang, X. Yang, D. Chang, J. Xie, S. Ju, Recruiting T-cells toward the brain for enhanced glioblastoma immunotherapeutic efficacy by co-delivery of cytokines and immune checkpoint antibodies with macrophage-membrane-camouflaged nanovesicles. *Adv. Mater.* **35**, e2209785 (2023).

9. Y. Agarwal, L. E. Milling, J. Y. H. Chang, L. Santollani, A. Sheen, E. A. Lutz, A. Tabet, J. Stinson, K. Ni, K. A. Rodrigues, T. J. Moyer, M. B. Melo, D. J. Irvine, K. D. Wittrup, Intratumourally injected alum-tethered cytokines elicit potent and safer local and systemic anticancer immunity. *Nat. Biomed. Eng.* **6**, 129–143 (2022).

10. A. M. Nash, M. I. Jarvis, S. Aghlara-Fotovat, S. Mukherjee, A. Hernandez, A. D. Hecht, P. D. Rios, S. Ghani, I. Joshi, D. Isa, Y. Cui, S. Nouraein, J. Z. Lee, C. Xu, D. Y. Zhang, R. A. Sheth, W. Peng, J. Oberholzer, O. A. Igoshin, A. A. Jazaeri, O. Veiseh, Clinically translatable cytokine delivery platform for eradication of intraperitoneal tumors. *Sci. Adv.* **8**, eabm1032 (2022).

11. S. Liang, J. Yao, D. Liu, L. Rao, X. Chen, Z. Wang, Harnessing nanomaterials for cancer sonodynamic immunotherapy. *Adv. Mater.* **35**, e2211130 (2023).

12. M. Zhou, S. Liang, D. Liu, K. Ma, Y. Peng, Z. Wang, Engineered nanoprobes for immune activation monitoring. *ACS Nano* **16**, 19940–19958 (2022).

13. G. Ji, L. Ma, H. Yao, S. Ma, X. Si, Y. Wang, X. Bao, L. Ma, F. Chen, C. Ma, L. Huang, X. Fang, W. Song, Precise delivery of obeticholic acid via nanoapproach for triggering natural killer T cell-mediated liver cancer immunotherapy. *Acta Pharm. Sin. B* **10**, 2171–2182 (2020).

14. C. Ding, Z. Song, A. Shen, T. Chen, A. Zhang, Small molecules targeting the innate immune cGAS–STING–TBK1 signaling pathway. *Acta Pharm. Sin. B* **10**, 2272–2298 (2020).

15. A. Decout, J. D. Katz, S. Venkatraman, A. Ablasser, The cGAS–STING pathway as a therapeutic target in inflammatory diseases. *Nat. Rev. Immunol.* **21**, 548–569 (2021).

16. J. Kwon, S. F. Bakhoum, The cytosolic DNA-sensing cGAS–STING pathway in cancer. *Cancer Discov.* **10**, 26–39 (2020).

17. M. Jiang, P. Chen, L. Wang, W. Li, B. Chen, Y. Liu, H. Wang, S. Zhao, L. Ye, Y. He, C. Zhou, cGAS-STING, an important pathway in cancer immunotherapy. *J. Hematol. Oncol.* **13**, 81 (2020).

18. X. Kong, H. Zuo, H.-D. Huang, Q. Zhang, J. Chen, C. He, Y. Hu, STING as an emerging therapeutic target for drug discovery: Perspectives from the global patent landscape. *J. Adv. Res.* **44**, 119–133 (2023).

19. K.-P. Hopfner, V. Hornung, Molecular mechanisms and cellular functions of cGAS–STING signalling. *Nat. Rev. Mol. Cell Bio.* **21**, 501–521 (2020).

20. L. Deng, H. Liang, M. Xu, X. Yang, B. Burnette, A. Arina, X.-D. Li, H. Mauceri, M. Beckett, T. Darga, X. Huang, T. F. Gajewski, Z. J. Chen, Y.-X. Fu, R. R. Weichselbaum, STING-dependent cytosolic DNA sensing promotes radiation-induced type I interferon-dependent antitumor immunity in immunogenic tumors. *Immunity* **41**, 843–852 (2014).

21. R. Zhang, C. Wang, Y. Guan, X. Wei, M. Sha, M. Yi, M. Jing, M. Lv, W. Guo, J. Xu, Y. Wan, X.-M. Jia, Z. Jiang, Manganese salts function as potent adjuvants. *Cell. Mol. Immunol.* **18**, 1222–1234 (2021).

22. X. Sun, Y. Zhang, J. Li, K. S. Park, K. Han, X. Zhou, Y. Xu, J. Nam, J. Xu, X. Shi, L. Wei, Y. L. Lei, J. J. Moon, Amplifying STING activation by cyclic dinucleotide–manganese particles for local and systemic cancer metalloimmunotherapy. *Nat. Nanotechnol.* **16**, 1260–1270 (2021).

23. C. Wang, Y. Guan, M. Lv, R. Zhang, Z. Guo, X. Wei, X. Du, J. Yang, T. Li, Y. Wan, X. Su, X. Huang, Z. Jiang, Manganese increases the sensitivity of the cGAS-STING pathway for double-stranded DNA and is required for the host defense against DNA viruses. *Immunity* **48**, 675–687.e7 (2018).

24. M. Lv, M. Chen, R. Zhang, W. Zhang, C. Wang, Y. Zhang, X. Wei, Y. Guan, J. Liu, K. Feng, M. Jing, X. Wang, Y.-C. Liu, Q. Mei, W. Han, Z. Jiang, Manganese is critical for antitumor immune responses via cGAS-STING and improves the efficacy of clinical immunotherapy. *Cell Res.* **30**, 966–979 (2020).

25. J. Li, H. Ren, Q. Qiu, X. Yang, J. Zhang, C. Zhang, B. Sun, J. F. Lovell, Y. Zhang, Manganese coordination micelles that activate stimulator of interferon genes and capture in situ tumor antigens for cancer metalloimmunotherapy. *ACS Nano* **16**, 16909–16923 (2022).

26. N. Fan, K. Chen, R. Zhu, Z. Zhang, H. Huang, S. Qin, Q. Zheng, Z. He, X. He, W. Xiao, Y. Zhang, Y. Gu, C. Zhao, Y. Liu, X. Jiang, S. Li, Y. Wei, X. Song, Manganese-coordinated mRNA vaccines with enhanced mRNA expression and immunogenicity induce robust immune responses against SARS-CoV-2 variants. *Sci. Adv.* **8**, eabq3500 (2022).

27. X. Sun, X. Zhou, X. Shi, O. A. Abed, X. An, Y. L. Lei, J. J. Moon, Strategies for the development of metalloimmunotherapies. *Nat. Biomed. Eng.* **8**, 1073–1091 (2024).

28. B. Ding, P. Zheng, F. Jiang, Y. Zhao, M. Wang, M. Chang, P. a. Ma, J. Lin, MnO_x nanospikes as nanoadjuvants and immunogenic cell death drugs with enhanced antitumor immunity and antimetastatic effect. *Angew. Chem. Int. Ed.* **59**, 16381–16384 (2020).

29. Y. Wang, Y. Li, Z. Zhang, L. Wang, D. Wang, B. Z. Tang, Triple-jump photodynamic theranostics: MnO_2 combined upconversion nanoplatforms involving a type-I photosensitizer with aggregation-induced emission characteristics for potent cancer treatment. *Adv. Mater.* **33**, e2103748 (2021).

30. J. Ou, H. Tian, J. Wu, J. Gao, J. Jiang, K. Liu, S. Wang, F. Wang, F. Tong, Y. Ye, L. Liu, B. Chen, X. Ma, X. Chen, F. Peng, Y. Tu, MnO_2-based nanomotors with active fenton-like Mn^{2+} delivery for enhanced chemodynamic therapy. *ACS Appl. Mater. Interfaces* **13**, 38050–38060 (2021).

31. S. Chattopadhyay, Y.-H. Liu, Z.-S. Fang, C.-L. Lin, J.-C. Lin, B.-Y. Yao, C.-M. J. Hu, Synthetic immunogenic cell death mediated by intracellular delivery of STING agonist nanoshells enhances anticancer chemo-immunotherapy. *Nano Lett.* **20**, 2246–2256 (2020).

32. S. Ma, W. Song, Y. Xu, X. Si, S. Lv, Y. Zhang, Z. Tang, X. Chen, Rationally designed polymer conjugate for tumor-specific amplification of oxidative stress and boosting antitumor immunity. *Nano Lett.* **20**, 2514–2521 (2020).

33. M. L. Zhou, S. Liang, D. Liu, K. S. Ma, K. Q. Yun, J. J. Yao, Y. X. Peng, L. N. Hai, Q. Zhang, Z. H. Wang, Manganese-enriched zinc peroxide functional nanoparticles for potentiating cancer immunotherapy. *Nano Lett.* **23**, 10350–10359 (2023).

34. J. P. Liu, J. Z. Zhan, Y. Zhang, L. Huang, J. Yang, J. Feng, L. W. Ding, Z. Y. Shen, X. Y. Chen, Ultrathin clay nanoparticles-mediated mutual reinforcement of ferroptosis and cancer immunotherapy. *Adv. Mater.* **36**, e2309562 (2024).

35. J. Liu, Y. Zhang, B. Yang, Y. Jia, R. T. Liu, L. Ding, Z. Shen, X. Chen, Synergistic glutathione depletion and STING activation to potentiate dendritic cell maturation and cancer vaccine efficacy. *Angew. Chem. Int. Ed.* **63**, e202318530 (2024).

36. H. Jia, J. Lin, D. Wang, X. Lv, Q. Wang, Z. Wang, J. Liu, L. Yang, J. Liu, A Mn^{2+}-assisted nanofiber-hydrogel adjuvant for simultaneous enhancement of humoral and cellular immune responses. *Adv. Funct. Mater.* **34**, 2315442 (2024).

37. H. Zhong, G. Chen, T. Li, J. Huang, M. Lin, B. Li, Z. Xiao, X. Shuai, Nanodrug augmenting antitumor immunity for enhanced TNBC therapy via pyroptosis and cGAS-STING activation. *Nano Lett.* **23**, 5083–5091 (2023).

38. D. Liu, S. Liang, K. Ma, Q. F. Meng, X. Li, J. Wei, M. Zhou, K. Yun, Y. Pan, L. Rao, X. Chen, Z. Wang, Tumor microenvironment-responsive nanoparticles amplifying STING signaling pathway for cancer immunotherapy. *Adv. Mater.* **36**, 2304845 (2023).

39. X. Li, S. Khorsandi, Y. Wang, J. Santelli, K. Huntoon, N. Nguyen, M. Yang, D. Lee, Y. Lu, R. Gao, B. Y. S. Kim, C. de Gracia Lux, R. F. Mattrey, W. Jiang, J. Lux, Cancer immunotherapy based on image-guided STING activation by nucleotide nanocomplex-decorated ultrasound microbubbles. *Nat. Nanotechnol.* **17**, 891–899 (2022).

40. R. Verbeke, I. Lentacker, L. Wayteck, K. Breckpot, M. Van Bockstal, B. Descamps, C. Vanhove, S. C. De Smedt, H. Dewitte, Co-delivery of nucleoside-modified mRNA and TLR agonists for cancer immunotherapy: Restoring the immunogenicity of immunosilent mRNA. *J. Control. Release* **266**, 287–300 (2017).

41. G. Wang, J. Li, L. Wang, Y. Yang, J. Wu, W. Tang, H. Lei, L. Cheng, Manganese-doped potassium chloride nanoelectrodes to potentiate electrochemical immunotherapy. *ACS Nano* **18**, 10885–10901 (2024).

42. J. Huang, P. Yu, M. Liao, X. Dong, J. Xu, J. Ming, D. Bin, Y. Wang, F. Zhang, Y. Xia, A self-charging salt water battery for antitumor therapy. *Sci. Adv.* **9**, eadf3992 (2023).

43. H. Chen, H. Kuang, F. Liu, Y. Wu, S. Cai, M. Xu, S.-J. Bao, A self-healing neutral aqueous rechargeable Zn/MnO_2 battery based on modified carbon nanotubes substrate cathode. *J. Colloid Interf. Sci.* **600**, 83–89 (2021).

44. X. Guo, J. Zhou, C. Bai, X. Li, G. Fang, S. Liang, Zn/MnO_2 battery chemistry with dissolution-deposition mechanism. *Mater. Today Energy* **16**, 100396 (2020).

45. S. Liu, T. Zeng, Z. He, M. Zuo, S. Chen, Y. Liu, Z. Fan, H. He, Q. Kong, Z. Zhou, L. Han, Synergistic effect of Ru single atoms and MnO_2 to boost oxygen reduction/evolution activity via strong electronic interaction. *Chem. Eng. J.* **499**, 156051 (2024).

46. J. Xie, Y. Chen, Z. He, S. Liu, Y. Liu, B. Li, T. Xu, X. Ning, S. Chen, T. Zeng, H. He, Single-atom Ni anchored on α-MnO_2 nanorods as an electrocatalyst for the oxygen evolution and oxygen reduction reactions. *ACS Appl. Nano Mater.* **7**, 18027–18035 (2024).

47. Z.-L. He, L.-Q. Wang, M. Jiang, J.-N. Xie, S. Liu, J.-C. Ren, R. Sun, W.-B. Lv, W.-B. Guo, Y.-L. Liu, B. Li, Q. Liu, H. He, Surface engineering on MnO_2 nanorods by La single atoms to accelerate oxygen reduction kinetics. *Rare Met.* **43**, 4302–4311 (2024).

48. J. Lei, Y. Yao, Z. Wang, Y.-C. Lu, Towards high-areal-capacity aqueous zinc–manganese batteries: Promoting MnO_2 dissolution by redox mediators. *Energ. Environ. Sci.* **14**, 4418–4426 (2021).

49. W. Chen, G. Li, A. Pei, Y. Li, L. Liao, H. Wang, J. Wan, Z. Liang, G. Chen, H. Zhang, J. Wang, Y. Cui, A manganese–hydrogen battery with potential for grid-scale energy storage. *Nat. Energy* **3**, 428–435 (2018).

50. E. Mylod, G. Conlon, E. P. W. Jenkins, G. G. Malliaras, C. M. Gardiner, Tumor-treating fields increase cytotoxic degranulation of natural killer cells against cancer cells. *Cell Rep. Phys. Sci.* **5**, 102119 (2024).

51. Y. Wang, F. Gao, L. Zhao, Y. Wu, C. Li, H. Li, Y. Jiang, Enhancing cancer treatment via "Zn^{2+} interference" with Zn-based nanomaterials. *Coord. Chem. Rev.* **500**, 215535 (2024).

52. L. Ding, M. Liang, Y. Li, M. Zeng, M. Liu, W. Ma, F. Chen, C. Li, R. L. Reis, F. R. Li, Y. Wang, Zinc-organometallic framework vaccine controlled-release Zn^{2+} regulates tumor extracellular matrix degradation potentiate efficacy of immunotherapy. *Adv. Sci.* **10**, 2302967 (2023).

53. X. Zeng, Z. Wang, A. Zhao, Y. Wu, Z. Wang, A. Wu, Q. Wang, X. Xia, X. Chen, W. Zhao, B. Li, Z. Lu, Q. Lv, G. Li, Z. Zuo, F. Wu, Y. Zhao, T. Wang, G. Nie, S. Li, G. Zhang, Zinc nanoparticles from oral supplements accumulate in renal tumours and stimulate antitumour immune responses. *Nat. Mater.* **24**, 287–296 (2025).

54. Y. Lv, X. Liu, J. Liu, S. Wu, S. Sun, P. Wu, Y. Wang, Y. Ding, Implantable and bio-compatible Na-O_2 battery. *Chem* **10**, 1885–1896 (2024).

55. H. Wu, Y. Wang, H. Li, Y. Hu, Y. Liu, X. Jiang, H. Sun, F. Liu, A. Xiao, T. Chang, L. Lin, K. Yang, Z. Wang, Z. Dong, Y. Li, S. Dong, S. Wang, J. Chen, Y. Liu, D. Yin, H. Zhang, M. Liu, S. Kong, Z. Yang, X. Yu, Y. Wang, Y. Fan, L. Wang, C. Yu, L. Chang, Accelerated intestinal wound healing via dual electrostimulation from a soft and biodegradable electronic bandage. *Nat. Electron.* **7**, 299–312 (2024).

56. Y. Yin, X. Ge, J. Ouyang, N. Na, Tumor-activated in situ synthesis of single-atom catalysts for O_2-independent photodynamic therapy based on water-splitting. *Nat. Commun.* **15**, 2954 (2024).

57. L. Liu, H. Lei, G. Hou, L. Zhang, Y. Chen, Y. Lu, Z. Pei, J. Ge, J. Wu, J. Zhou, L. Cheng, Gas-amplified metalloimmunotherapy with dual activation of pyroptosis and the STING pathway for remodeling the immunosuppressive cervical cancer microenvironment. *ACS Nano* **18**, 12830–12844 (2024).

58. Y. Huang, J. Zou, J. Huo, M. Zhang, Y. Yang, Sulfate radical based in situ vaccine boosts systemic antitumor immunity via concurrent activation of necroptosis and STING pathway. *Adv. Mater.* **36**, e2407914 (2024).

59. D. Li, E. Ha, Z. Zhou, J. Zhang, Y. Zhu, F. Ai, L. Yan, S. He, L. Li, J. Hu, “Spark” PtMnIr nanozymes for electrodynamic-boosted multienzymatic tumor immunotherapy. *Adv. Mater.* **36**, e2308747 (2024).

60. H. Li, C. Chen, Z. Wang, Y. Huang, G. He, Y. Liu, P. Jiang, Z. L. Wang, Triboelectric immunotherapy using electrostatic-breakdown induced direct-current. *Mater. Today* **64**, 40–51 (2023).

61. W. Q. Huang, Y. Q. Zhu, F. Gao, W. You, G. Chen, X. Nie, L. Xia, L. H. Wang, C. Y. Hong, Z. Zhang, F. Wang, Y. Yu, Y. Z. You, Nanogalvanic cells release highly reactive electrons in tumors to effectively eliminate tumors. *Adv. Mater.* **36**, 2404199 (2024).
